# Synthesis and characterization of novel hercynite@sulfuric acid and its catalytic applications in the synthesis of polyhydroquinolines and 2,3-dihydroquinazolin-4(1*H*)-ones†

**DOI:** 10.1039/d1ra07381h

**Published:** 2022-01-20

**Authors:** Masoud Mohammadi, Arash Ghorbani-Choghamarani

**Affiliations:** Department of Chemistry, Faculty of Science, Ilam University P.O. Box 69315516 Ilam Iran; Department of Organic Chemistry, Faculty of Chemistry, Bu–Ali Sina University Hamedan 6517838683 Iran arashghch58@yahoo.com a.ghorbani@basu.ac.ir +98 8138380709 +98 8138282807

## Abstract

Herein, we report the synthesis of hercynite@sulfuric acid as a novel nanomagnetic solid acid catalyst, containing the sulfuric acid catalytic sites on the surface of hercynite MNPs as the catalytic support. The as-synthesized nanocomposite was meticulously characterized using a wide range of physicochemical techniques; including, FT-IR, XRD, EDX, X-ray-mapping, SEM and VSM analysis. The catalytic activity of this nanomagnetic material was considered for the synthesis of the diversely substituted polyhydroquinolines and 2,3-dihydroquinazolin-4(1*H*)-ones under solvent free conditions and also cyclocondensation reactions in ethanol, respectively affording good to excellent yields. Moreover, it is worth mentioning that the heterogeneity of the catalyst was measured through its excellent reusability and hot-filtration test.

## Abstract 2

Herein, we report the synthesis of hercynite@sulfuric acid as a novel nanomagnetic solid acid catalyst, containing the sulfuric acid catalytic sites on the surface of hercynite MNPs as the catalytic support.

## Graphical abstract

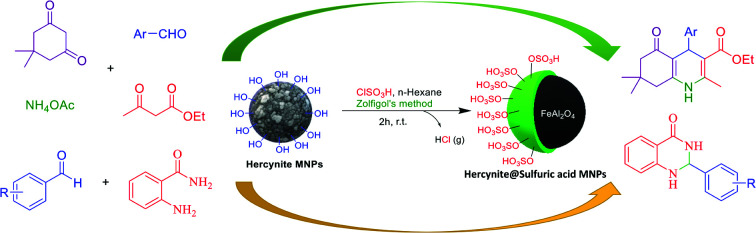

## 1. Introduction

Multicomponent reactions have attracted extensive attention due to the fact that they offer a fundamental methodology in organic synthesis.^1–4^ This synthetic method has been utilized to prepare a variety of N-containing heterocycles, which are interesting drugs and useful organic intermediates in synthetic organic chemistry.^5–9^ In this regard, dihydropyridines and 2,3-dihydroquinazolin-4(1*H*)-ones are important classes of heterocycles having high nitrogen content.^6,10,11^ Moreover, they are frequently used as bioisosteres of pyridines exhibiting a plethora of applications in pharmaceuticals and drug designing as explosives and also in material science as important precursors in synthetic chemistry for the synthesis of other nitrogenous heterocycles.^12,13^ Catalytic Hantzsch synthesis which involves the condensation of an aldehyde, two β-keto ester constituents and a nitrogen donor is the most useful synthetic method in order to generate polyhydroquinolines which are a class of dihydropyridine-containing compounds.^6,14–16^ Although there are numerous reports on the synthesis of 2,3-dihydroquinazolin-4(1*H*)-one derivatives,^10,12^ the clean, one pot and green synthetic methodologies for these molecules are rather limited. An interesting protocol involves the cyclocondensation of aryl aldehydes with anthranilamide towards a library of 2,3-dihydroquinazolin-4(1*H*)-ones.^17–19^ In this method, anthranilamide is considered as a green reagent because, on the basis of the cyclic mechanism, this reaction gives only water as the by-product.^20^

In order to derive the mentioned reactions, acid catalysts which dominate these reactions can be regarded as standard methods, evaluating the reactivity of Brønsted–Lowry acid species as potential catalysts.^21–23^ These catalysts are generally classified into two groups: homogeneous and heterogeneous acidic catalysts.^22,24–27^ There are numerous organic and inorganic homogeneous materials, which act as the Lewis or Brønsted–Lowry acids and are efficient in organic reactions.^28–32^ On the other hand, in recent years, there has been developed a great interest in using heterogeneous (solid) acid catalysts instead of those homogeneous ones, due to their possible recovery and recycling solids, which results in reducing the environmental impact.^5,6,22^ Some applications of solid acids in organic synthesis have been reviewed by Niknam *et al.* in 2020.^24^

Most solid state acids are heterogenized organic acids and transition metal complexes or acidic ion-exchange polymer resins.^33–36^ Recently, various types of solid-acid catalysts, *i.e.* silica sulfuric acid (SSA),^37,38^ boehmite silica sulfuric acid (boehmite-SSA)^39^ and magnetic silica sulfuric acid (Fe_3_O_4_@SSA),^40^ have been developed using Zolfigol's method. Like various types of catalysts, their configuration has some specific advantages and disadvantages.^5^

The magnetically recoverable catalysts have attracted a wide variety of attention due to the fact that the magnetic separation is economic and green.^41–43^ The FeAl_2_O_4_ spinel ferrite hercynite is another novel promising nano-material which has been examined in our previous works as an efficient heterogeneous catalytic support for the immobilization of homogeneous catalytic species.^44^ Nanomagnetic hercynite's ability to operate as a Lewis acid catalyst makes it a commercially attractive material because it reduces the cost of synthesis of active heterogeneous catalysts material and minimizes the synthetic steps and wastes.^45^

Given the mentioned features, we have developed the hercynite MNPs as a novel heterogeneous support for the immobilization of sulfuric acid to generate a novel magnetic solid acid catalyst. According to the best of our knowledge, this is the first report on the immobilization of the sulfuric acid (SO_3_H) groups by Zolfigol's method on the surface of hercynite as nanomagnetic catalytic support to get a novel solid acid catalyst.

Herein, hercynite@sulfuric acid MNPs are introduced as a novel heterogeneous catalytic system for the synthesis of diversely substituted polyhydroquinolines and 2,3-dihydroquinazolin-4(1*H*)-ones under green conditions.

## 2. Experimental

### 2.1. Preparation of sulfuric acid supported on the surface of hercynite magnetic nanoparticles (hercynite@sulfuric acid)

The hercynite magnetic nanoparticles were prepared using the coprecipitation technique, as it was previously reported by our group.^45^ Afterwards, its surface was coated with sulfuric acid catalytic cites according to the Zolfigol's method: 1 g of the prepared hercynite MNPS was dispersed in dry *n*-hexane (10 mL) by sonication for 30 min. Subsequently, chlorosulfuric acid (1.5 mL) was added dropwise over a period of 30 min and, then, the mixture was stirred for 4 h at room temperature. Afterwards, the obtained hercynite@sulfuric acid MNPs were separated using an external magnet, washed by dry *n*-hexane, respectively, to remove the unattached substrates and, finally, dried at 80 °C in an oven for 12 h.

### 2.2. General procedure for the catalytic synthesis of polyhydroquinolines

A mixture of aromatic aldehydes (1.0 mmol), ethyl acetoacetate (1 mmol), dimedone (1 mmol), NH_4_OAc (1.2 mmol) and hercynite@sulfuric acid (7 mg) was stirred at 100 °C under solvent-free conditions for the required time. The progress of reaction was monitored by TLC. After completion of the reaction, the reaction mixture was diluted with hot ethanol to dissolve the organic products. Afterwards, the catalyst was collected using magnetic decantation and, then, washed with hot ethyl acetate and water. Finally, the pure polyhydroquinoline products were obtained through recrystallization in ethanol.

### 2.3. General procedure for the catalytic synthesis of 2,3-dihydroquinazolin-4(1*H*)-ones

A mixture of aromatic aldehydes (1.0 mmol), anthranilamide (2-aminobenzamide) (1 mmol) and hercynite@sulfuric acid (9 mg) was stirred in ethanol under reflux conditions for the required time. The progress of reaction was monitored by TLC. After completion of the reaction, the mixture was cooled down to room temperature. Afterwards, the catalyst was collected using magnetic decantation and, then, washed with hot ethyl acetate and water. Finally, the pure polyhydroquinoline products were obtained through recrystallization in ethanol.

### 2.4. Spectral data

All of the synthesized organic compounds were characterized by ^1^H and ^13^C NMR spectroscopy and the copies of original spectrums are reported in the ESI.†

#### 2.4.1 Ethyl 4-(pyridin-3-yl)-2,7,7-trimethyl-5-oxo-1,4,5,6,7,8-hexayidroquinolin-3-carboxylate

Mp = 230–233 °C, ^1^H NMR (500 MHz, DMSO-d_6_): *δ* = 0.80 (s, 3H), 0.99 (s, 3H), 1.09 (t, *J* = 7 Hz, 3H), 1.94–1.99 (m, 1H), 2.14–2.18 (m, 1H), 2.25–2.31 (m, 4H), 2.29–2.43 (m, 1H), 3.93–4.0 (q, *J* = 7 Hz, 2H), 4.82 (s, 1H), 7.20–7.22 (m, 1H), 7.46–7.48 (m, 1H), 8.23–8.27 (m, 1H), 8.35–8.36 (d, *J* = 5 Hz, 1H), 9.15 (s, 1H, NH). ^13^C NMR (126 MHz, DMSO-d_6_): *δ* = 14.54, 18.75, 26.87, 29.47, 32.63, 34.55, 50.53, 59.61, 103.11, 109.70, 123.71, 135.26, 143.20, 146.35, 147.37, 149.34, 150.35, 166.95, 194.69 ppm.

#### 2.4.2 Ethyl 4-(4-dimethylamino)phenyl)-2,7,7-trimethyl-5-oxo-1,4,5,6,7,8-hexayidroquinolin-3-carboxylate

MP = 234–237 °C, ^1^H NMR (500 MHz, DMSO-d_6_): *δ* = 0.86 (s, 3H), 0.99 (s, 3H), 1.12–1.15 (t, *J* = 7.0 Hz, 3H), 1.93–1.97 (m, 1H), 2.12–2.15 (m, 1H), 2.24–2.28 (m, 4H), 2.37–2.40 (m, 1H), 2.78 (s, 6H), 3.94–4.01 (m, 2H), 4.72 (s, 1H), 6.52–6.54 (d, *J* = 10 Hz, 3H), 6.93–6.95 (d, *J* = 10 Hz, 3H), 8.93 (s, 1H), ^13^C NMR (126 MHz, DMSO-d_6_): *δ* = 14.66, 18.70, 27.05, 29.64, 32.58, 35.02, 50.80, 59.37, 104.74, 110.81, 112.48, 128.40, 136.53, 144.55, 149.12, 149.47, 167.54, 194.72 ppm.

#### 2.4.3 Ethyl 2,7,7-trimethyl-5-oxo-4-(*p*-tolyl)-1,4,5,6,7,8-hexahydroquinoline-3-carboxylate

MP = 194–195 °C, ^1^H NMR (500 MHz, DMSO) *δ*: 0.83 (s, 3H), 0.99 (s, 3H), 1.11–1.13 (t, 3H, *J* = 7 Hz), 1.93–1.96 (d, *J* = 15 Hz, 1H), 2.12–2.18 (m, 4H), 2.22–2.31 (m, 4H), 2.38–2.41 (d, *J* = 15 Hz, 1H),3.93–3.97 (q, 2H, *J* = 7 Hz), 4.80 (s, 1H), 6.95–6.96 (d, *J* = 5 Hz, 2H), 6.95–6.96 (d, *J* = 5 Hz, 2H), 7.01–7.02 (d, *J* = 5 Hz, 2H), 9.00 (s, 1H), ^13^C NMR (126 MHz, DMSO-d_6_): *δ* = 14.61, 18.72, 21.02, 26.92, 29.61, 32.58, 35.84, 39.92, 50.72, 59.44, 104.24, 110.55, 127.82, 128.74, 134.97, 145.19, 145.25, 149.80, 167.35, 194.67 ppm.

#### 2.4.4 2,7,7-Trimethyl-4-(4-nitro-phenyl)-5-oxo-1,4,4a,5,6,7,8,8a-octahydro-quinoline-3-carboxylic acid ethyl ester

Mp = 234–236 °C. ^1^H NMR (500 MHz, DMSO-d_6_): *δ* = 0.81 (s, 3H), 0.99 (s, 3H), 1.08–1.11 (t, *J* = 7 Hz, 3H), 1.96–1.98 (d, *J* = 10 Hz, 1H), 2.15–2.19 (m, 1H), 2.28–2.38 (m, 4H), 2.42 (m, 1H), 3.90–4.00 (m, 2H), 4.97 (s, 1H), 7.48–7.62 (m, 2H), 7.94–7.99 (m, 2H), 9.25 (s, 2H). ^13^C NMR (125 MHz, DMSO-d_6_): *d* = 14.44, 18.67, 26.75, 29.48, 31.61, 32.61, 36.89, 50.48, 59.67, 103.10, 109.68, 121.31, 122.49, 129.82, 134.76, 146.59, 147.82, 150.23, 150.59, 166.84, 194.74 ppm.

#### 2.4.5 4-(4-Chloro-phenyl)-2,7,7-trimethyl-5-oxo-1,4,4a,5,6,7,8,8aoctahydro-quinoline-3-carboxylic acid ethyl ester

Mp = 234–245 °C. ^1^H NMR (500 MHz, DMSO-d_6_): *δ* = 0.81 (s, 3H), 0.98 (s, 3H), 1.10 (t, *J* = 7. Hz, 3H), 1.96 (d, *J* = 20 Hz, 1H), 2.15 (d, *J* = 20 Hz, 1H), 2.25–2.28 (m, 4H), 2.38–41 (m, 1H), 3.92–3.99 (q, *J* = 7. Hz, 2H), 4.84 (s, 1H), 7.14–7.16 (d, *J* = 10 Hz, 2H), 7.21–7.23 (d, *J* = 10 Hz, 2H) 9.09 (s, 1H). ^13^C NMR (126 MHz, DMSO-d_6_): *δ* = 14.56, 18.75, 26.88, 29.53, 32.57, 36.06, 39.88, 50.63, 59.54, 103.58, 110.13, 128.12, 129.78, 130.65, 145.86, 147.02, 150.05, 167.10, 194.68 ppm.

#### 2.4.6 Ethyl 1,4,5,6,7,8-hexahydro-4-(4-isopropylphenyl)-2,7,7-trimethyl-5-oxoquinoline-3-carboxylate

MP 182–184 °C, ^1^H NMR (500 MHz, DMSO-d_6_): *δ* = 0.86 (s, 3H), 0.98 (s, 3H), 1.10–1.19 (m, 9H), 1.95–1.99 (m, 1H), 2.12–2.16 (m, 1H), 2.24 (s, 3H), 2.28 (m, 1H), 2.37 (m, 1H), 2.71–2.79 (se, *J* = 5 Hz, 1H), 3.94–3.98 (q, *J* = 7 Hz, 2H), 4.82 (s, 1H), 6.98–7.06 (m, 4H), 9.05 (s, 1H); ^13^C NMR (126 MHz, DMSO-d_6_) *δ* = 14.58, 18.71, 24.24, 24.39, 27.09, 29.51, 32.58, 33.40, 35.74, 50.71, 59.51, 104.39, 110.42, 126.06, 127.77, 145.12, 145.56, 146.01, 150.16, 167.43, 194.91 ppm.

#### 2.4.7 Ethyl-2,7,7-trimethyl-5-oxo-4-(pyridin-4-yl)-1,4,5,6,7,8-hexahydroquinoline-3-carboxylate

M.p: 218–220 °C; ^1^H NMR (500 MHz, DMSO-d_6_): *δ* = 0.80 (s, 3H), 0.98 (s, 3H), 1.07–1.10 (t, *J* = 7 Hz, 3H), 1.98 (m, 1H), 2.16 (m, 1H), 2.27–2.34 (m, 4H), 2.40 (m, 1H), 3.94–3.98 (q, *J* = 7 Hz, 2H), 4.85 (s, 1H), 7.12 (d, *J* = 5 Hz, 2H), 8.37 (s, 2H), 9.19 (s, 1H); ^13^C NMR (126 MHz, DMSO-d_6_) *δ* = 194.73, 166.90, 155.82, 150.66, 149.68, 146.70, 123.27, 109.21, 102.42, 59.66, 50.53, 39.84, 36.25, 32.56, 29.46, 26.83, 18.75, 14.52.

#### 2.4.8 Ethyl 2,7,7-trimethyl-5-oxo-4-phenyl-1,4,5,6,7,8-hexahydroquinolin-3-carboxylate

M.p: 201–203 °C; ^1^H NMR (500 MHz, DMSO-d_6_): *δ* = 0.83 (s, 3H), 0.99 (s, 3H), 1.09–1.12 (t, *J* = 7.0 Hz, 3H), 1.96 (d, 1H), 2.15 (d, 1H), 2.23–2.31 (m, 4H), 2.38–2.42 (m, 1H), 3.93–3.98 (q, *J* = 7 Hz, 2H), 4.84 (s, 1H), 7.3–7.06 (m, 1H), 7.11–7.19 (m, 4H), 9.04 (s, 1H); ^13^C NMR (126 MHz, DMSO-d_6_) *δ* = 194.70, 167.30, 149.95, 148.10, 145.43, 128.16, 127.91, 126.12, 110.43, 104.07, 59.47, 50.70, 39.92, 36.31, 32.58, 29.58, 26.90, 18.73, 14.59.

#### 2.4.9 Ethyl 4-(4-methoxyphenyl)-2,7,7-trimethyl-5-oxo-1,4,5,6,7,8- hexahydroquinoline-3-carboxylate

M.p: 257–260 °C; ^1^H NMR (500 MHz, DMSO-d_6_): *δ* = 0.83 (s, 3H), 0.99 (s, 3H), 1.08–1.11 (t, *J* = 7.0 Hz, 3H), 1.86–1.90 (d, 1H), 2.08–2.14 (d, 1H), 2.17–2.25 (m, 4H), 2.36–2.39 (m, 1H), 3.68 (s, 3H), 3.87–3.96 (m, 2H), 5.03 (s, 1H), 6.72–6.75 (m, 1H), 6.79–6.82 (m, 1H), 7.01–7.04 (m, 1H), 7.08–7.09 (m, 1H), 8.92 (s, 1H); ^13^C NMR (126 MHz, DMSO-d_6_) *δ* = 194.27, 167.75, 157.60, 150.42, 144.57, 135.42, 130.96, 127.39, 119.93, 111.45, 109.13, 103.39, 59.21, 55.62, 50.85, 33.27, 32.43, 29.77, 26.64, 18.49, 14.54.

#### 2.4.10 Ethyl 4-(4-bromophenyl)-2,7,7-trimethyl-5-oxo-1,4,5,6,7,8-hexahydroquinoline-3 carboxylate

M.p: 249–252 °C; ^1^H NMR (500 MHz, DMSO-d_6_): *δ* = 0.85 (s, 3H), 1.0 (s, 3H), 1.13 (b, s, 3H), 1.95–1.98 (d, 1H), 2.19 (s, 1H), 2.28 (s, 3H), 2.39 (m, 1H), 3.97 (b, s, 2H), 4.82 (s, 1H), 6.97–7.04 (m, 4H), 9.01 (s, 1H); ^13^C NMR (126 MHz, DMSO-d_6_) *δ* = 14.61, 18.73, 21.02, 26.93, 29.62, 32.58, 35.87, 50.74, 59.44, 104.27, 110.58, 127.84, 128.74, 134.97, 145.20, 145.27, 149.82, 167.35, 194.68 ppm.

#### 2.4.11 Ethyl 2,7,7-trimethyl-5-oxo-4-(4-hydroxy)-1,4,5,6,7,8-hexahydroquinoline-3-carboxylate

M.p: 225–228 °C; ^1^H NMR (500 MHz, DMSO-d_6_): *δ* = 0.85 (s, 3H), 1.00 (s, 3H), 1.12–1.15 (t, *J* = 7 Hz, 3H), 1.95–1.98 (d, *J* = 15 Hz, 1H), 2.13–2.16 (d, *J* = 15 Hz, 1H), 2.26–2.28 (m, 4H), 2.38–2.41 (m, 1H), 2.40–2.44 (d, *J* = 16 Hz, 1H), 3.96–3.98 (q, *J* = 7 Hz, 2H), 4.74 (s, 1H), 6.54–6.56 (m, 2H), 6.92–6.93 (m, 2H), 8.92 (s, 1H), 9.02 (s, 1H); ^13^C NMR (126 MHz, DMSO-d_6_) *δ* = 14.64, 18.71, 26.94, 29.63, 32.59, 35.25, 39.94 50.78, 59.41,104.58, 110.80, 114.90, 128.79, 138.88, 144.84, 149.58, 155.70, 167.48, 194.74 ppm.

#### 2.4.12 Dimethyl 4-(3-ethoxy-4-hydroxyphenyl)-2,6-dimethyl-1,4-dihydropyridine-3,5-dicarboxylate

Mp: 197–199 °C; ^1^H NMR (500 MHz, DMSO-d_6_): *δ* = 0.87 (s, 3H), 1.00 (s, 3H), 1.11–1.16 (t, 3H, *J* = 7 Hz), 1.27–1.30 (t, 3H, *J* = 7 Hz), 1.96–1.99 (d, 1H), 2.14–2.18 (d, 1H), 2.22–2.28 (m, 4H), 2.39–2.42 (d, 1H), 3.85–3.93 (m, 2H), 3.97–4.01 (q, *J* = 7 Hz, 2H), 4.73 (s, 1H), 6.50 (d, 1H, *J* = 7 Hz), 6.58 (d, 1H, *J* = 7 Hz), 6.64 (s, 1H); ^13^C NMR (126 MHz, DMSO-d_6_) *δ* = 14.69, 15.25, 18.70, 26.84, 29.69, 32.57, 35.44, 50.78, 59.44, 64.24, 104.49, 110.68, 114.01, 115.47, 120.12, 139.42, 144.86, 145.35, 146.24, 149.67, 167.51, 194.82 ppm.

#### 2.4.13 Ethyl 4-(3,4-dimethoxyphenyl)-2,7,7-trimethyl-5-oxo-1,4,5,6,7,8-hexahydroquinoline-3-carboxylate

MP: 199–201 °C; ^1^H NMR (500 MHz, DMSO-d_6_): *δ* = 0.88 (s, 3H), 1.01 (s, 3H, CH_3_), 1.14–1.17 (t, *J* = 7. Hz, 3H), 1.97–2.00 (d, *J* = 5 Hz, 1H), 2.15–2.19 (d, *J* = 15 Hz, 1H), 2.27–2.30 (m, 4H), 2.40–2.44 (m, 1H), 3.66 (s, 3H), 3.66 (s, 3H), 3.97–4.02 (q, *J* = 7 Hz, 2H), 4.79 (s, 1H), 6.61–6.63 (t, *J* = 5 Hz, 1H), 6.74–6.77 (m, 2H), 9.02 (s, 1H). ^13^C NMR (126 MHz, DMSO-d_6_) *δ* = 14.71, 18.72, 26.89, 29.68, 32.58, 35.60, 50.75, 55.76, 55.87, 59.48, 104.33, 110.52, 111.94, 112.18, 119.70, 140.96, 145.07, 147.43, 148.45, 149.87, 167.44, 194.83 ppm.

#### 2.4.14 1,4-bis(3-ethoxylcarbonyl-1,4,5,6,7,8-hexahydro-5-oxo-2,7,7-trimethylquinoline-4-yl)benzene

MP: 305–307 °C; ^1^H NMR (500 MHz, DMSO-d_6_): *δ* = 0.84 (s, 6H), 0.98 (s, 6H), 1.07–1.14 (t, *J* = 7 Hz, 6H), 1.92–2.14 (m, 4H), 2.26 (s, 6H), 2.30–240 (m, 4H), 3.96–3.98 (q, *H* = 7 Hz, 4H), 4.81 (s, 2H), 6.90–6.97 (m, 4H), 9.00 (s, 2H); ^13^C NMR (126 MHz, DMSO-d_6_) *δ* = 14.49, 14.59, 18.71, 18.79, 26.61, 27.25, 29.36, 29.68, 32.60, 32.63, 35.28, 35.81, 50.73, 59.36, 59.47, 103.86, 104.27, 110.34, 110.57, 127.11, 127.28, 145.15, 145.45, 145.53, 150.09, 167.33, 167.40, 194.68, 194.82 ppm.

#### 2.4.15 2-Phenyl-2,3-dihydroquinazolin-4(1*H*)-one

M.P: 166–168 °C; ^1^H NMR (500 MHz, DMSO-d_6_): *δ* = 5.76 (s, 1H); 6.68 (m, 1H), 6.76 (m, 1H), 7.10 (m, 1H), 7.26 (m, 1H), 7.33 (m, 1H), 7.40 (m, 1H), 7.50–7.64 (m, 3H), 8.19 (m, 1H), 8.29 (m, 1H); ^13^C NMR (126 MHz, DMSO-d_6_) *δ* = 46.50, 67.05, 114.72, 117.58, 127.33, 127.82, 128.78, 128.91, 129.08, 133.78, 135.09, 142.09, 148.34, 164.08 ppm.

#### 2.4.16 2-(Pyridin-4-yl)-2,3-dihydroquinazolin-4(1*H*)-one

M.P: 218–220 °C; ^1^H NMR (500 MHz, DMSO-d_6_): *δ* = 5.86 (s, 1H); 6.69–6.77 (m, 2H), 7.17 (s, 1H), 7.25–7.29 (m, 1H), 7.41–7.44 (m, 1H), 7.62–7.64 (m, 1H), 7.85–7.91 (m, 1H), 8.38 (s, 1H), 8.55–8.56 (m, 1H), 8.67–8.68 (m, 1H); ^13^C NMR (126 MHz, DMSO-d_6_) *δ* = 65.15, 115.03, 115.50, 117.98, 124.01, 127.87, 133.94, 135.13, 137.29, 148.17, 148.84, 150.13, 164.05 ppm.

#### 2.4.17 2-(4-chlorophenyl)-2,3-dihydroquinazolin-4(1*H*)-one

M.P: 191–193 °C; ^1^H NMR (500 MHz, DMSO-d_6_): *δ* = 5.77 (s, 1H), 6.78 (m, 1H), 6.76 (m, 1H), 7.14 (s, 1H), 7.23–7.27 (m, 1H), 7.41–7.55 (m, 4H), 7.60–7.64 (m, 1H), 8.33 (s, 1H); ^13^C NMR (126 MHz, DMSO-d_6_) *δ* = 66.22, 114.93, 115.41, 117.74, 127.83, 128.77, 129.22, 130.10, 133.86, 141.15, 148.11, 163.96 ppm.

#### 2.4.18 2-(4-Bromophenyl)-2,3-dihydroquinazolin-4(1*H*)-one

M.P: 200–202 °C; ^1^H NMR (500 MHz, DMSO-d_6_) *δ* = 6.34 (t, *J* = 2.2 Hz, 1H), 6.72 (t, *J* = 7.5 Hz, 1H), 6.78 (d, *J* = 8.1 Hz, 1H), 7.01 (s, 1H), 7.26 (td, *J* = 7.8, 1.7 Hz, 1H), 7.69–7.56 (m, 2H), 7.82–7.75 (m, 1H), 7.86 (dd, *J* = 7.9, 1.6 Hz, 1H), 8.07 (dd, *J* = 8.2, 1.3 Hz, 1H), 8.22 (s, 1H); ^13^C NMR (126 MHz, DMSO-d_6_) *δ* = 62.64, 114.98, 115.37, 118.14, 125.17, 127.78, 129.40, 130.35, 134.02, 134.37, 136.38, 147.58, 148.12, 163.83 ppm.

#### 2.4.19 2-(4-Nitrophenyl)-2,3-dihydroquinazolin-4(1*H*)-one

M.P: 195–197 °C; ^1^H NMR (500 MHz, DMSO-d_6_) *δ* = 5.92 (d, *J* = 2.6 Hz, 1H), 6.68 (q, *J* = 6.3 Hz, 1H), 6.78 (d, *J* = 8.1 Hz, 1H), 7.30–7.23 (m, 1H), 7.32 (s, 1H), 7.62 (d, *J* = 7.7 Hz, 1H), 7.75 (d, *J* = 8.4 Hz, 2H), 8.28–8.17 (m, 2H), 8.52 (s, 1H); ^13^C NMR (126 MHz, DMSO-d_6_) *δ* = 65.77, 115.02, 115.37, 117.94, 124.04, 127.88, 128.49, 134.03, 147.70, 147.89, 149.78, 163.77 ppm.

#### 2.4.20 2-(2-Hydoxyphenyl)-2,3-dihydroquinazolin-4(1*H*)-one

M.P: 222–224 °C; ^1^H NMR (500 MHz, DMSO-d_6_) *δ* = 7.04–6.87 (m, 3H), 7.42 (d, *J* = 7.8 Hz, 1H), 7.52 (t, *J* = 7.5 Hz, 1H), 7.72 (d, *J* = 8.2 Hz, 1H), 7.82 (t, *J* = 7.7 Hz, 1H), 8.18 (dd, *J* = 40.3, 8.0 Hz, 3H), 12.44 (s, 1H), 13.78 (s, 1H); ^13^C NMR (126 MHz, DMSO-d_6_) *δ* = 114.13, 118.34, 119.24, 121.17, 126.48, 127.35, 128.13, 134.14, 135.41, 146.53, 154.19, 160.57, 161.86 ppm.

#### 2.4.21 2,2′-(1,4-phenylene)bis(2,3-dihydroquinazolin-4(1*H*)-one)

M.P: 242–245 °C; ^1^H NMR (500 MHz, DMSO-d_6_) *δ* = 6.80–6.60 (m, 2H), 7.26 (dq, *J* = 24.9, 8.9 Hz, 3H), 7.58–7.46 (m, 3H), 7.87–7.69 (m, 4H), 8.25–8.02 (m, 3H), 8.36 (d, *J* = 8.6 Hz, 1H), 9.01–8.60 (m, 1H), 10.16–9.85 (m, 1H), ^13^C NMR (126 MHz, DMSO-d_6_) *δ* = 56.54, 121.57, 121.63, 126.37, 127.15, 129.00, 135.14, 148.77, 163.07 ppm.

#### 2.4.22 2-(4-Hydroxy)-2,3-dihydroquinazolin-4(1*H*)-one

M.P: 272–275 °C; ^1^H NMR (500 MHz, DMSO-d_6_) *δ* = 6.91 (d, *J* = 8.3 Hz, 3H), 7.44 (t, *J* = 7.5 Hz, 1H), 7.67 (d, *J* = 8.2 Hz, 1H), 7.77 (t, *J* = 7.7 Hz, 1H), 8.11 (dd, *J* = 12.0, 8.1 Hz, 4H), 10.16 (s, 1H), 12.45–12.07 (m, 1H); ^13^C NMR (126 MHz, DMSO-d_6_) *δ* = 113.36, 115.83, 121.05, 123.70, 126.28, 126.33, 127.65, 130.06, 134.90, 149.52, 152.60, 161.03, 162.81 ppm.

#### 2.4.23 2-(4-Methoxyphenyl)-2,3-dihydroquinazolin-4(1*H*)-one

M.P: 183–185 °C; ^1^H NMR (400 MHz, DMSO-d_6_): *δ* = 3.75 (s, 3H), 5.71 (t, *J* = 1.5 Hz, 1H), 6.68 (td, *J* = 7.4, 1.1 Hz, 1H), 6.74 (dd, *J* = 8.2, 1.0 Hz, 1H), 6.97–6.89 (m, 2H), 7.02 (s, 1H), 7.30–7.20 (m, 1H), 7.46–7.38 (m, 2H), 7.61 (dd, *J* = 7.7, 1.7 Hz, 1H), 8.20 (d, *J* = 2.2 Hz, 1H).

#### 2.4.24 2-(4-Tolyl)-2,3-dihydroquinazolin-4(1*H*)-one

M.P: 219–220 °C; ^1^H NMR (400 MHz, DMSO-d_6_): *δ* = 2.30 (s, 3H), 5.71 (t, *J* = 1.8 Hz, 1H), 6.67 (td, *J* = 7.5, 1.1 Hz, 1H), 6.74 (dd, *J* = 8.1, 1.1 Hz, 1H), 7.06 (s, 1H), 7.28–7.16 (m, 3H), 7.41–7.34 (m, 2H), 7.61 (dd, *J* = 7.8, 1.6 Hz, 1H), 8.24 (t, *J* = 1.9 Hz, 1H) ppm.

#### 2.4.25 2-(Pyridin-3-yl)-2,3-dihydroquinazolin-4(1*H*)-one

M.P: 218–220 °C; ^1^H NMR (400 MHz, DMSO-d_6_): *δ* = 5.86 (s, 1H), 6.79–6.66 (m, 2H), 7.17 (s, 1H), 7.30–7.19 (m, 1H), 7.43 (ddd, *J* = 7.7, 5.0, 2.2 Hz, 1H), 7.63 (d, *J* = 7.8 Hz, 1H), 7.93–7.83 (m, 1H), 8.38 (s, 1H), 8.55 (dd, *J* = 4.7, 2.1 Hz, 1H), 8.68 (d, *J* = 2.5 Hz, 1H); ^13^C NMR (126 MHz, DMSO-d_6_) *δ* = 65.15, 115.03, 115.50, 117.98, 124.01, 127.87, 133.94, 135.13, 137.29, 148.17, 148.84, 150.13, 164.05 ppm.

#### 2.4.26 2-(3,4-dimethoxyphenyl)-2,3-dihydroquinazolin-4(1*H*)-one

M.P: 210–212 °C; ^1^H NMR (400 MHz, DMSO-d_6_): *δ* = 3.01 (d, *J* = 1.9 Hz, 6H), 6.71–6.56 (m, 1H), 6.82–6.75 (m, 2H), 7.15–6.86 (m, 2H), 7.42 (t, *J* = 7.6 Hz, 1H), 7.67–7.50 (m, 2H), 7.77 (t, *J* = 7.6 Hz, 1H), 8.21–8.04 (m, 3H) ppm.

#### 2.4.27 2-(4-(dimethylamino)phenyl)-2,3-dihydroquinazolin-4(1*H*)-one

M.P: 206–208 °C; ^1^H NMR (400 MHz, DMSO-d_6_): *δ* = 3.01 (d, *J* = 1.9 Hz, 6H), 6.71–6.56 (m, 1H), 6.82–6.75 (m, 2H), 7.15–6.86 (m, 2H), 7.42 (t, *J* = 7.6 Hz, 1H), 7.67–7.50 (m, 2H), 7.77 (t, *J* = 7.6 Hz, 1H), 8.21–8.04 (m, 3H); ^13^C NMR (126 MHz, DMSO-d_6_) *δ* = 40.79, 111.69, 113.28, 119.21, 120.81, 125.86, 126.27, 129.20, 129.34, 129.85, 134.87, 152.75 ppm.

## 3. Results and discussion

### 3.1. Catalyst preparation

**Scheme 1:**
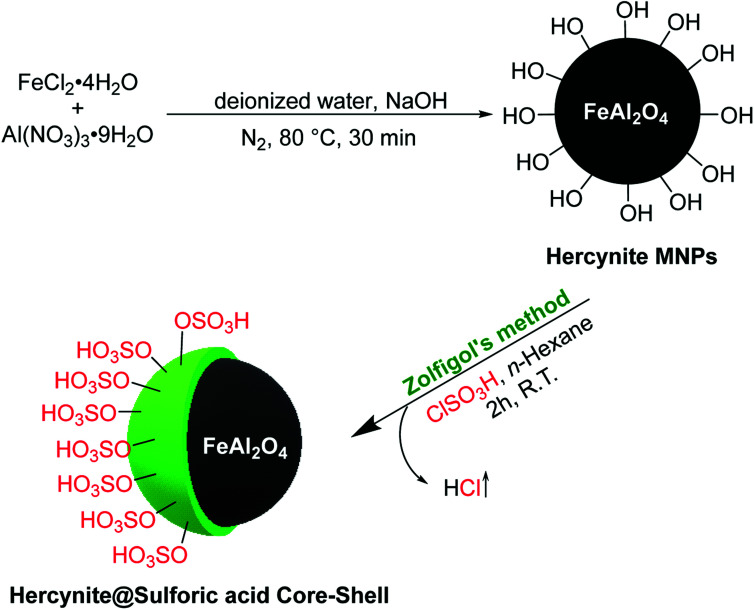
The synthesis of hercynite@sulfuric acid nanoparticles.

The novel sulfuric acid-modified Hercynite MNPs were readily synthesized according to the route depicted in Scheme 1. First, the coprecipitation reaction of commercially available FeCl_2_·4H_2_O and Al(NO_3_)_2_·9H_2_O in deionized water, at 80 °C for 30 min and in the presence of NaOH as base, provided the hercynite MNPs (FeAl_2_O_4_). The subsequent reaction of hercynite MNPs with chlorosulfuric acid in *n*-hexane, which is associated with the removal of HCl gas from the reaction vessel, immediately delivered the sulfuric acid-modified hercynite MNPs (hercynite@sulfuric acid).

### 3.2. Catalyst characterizations

The as-prepared hercynite@sulfuric acid core–shell was then fully characterized using different physico-chemical techniques; including, FT-IR, XRD, EDX, X-ray-mapping, SEM and VSM analysis.

**Fig. 1:**
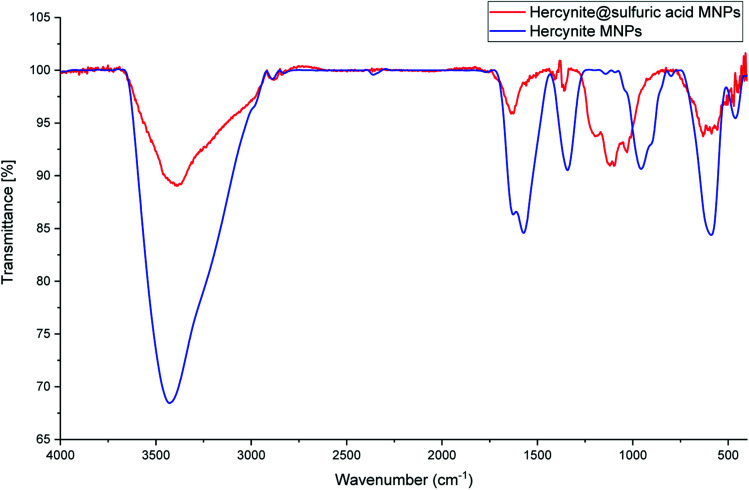
The FT-IR spectrums of (blue curve ) hercynite and (red curve ) hercynite@sulfuric acid MNPs.

The FT-IR spectra of the hercynite (a) and hercynite@sulfuric acid (b) are shown in Fig. 1. Both of the FT-IR patterns are completely consistent with the previous analyses of hercynite (FeAl_2_O_4_) MNPs.^45^ As it can be seen on both of the FT-IR spectrums, the presence of vibration bands at 425, 591 and 3409 cm^−1^ are due to Fe–O and OH bonds, respectively. In addition, the weak adsorption peaks around 827 and 855 cm^−1^ are related to Al–O bond. Moreover, the bands at 1339, 1383 and1650 cm^−1^ correspond to the nitrate impurity vibrations.^45^ Finally, the presence of 994–1237 cm^−1^ and 3000–3400 cm^−1^ bands in FT-IR spectra of hercynite@sulfuric acid (Fig. 1b) confirms the successful functionalization of hercynite with the SO_3_H groups.^46^

**Fig. 2:**
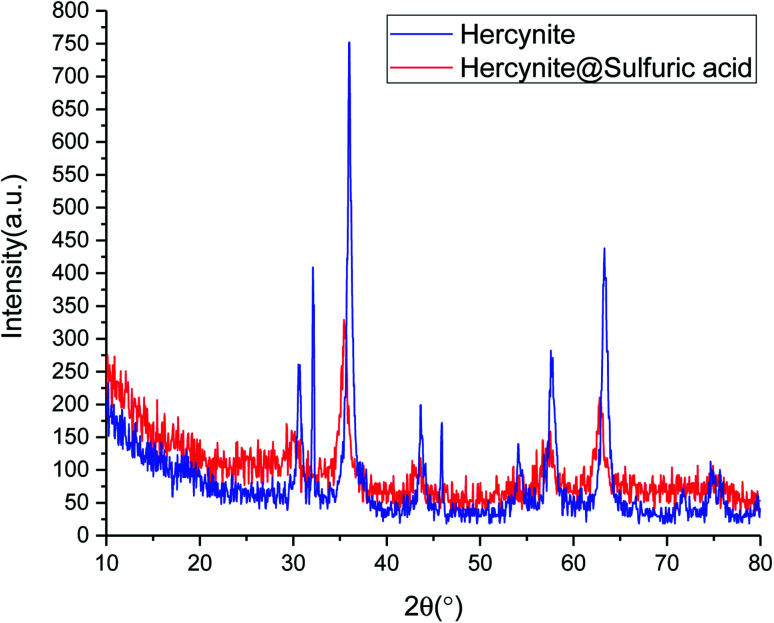
The XRD patterns of hercynite and hercynite@sulfuric acid MNPs.

Fig. 2 shows normal angle powder X-ray diffraction (P-XRD) patterns of hercynite (blue curve) and hercynite@Sulfuric acid (red curve). The P-XRD pattern of hercynite exhibits several peaks, which are in good agreement with XRD pattern of the spinel-type FeAl_2_O_4_ MNPs.^45^ The XRD pattern of hercynite@sulfuric acid was similar to that of hercynite, indicating that hercynite@sulfuric acid also contains the octahedral structures. However, after anchoring the sulfuric acid cites, the peak intensity of hercynite@sulfuric acid significantly decreased and the background became noisy. Conversely, the three high order (0 3 1), (1 1 0) and (4 2 2) diffraction peaks disappeared, indicating the peak reflections originating from the core magnetite and also the noisy background coming from the amorphous dried SO_3_H shells. Moreover, it also confirmed that the sulfuric acid groups were mainly anchored on the surface of the hercynite MNPs.^47^ These results indicated that the textural characteristics of hercynite were preserved – during the supported SO_3_H catalyst preparation and the crystalline phase – and the structural properties remained accessible.^44^

**Fig. 3:**
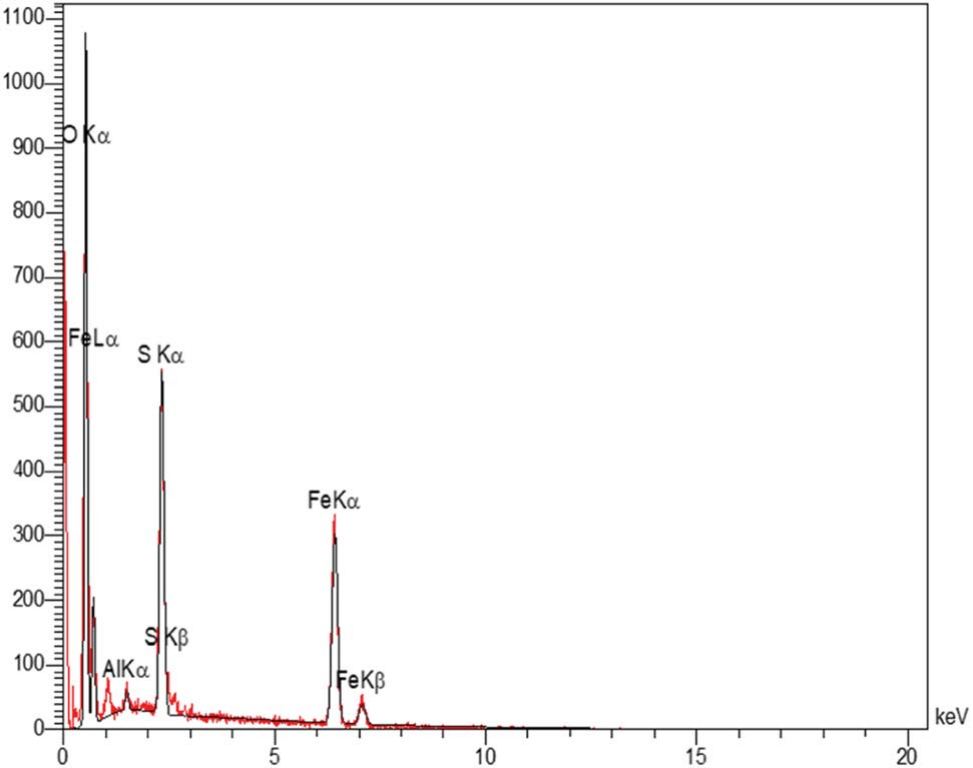
EDX analysis of hercynite@sulfuric acid MNPs.

In order to have an idea of chemical composition of the nanocomposite, energy dispersive X-ray (EDX) analysis was carried out, as the profile is shown in Fig. 3 representing the Fe and Al as metallic components. The presence of sulfur and oxygen confirmed the successful fabrication of sulfuric acid shell over the hercynite surface. In addition, there are no other elements, showing the high purity of the sample. Accordingly, the sulfur presence was witnessed; but, we did not observe any amount of Cl, indicating that it was on the catalyst surface that the covalent adsorption of SO_3_H groups has successfully occurred. Besides, the Cl was removed as HCl gas from the reaction vessel, immediately.

**Fig. 4:**
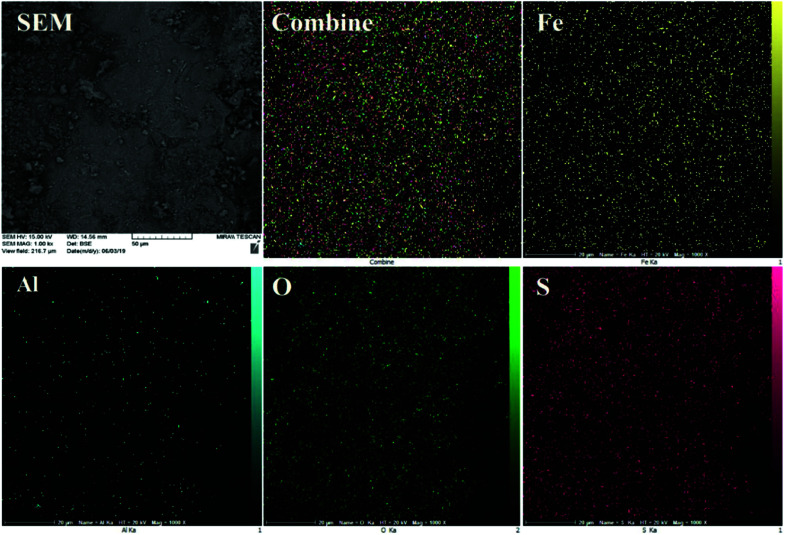
EDX mapping images of hercynite@sulfuric acid MNPs.

The EDS results were further justified by FE-SEM elemental mapping analysis (Fig. 4). The compositional map reveals that the Fe, Al, O and S species exist with excellent homogeneously dispersion throughout the matrix surface. In this sense, fine distribution of active sulfuric acid species on the hercynite surface definitely has a significant impact on the catalytic performance because of the good availability of the sulfonated Brønsted acid catalytic cites.

**Fig. 5:**
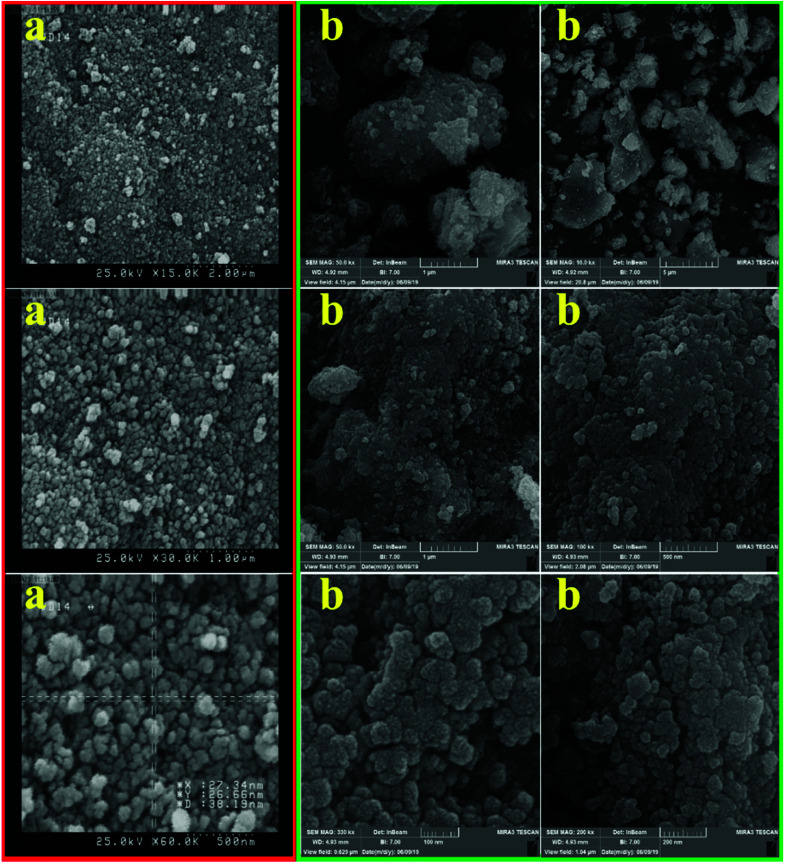
SEM images of (a) hercynite and (b) hercynite@sulfuric acid MNPs.

The inherent microstructural features, morphology, texture and shape of the as-synthesized hercynite (a) and hercynite@sulfuric acid MNPs (b) were determined using scanning electron microscopy (SEM) analysis. Fig. 5a, representing the SEM image, depicts the globular morphology of the hercynite NPs having a mean diameter of 27–38 nm. Moreover, the hercynite@sulfuric acid MNPs (Fig. 5b) which consist of a set of regular particles like its hercynite parent (Fig. 5a) are in an approximately spherical shape and uniform nanometer size for most particles and have a specific heterogeneity of the surface. A thin uniform and continuous distribution of sulfuric acid shell over the hercynite core ferrite towards the surface functionalization can be anticipated from the appearances. These type particles are good candidates for catalystic processes. However, the presence of SO_3_H functional groups cannot be separately detected from the images. Owing to manual sampling, the particles were somewhat aggregated, as can be seen from the image.

**Fig. 6:**
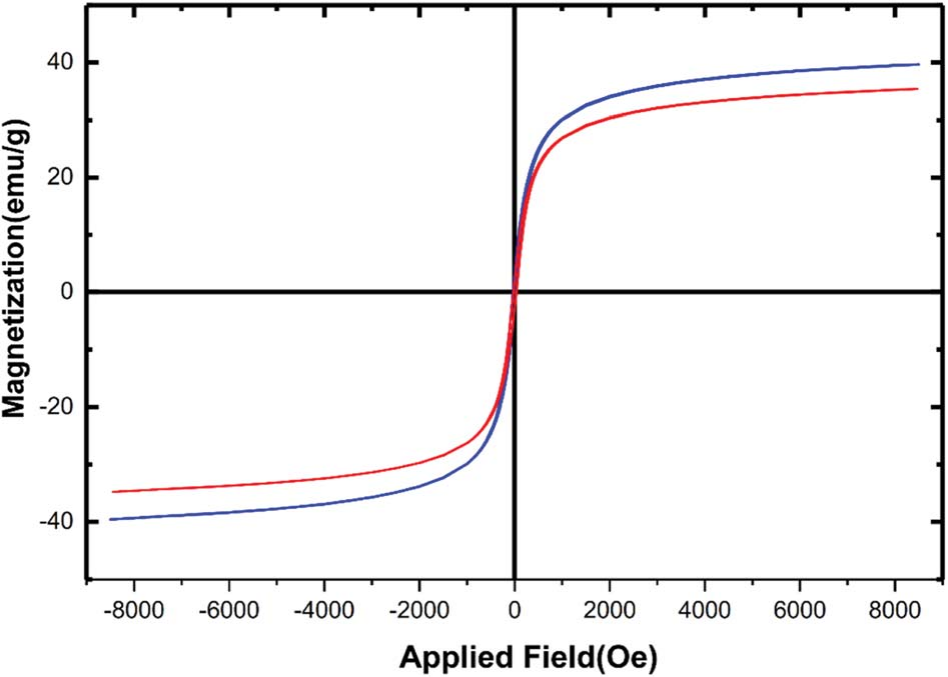
VSM curves of hercynite (blue) and hercynite@sulfuric acid MNPs (red).

Due to the iron and aluminium core-based materials, analysis of magnetism using vibrating sample magnetometer (VSM) technique seems to be an obvious measure. Fig. 6 reveals the magnetic hysteresis curves of the hercynite@sulfuric acid (red curve) and its bare hercynite synthon (blue curve). Against a variable external magnetic field, both of the materials show significant magnetic properties at room temperature and the nature of curves evidently reveals their paramagnetic character. The saturation magnetization values (Ms) of the hercynite and hercynite@sulfuric acid materials were found to be ∼40 and ∼36 emu g^−1^, respectively. The decrease in magnetism in the modified material can be predicted from the incorporation of nonmagnetic sulfuric acid shell over the magnetic hercynite core. This result confirms the successful chemical adsorption of SO_3_H shell *via* chemical bonding on the surface of magnetic core. Besides, this can be a sufficient reason to confirm the successful immobilization of sulfuric acid functional groups of hercynite surface. Nevertheless, the hercynite@sulfuric acid nanocatalyst exhibits super magnetic properties and high magnetic values, allowing it to be easily separated from the mixture with a simple external magnet.

### 3.3. Catalytic studies

Just after the successful synthesis and detailed microstructural study and analysis of structure and content of hercynite@sulfuric MNPs by rigorous instrumentation, we headed towards the catalytic exploration of these MNPs in multicomponent synthesis of a wide variety of the functionalized six-membered N-containing heterocyclic systems including the Hantzsch synthesis of polyhydroquinolines and the synthesis of 2,3-dihydroquinazolin-4(1*H*)-ones.

**Scheme 2:**
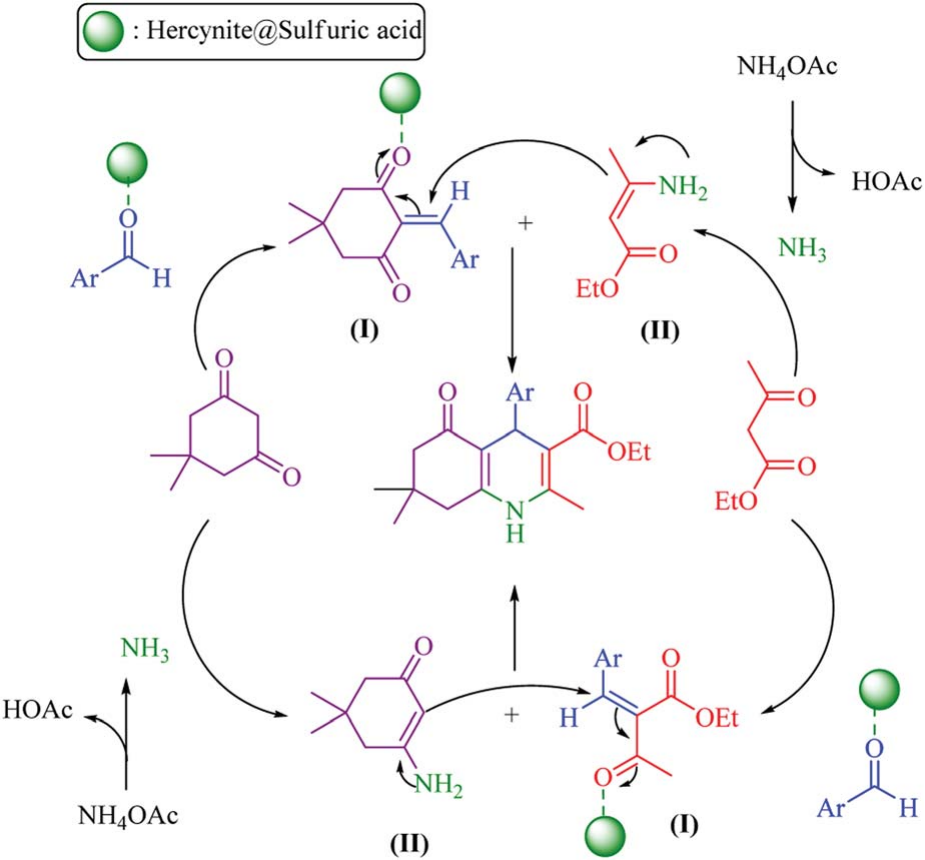
Proposed mechanism for the synthesis of polyhydroquinolines in the presence of hercynite@sulfuric acid MNPs.

Firstly, the Hantzsch synthesis of polyhydroquinolines was investigated. Subsequently, in an attempt to recognize the ideal catalytic conditions which can be regarded as a competent model for an efficient condensation of 4-chlorobenzaldehyde with dimedone, ethyl acetoacetate and ammonium acetate were chosen as the model substrates and, then, an array of investigations was carried out using various parameters like catalyst loading, solvent and temperature (Table 1). The initial optimization was started with the screening of various solvents over the as-prepared nanocatalyst at their respective refluxing temperatures which generated low to moderate yields. Evidently, solvent-free condition was found to be perfect to start the optimization of the desired transformation. Thus, we decided to continue with that for further experiments. At the first step, the effect of the catalyst amount was optimized. Afterwards, the results, conducted in presence of 7 mg of the catalyst, demonstrated that the amount of the catalyst would play a significant role as the yield of polyhydroquinoline product increases by increasing the catalyst loading. Notably, the reaction failed in the absence of any catalyst (Table 1, entry 1). At higher catalyst load, there was no further improvement in the reaction (Table 1, entry 16). In addition, the catalytic effect of hercynite on the reaction was examined; but, it did not show an interesting effect on the reaction yield in comparison to hercynite@sulfuric acid MNPs at the same conditions, which – according to the proposed mechanism – shows that the presence of SO_3_H functional groups in the catalyst plays an important role in promoting the reactions (see Scheme 2). Subsequently, the effect of different solvents was re-examined. The catalyst was found out to be efficient in solvent-free conditions. We also screened the reaction in different solvents like ethanol, methanol and water at reflux conditions, as we did not observe satisfactory yields of the product. It was found that the catalytic activity of hercynite@sulfuric acid MNPs was strongly inhibited by solvents especially when the water used as reaction medium. The results show that the catalytic activity was decreased as the amount of water present in the medium increased going from water to ethanol and methanol. The deactivating effect of water also manifested itself by changes in the activation energy. Moreover, the decreased activity of the catalytic protons is suggested to be caused by preferential solvation of them by water. Also, the solvents limiting the ceiling temperature of reaction. Finally, the effect of temperature was investigated. However, the reaction was slow at ambient temperature, at lower temperature conditions, it was not much successful and, accordingly, we continued the reactions at 100 °C. Therefore, the best results for the Hantzsch synthesis of polyhydroquinolines were obtained by heating the reaction over 7 mg of hercynite@sulfuric acid at 100 °C under solvent free conditions.

**Table 1:** Optimization of the reaction conditions for the Hantzsch condensation of 4-chlorobenzaldehyde and dimedone, ethyl acetoacetate and ammonium acetate as the model reaction for the synthesis of polyhydroquinolines

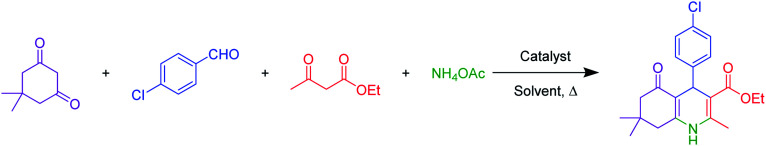						
Entry	Catalyst	Amount catalyst (mg)	Solvent	Temperature (°C)	Time (min)	Yield^a^^,^^b^ (%)
1	—	—	Solvent-free	100	4 h	Trace
2	Hercynite	7	Solvent-free	100	20	76
3	Hercynite@sulfuric acid	1	Solvent-free	100	20	29
4	Hercynite@sulfuric acid	3	Solvent-free	100	20	67
5	Hercynite@sulfuric acid	5	Solvent-free	100	20	87
6	Hercynite@sulfuric acid	6	Solvent-free	100	20	91
7	Hercynite@sulfuric acid	7	Solvent-free	100	20	99
8	Hercynite@sulfuric acid	8	Solvent-free	100	20	99
9	Hercynite@sulfuric acid	7	EtOH	Reflux	20	87
10	Hercynite@sulfuric acid	7	MeOH	Reflux	20	78
11	Hercynite@sulfuric acid	7	PEG-400	100	20	89
12	Hercynite@sulfuric acid	7	H_2_O	Reflux	20	63
13	Hercynite@sulfuric acid	7	Solvent-free	25	20	NR
14	Hercynite@sulfuric acid	7	Solvent-free	40	20	Trace
15	Hercynite@sulfuric acid	7	Solvent-free	80	20	74
16	Hercynite@sulfuric acid	7	Solvent-free	90	20	92

a Isolated yield.

b Reaction conditions: 4-chlorobenzaldehyde (1 mmol), dimedone (1 mmol), ethyl acetoacetate (1 mmol), ammonium acetate (1.2 mmol), catalyst (mg) and solvent (3 mL).

Subsequently, to investigate the scope and limitations of the prepared catalytic system for the Hantzsch synthesis of asymmetric polyhydroquinolines, a diverse range of aromatic aldehydes was employed as variable substrates in the reaction along with dimedone, ethyl acetoacetate and ammonium acetate. The outcomes are documented in Table 2. Aromatic aldehydes were converted to the corresponding dihydropyridines in short reaction times with 86–98% yields. The differently substituted aldehydes were found to be highly compatible under the stabilized conditions. There was no significant difference in the electron-releasing (Me, OMe) or -withdrawing effect (Cl, Br, NO_2_) in the yields. Finally, the isolated pure products were authenticated by comparing their corresponding melting points with the literature.

**Table 2:** Hantzsch synthesis of polyhydroquinoline derivatives in the presence of hercynite@sulfuric acid MNPs under solvent-free conditions at 100 °C

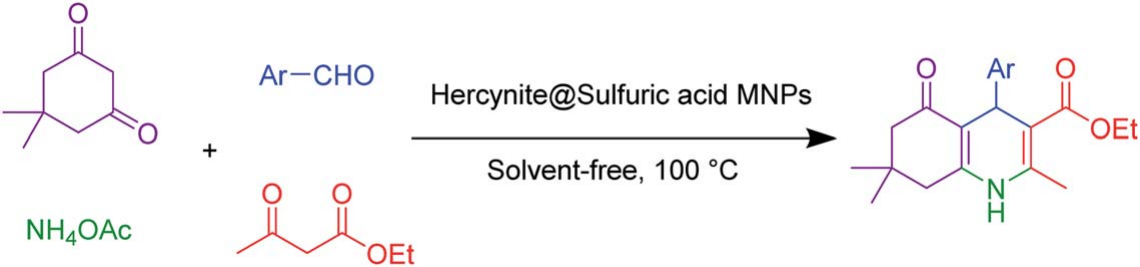						
Entry	Aryl aldehyde	Product	Time (min)	Yield^a^^,^^b^ (%)	Melting point	
					Measured	Literature
1	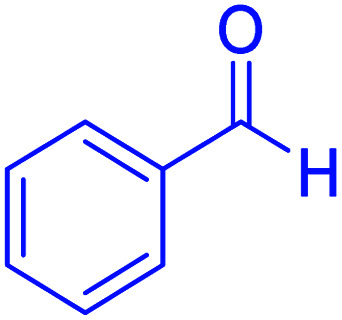	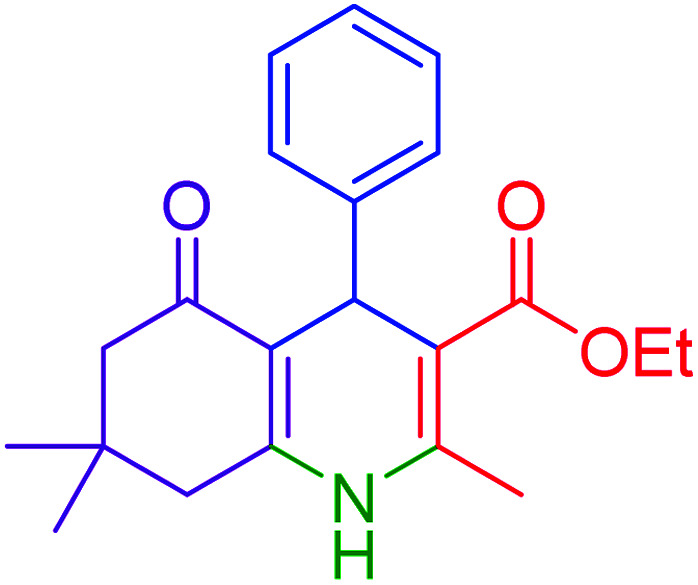	30	98	201–203	200–203 (ref. 20)
2	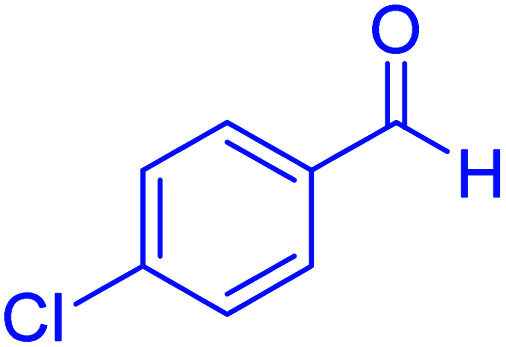	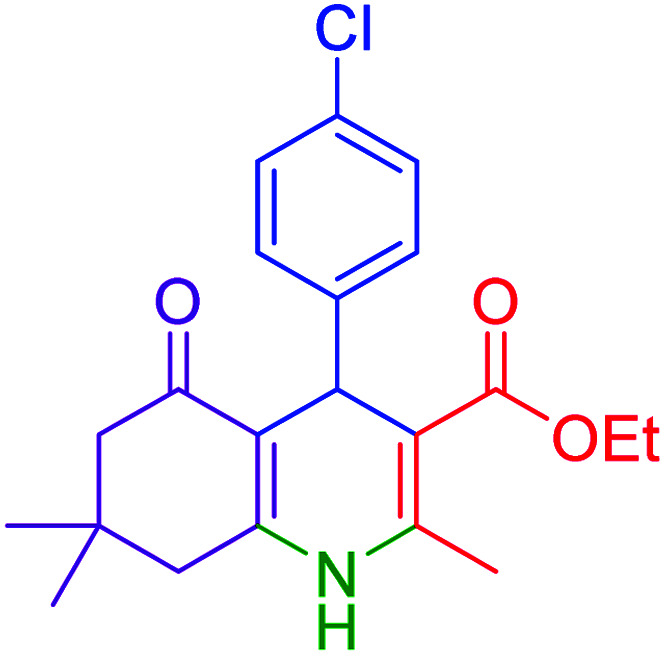	20	99	243–245	242–245 (ref. 20)
3	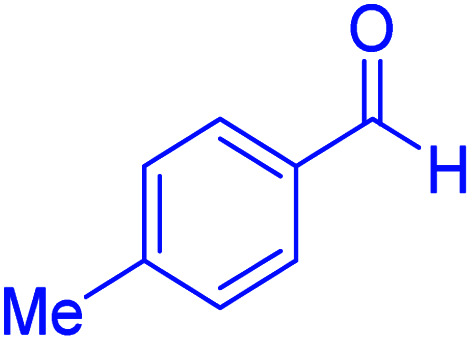	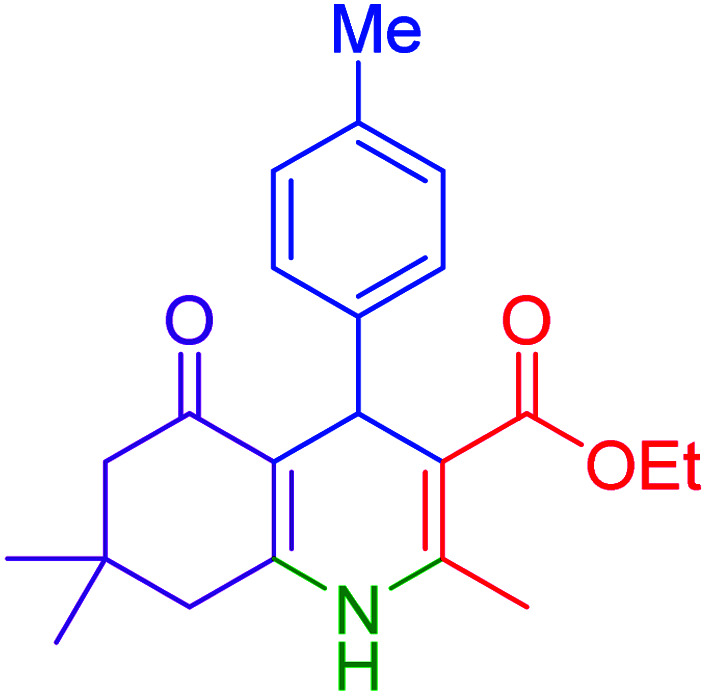	35	95	194–195	193–194 (ref. 20)
4	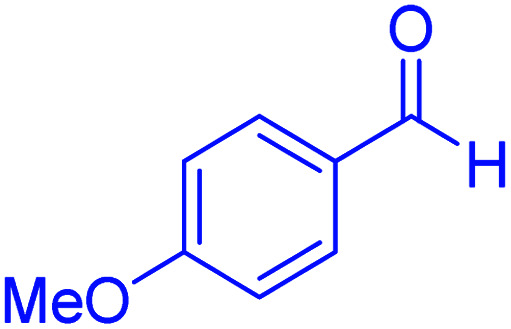	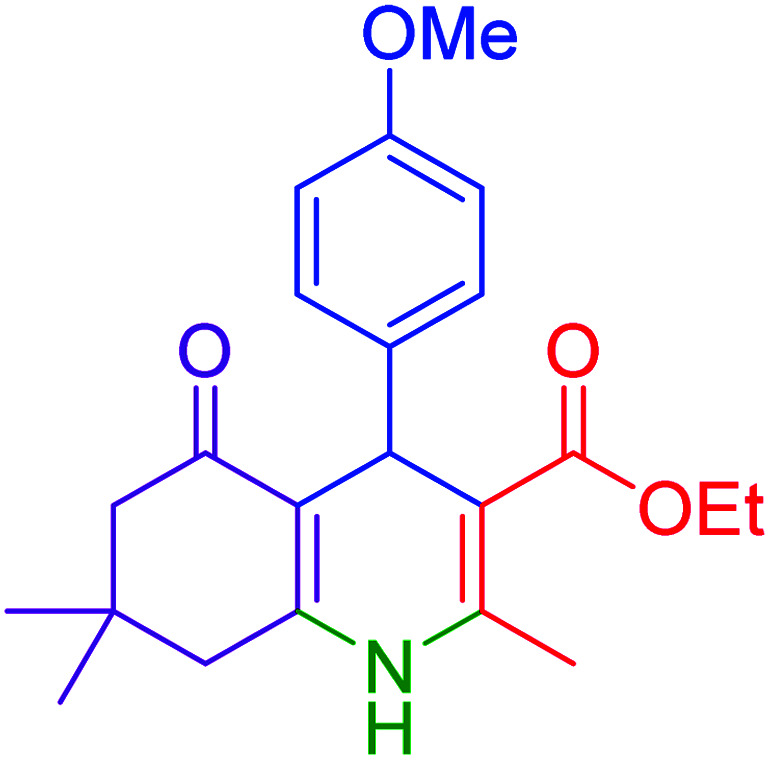	30	97	257–260	257–259 (ref. 20)
5	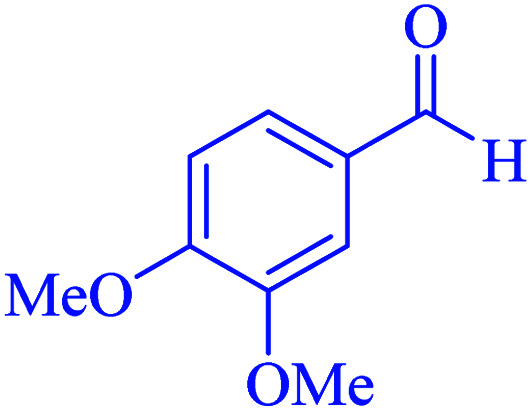	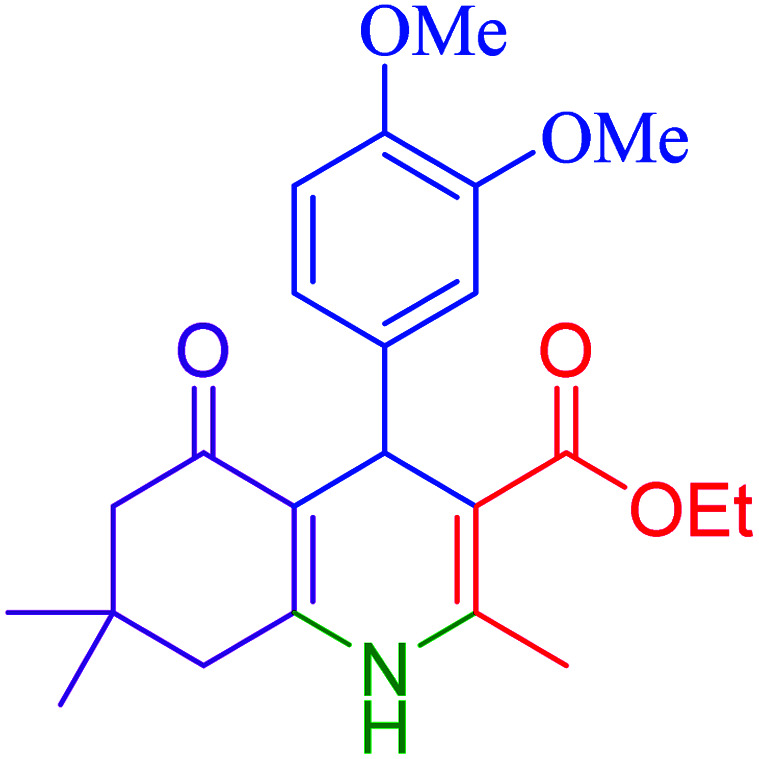	75	91	199–201	200–201 (ref. 48)
6	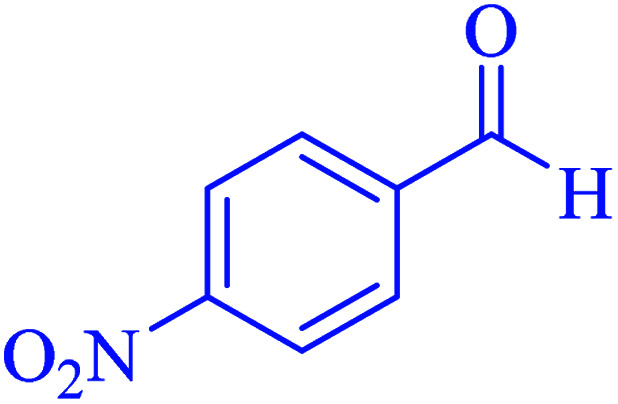	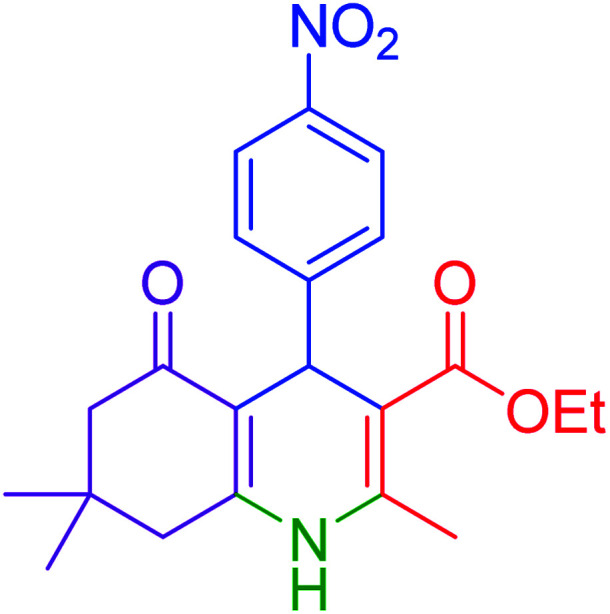	15	97	234–236	235–237 (ref. 20)
7	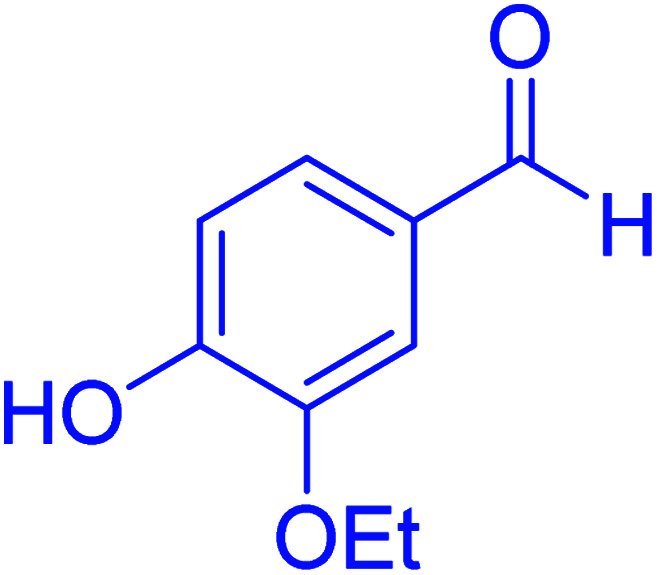	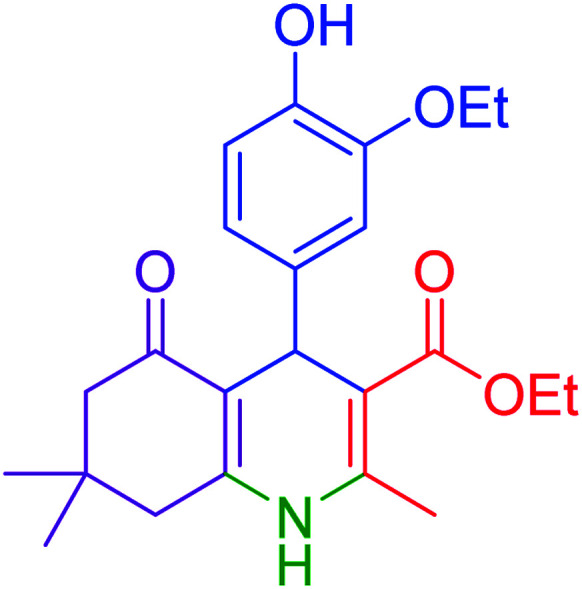	75	92	197–199	198–200 (ref. 49)
8	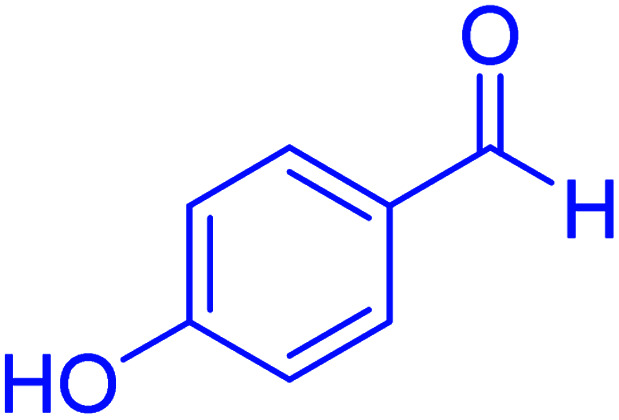	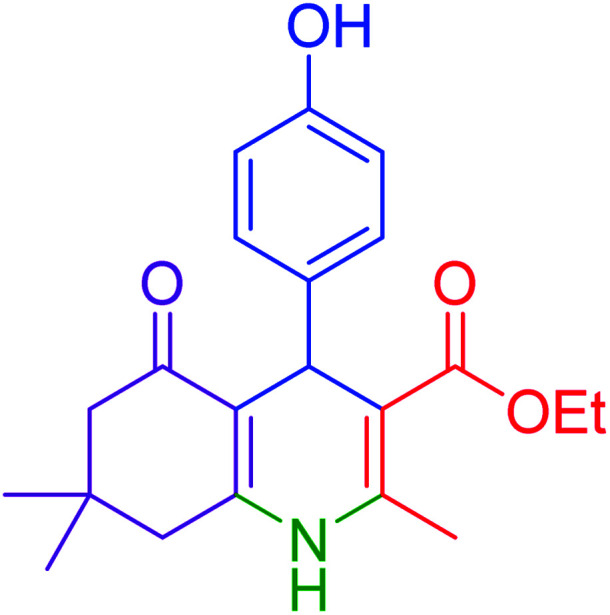	95	92	225–228	225–227 (ref. 20)
9	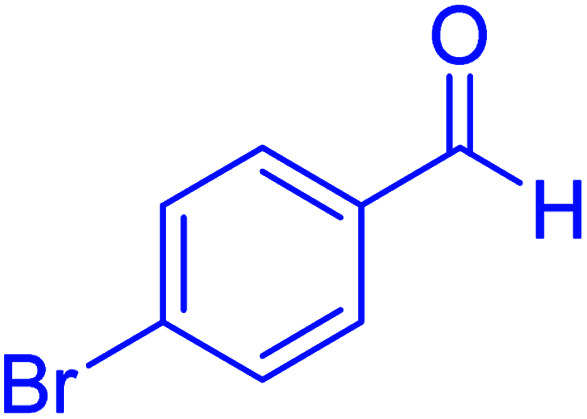	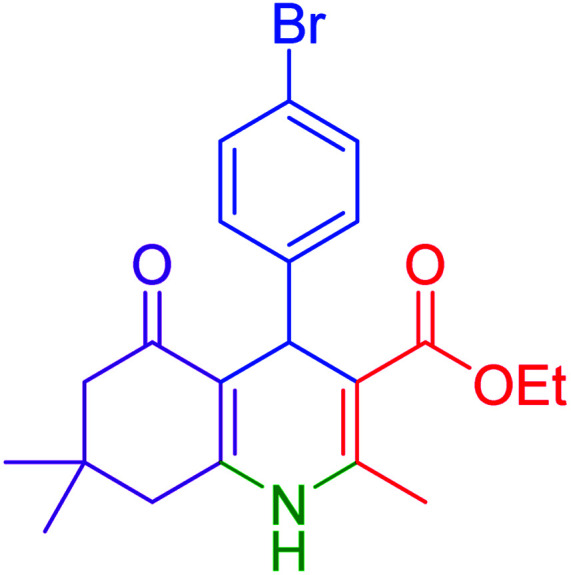	30	94	249–252	248–250 (ref. 20)
10	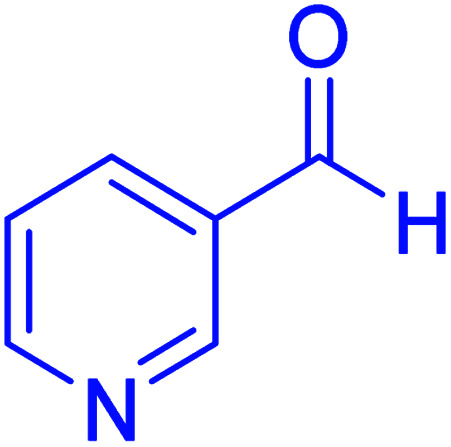	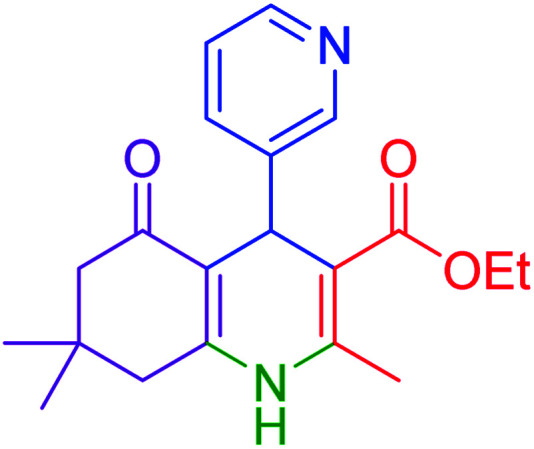	50	93	230–233	232–235 (ref. 20)
11	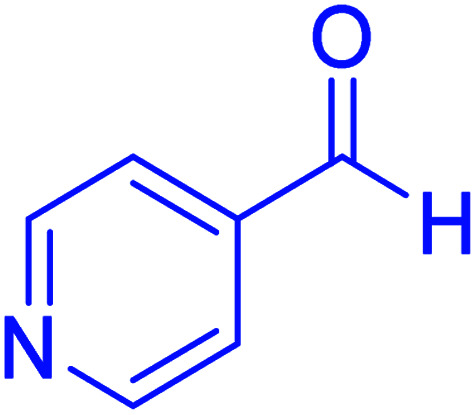	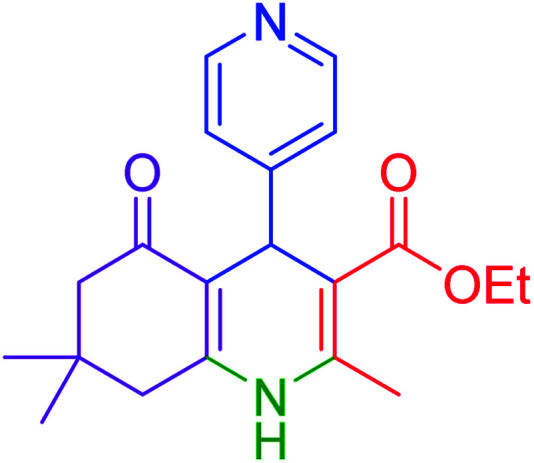	40	94	218–220	218–220 (ref. 50)
12	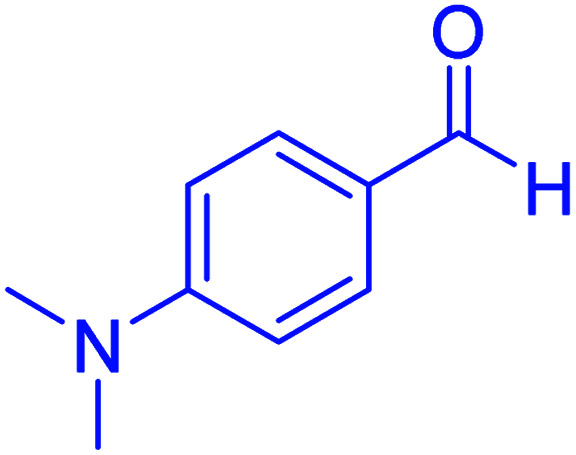	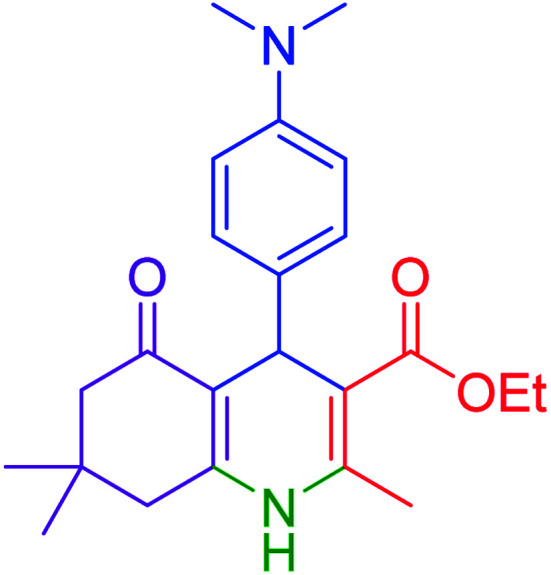	55	94	234–237	234–236 (ref. 51)
13	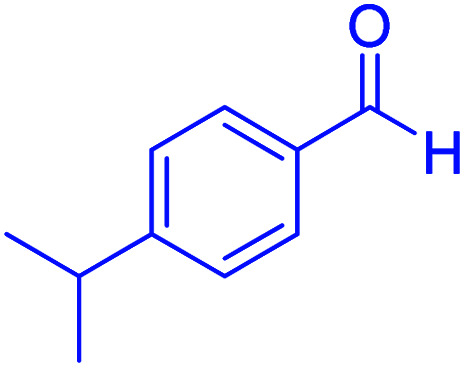	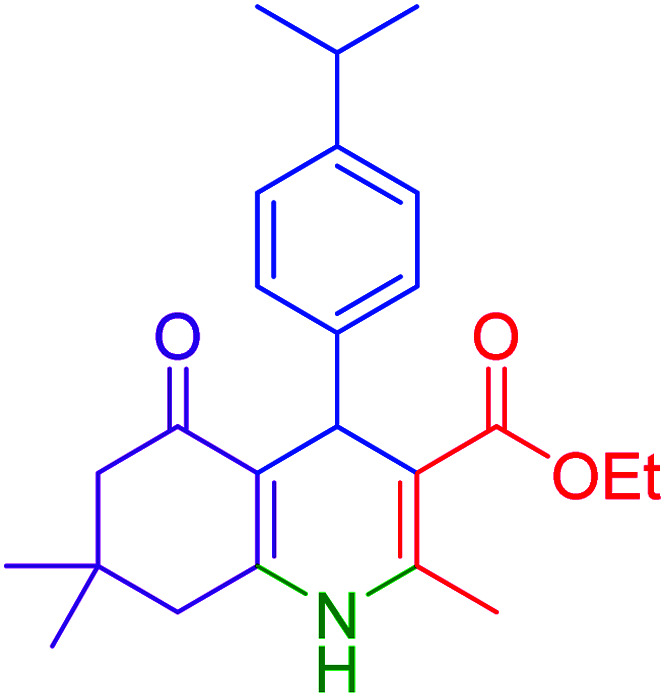	80	92	182–184	181–183 (ref. 48)
14	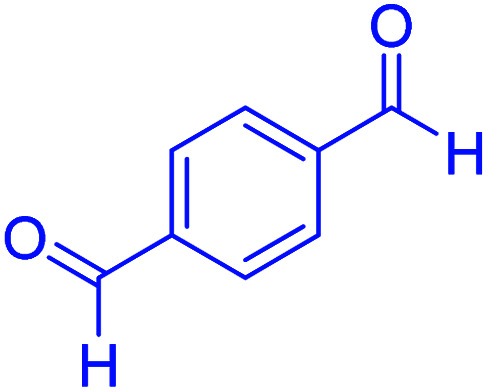	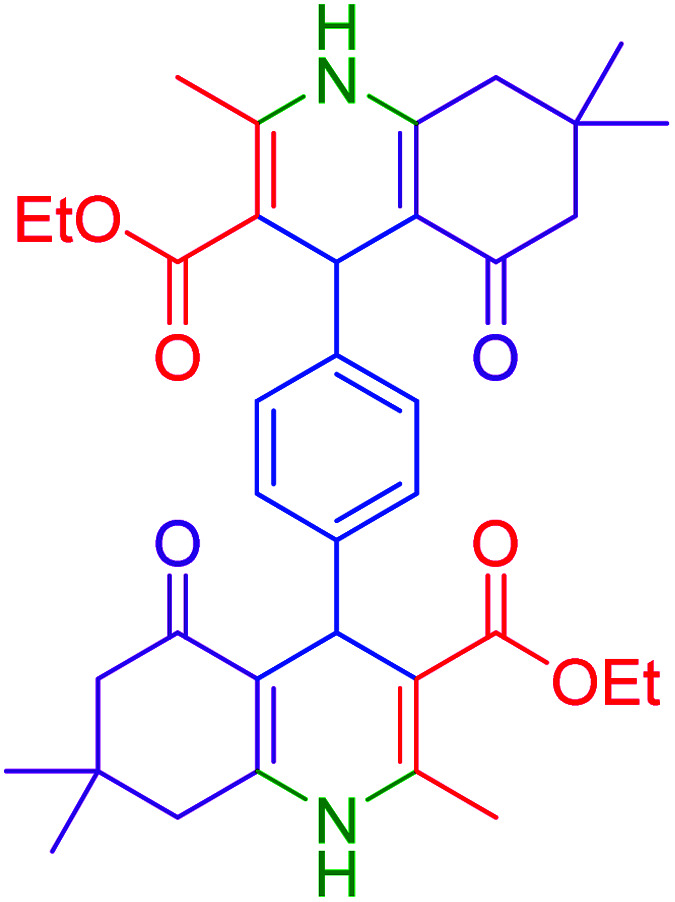	115	82	305–307	305–307 (ref. 52)

a Isolated yield.

b Reaction conditions: aromatic aldehyde (1 mmol), dimedone (1 mmol), ethyl acetoacetate (1 mmol), ammonium acetate (1.2 mmol), hercynite@sulfuric acid (7 mg) at 100 °C under solvent-free conditions.

In general, there are two plausible mechanistic pathways for the synthesis of polyhydroquinolines, following the Hantzsch reaction which is presented in Scheme 2.^6^ Both of the plausible mechanistic pathways involve the production of an enamine intermediate (from the combination of NH_3_ with active carbonyl compounds (ethyl acetoacetate or dimedone)) and Tandem Knoevenagel condensation – Michael addition–cyclization reactions. Both of the pathways are possible; but, the cyclic compounds exhibit enhanced acidity relative to acyclic models.^53^ Thus, based on more acidity of the cyclic compounds, the malononitrile was rapidly deprotonated with a greater speed in comparison to ethyl acetoacetate. Afterwards, it underwent Knoevenagel condensation reaction with aldehyde to form an α,β-unsaturated compound. Subsequently, the Michael addition was occurred and, in this conjugate addition reaction, the enamine acted as a Michael donor and attacked the α,β-unsaturated carbonyl intermediate as the Michael acceptor, followed by cyclization reaction which generated a six-membered N-containing ring. Finally, the dehydration gave the final polyhydroquinoline products (Scheme 2).^16^

**Scheme 3:**
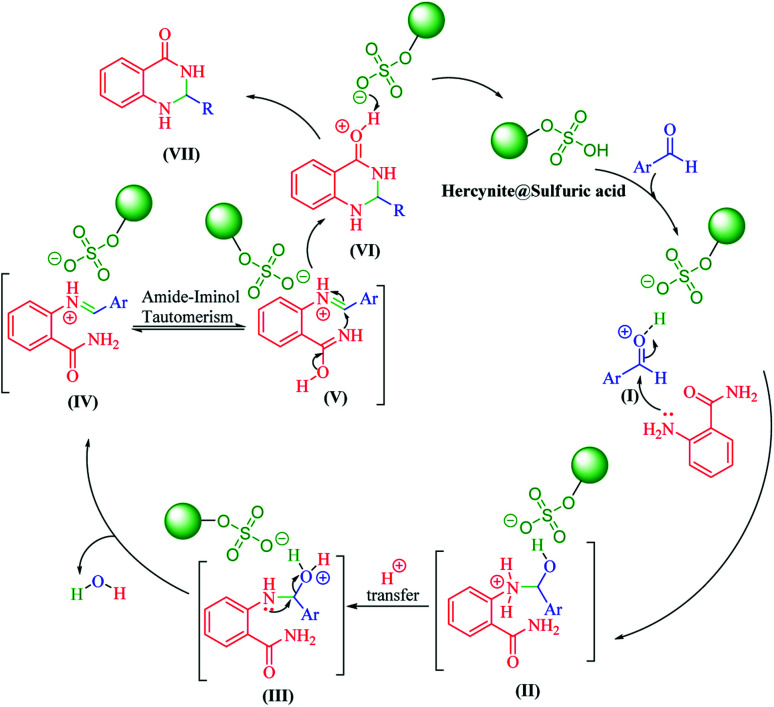
The proposed mechanism for the synthesis of 2,3-dihydroquinazolin-4(1*H*)-ones in the presence of hercynite@sulfuric acid MNPs.

In the next part of this research project, we applied this magnetic nanomaterial as a novel solid acid catalyst for the cyclocondensation of aromatic aldehydes with anthranilamide as a green reagent for the synthesis of 2,3-dihydroquinazolin-4(1*H*)-ones under diverse conditions (Tables 3 and 4). Besides, it is worth mentioning that, on the basis of the cyclic mechanism, this reaction gives only water as the by-product (Scheme 3).We optimized conditions of the reaction for the synthesis of 2,3-dihydroquinazolin-4(1*H*)-one scaffolds using *para*-chlorobenzaldehyde and anthranilamide as the model reaction. Afterwards, various parameters of the reaction, including the amount of catalyst, solvent and temperature, were evaluated for the model reaction, the results of which are summarized in Table 3. When there is no catalyst in the reaction, no reaction takes place (Table 3, entry 1). In this sense, it is worth mentioning that the existence of hercynite@sulfuric acid MNPs is required for this type of condensation reaction. The reaction proceeds faster by increasing the catalyst amount up to 9 mg. According to the results, 9 mg of the catalyst is required for the reaction. Using smaller amounts of the catalyst will cause the reaction to be incomplete (Table 3, entries 3–5). Increasing the amount of catalyst by more than 9 mg does not affect the efficiency percentage (Table 3, entry 7). Additionally, the catalytic effect of hercynite was investigated on the model reaction (Table 3, entry 2). But, it did not show an interesting effect on the reaction yield in comparison to its sulfonated form (hercynite@sulfuric acid MNPs) at the same conditions, which shows that the presence of SO_3_H functional groups in the catalyst plays an important role in promoting the reactions according to the proposed mechanism (see Scheme 3). Among various solvents used in the reaction, the results indicate that the ethanol as a solvent showed a higher efficiency, as compared to all solvents tested with 98% isolated yield (Table 3, entry 7). Finally, the reaction was performed in different temperatures, and the low temperature continued with lower efficiency (Table 3, entries 14–16). Regarding the optimization studies, the optimum conditions for this reaction are: 9 mg of hercynite@sulfuric acid MNPs in ethanol at reflux conditions (Table 3, entry 7).

**Table 3:** Optimization of the reaction conditions for the cyclocondensation of 4-chlorobenzaldehyde with anthranilamide as the model reaction for the synthesis of 2,3-dihydroquinazolin-4(1*H*)-ones

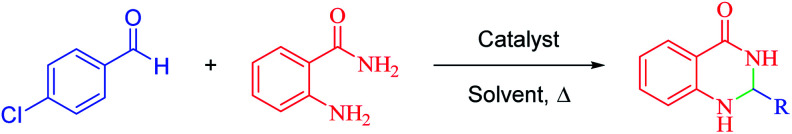						
Entry	Catalyst (mg)	Amount catalyst (mg)	Solvent	Temperature (°C)	Time (min)	Yield^a^^,^^b^ (%)
1	—	—	EtOH	Reflux	4 h	NR
2	Hercynite	9	EtOH	Reflux	15	Trace
3	Hercynite@sulfuric acid	4	EtOH	Reflux	15	64
4	Hercynite@sulfuric acid	6	EtOH	Reflux	15	81
5	Hercynite@sulfuric acid	8	EtOH	Reflux	15	93
6	Hercynite@sulfuric acid	9	EtOH	Reflux	15	98
7	Hercynite@sulfuric acid	10	EtOH	Reflux	15	98
8	Hercynite@sulfuric acid	9	Solvent-free	80	15	87
9	Hercynite@sulfuric acid	9	Solvent-free	100	15	91
10	Hercynite@sulfuric acid	9	PEG-400	80	15	89
11	Hercynite@sulfuric acid	9	H_2_O	Reflux	15	Trace
12	Hercynite@sulfuric acid	9	MeOH	Reflux	15	94
13	Hercynite@sulfuric acid	9	DMSO	80	15	87
14	Hercynite@sulfuric acid	9	EtOH	25	15	Trace
15	Hercynite@sulfuric acid	9	EtOH	50	15	71
16	Hercynite@sulfuric acid	9	EtOH	75	15	94

a Isolated yield.

b Reaction conditions: 4-chlorobenzaldehyde (1 mmol), anthranilamide (2-aminobenzamide) (1 mmol), catalyst (mg) and solvent (3 mL).

**Table 4:** Cyclocondensation of aromatic aldehydes with anthranilamide for synthesis of 2,3-dihydroquinazolin-4(1*H*)-one derivatives in the presence of hercynite@sulfuric acid catalyst EtOH under reflux conditions

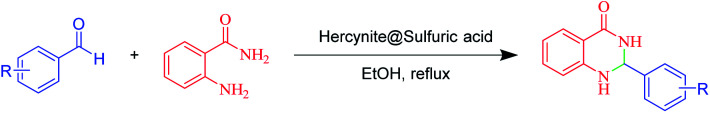						
Entry	Aryl aldehyde	Product	Time (min)	Yield^a^^,^^b^ (%)	Melting point	
					Measured	Literature
1	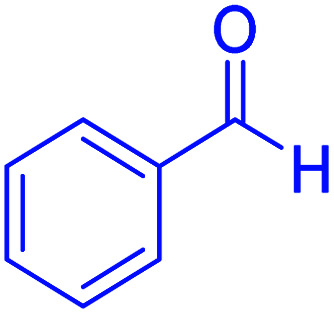	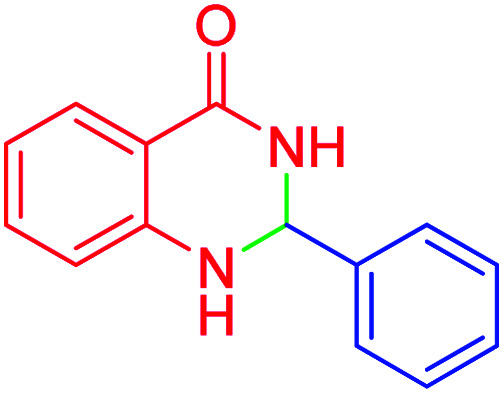	60	94	166–168	166–167 (ref. 20)
2	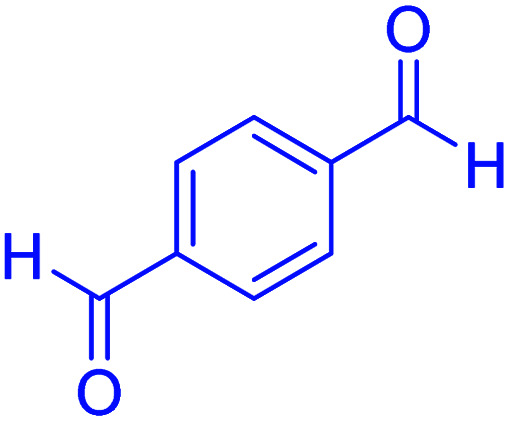	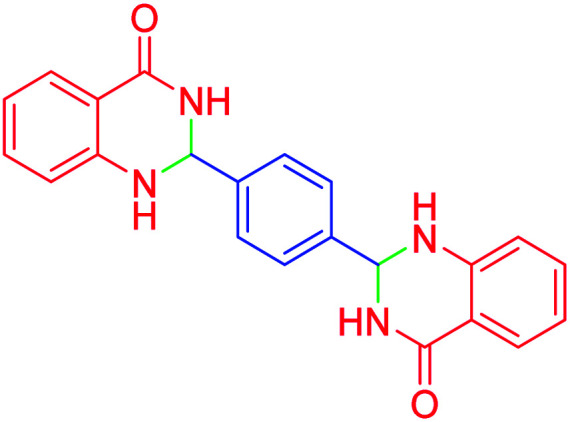	95	89	242–245	242–245 (ref. 20)
3	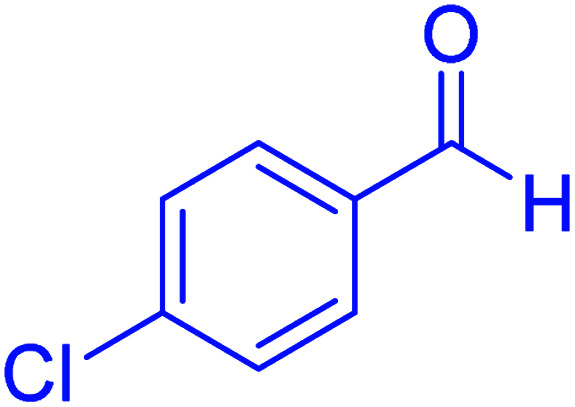	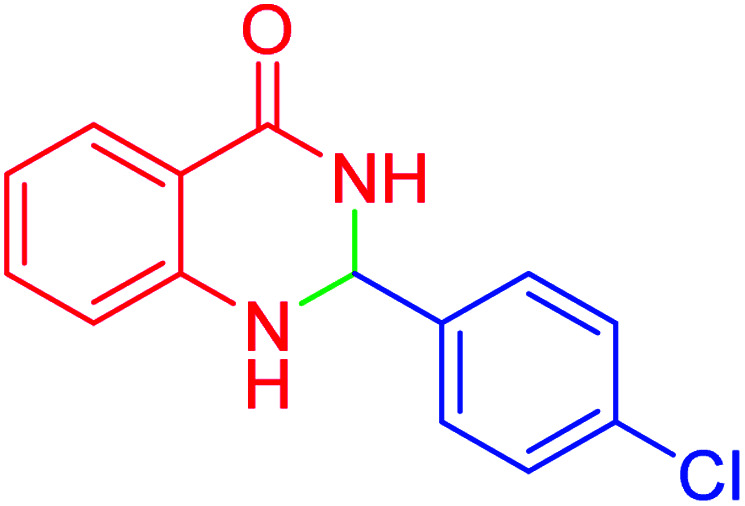	15	98	191–193	193–194 (ref. 20)
4	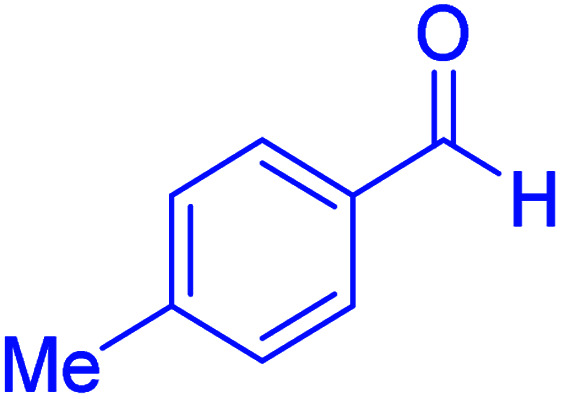	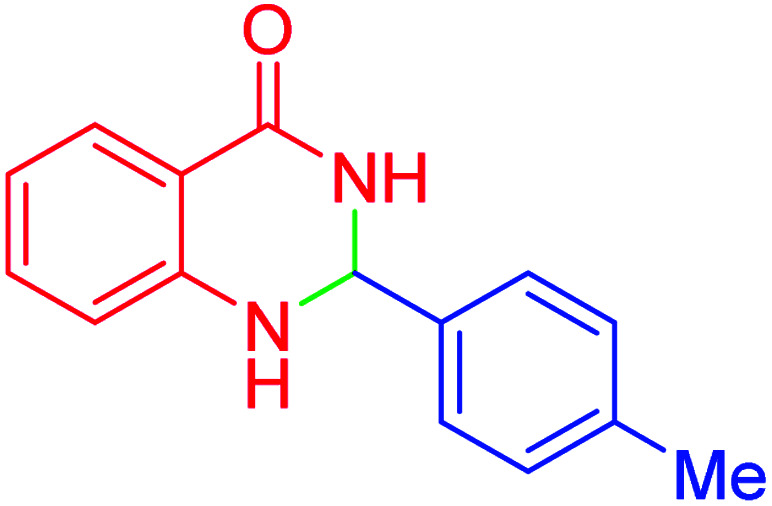	65	89	219–220	218–221 (ref. 20)
5	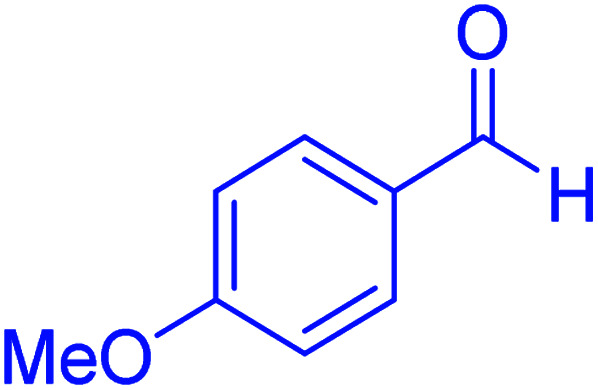	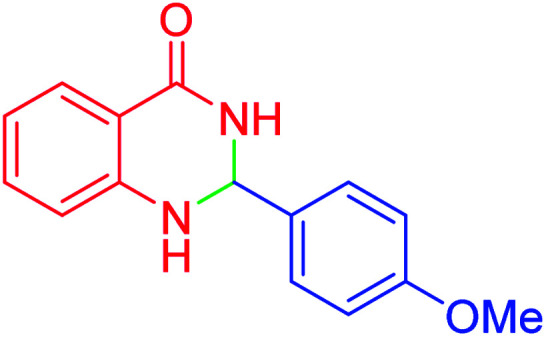	55	91	183–185	182–184 (ref. 20)
6	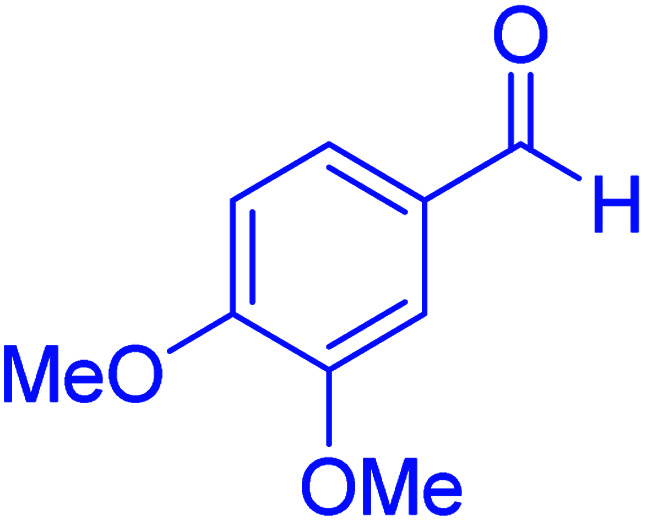	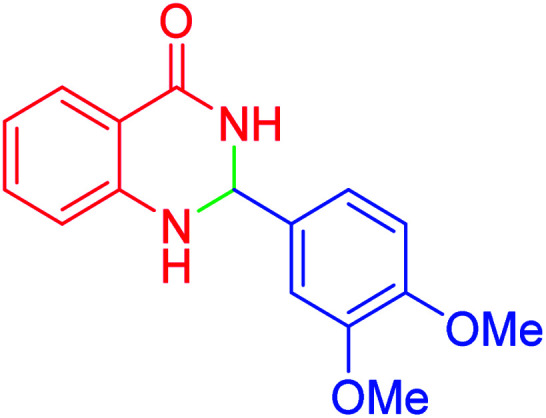	120	95	210–212	211–212 (ref. 20)
7	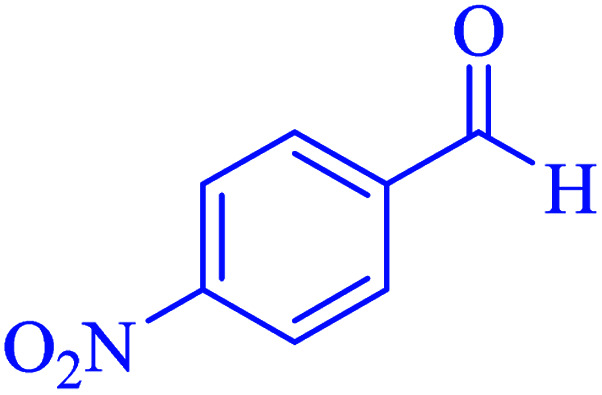	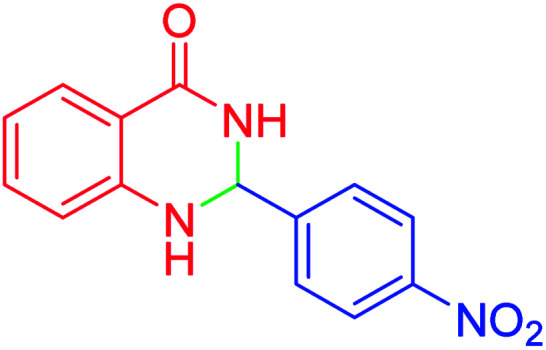	180	84	195–197	195–197 (ref. 20)
8	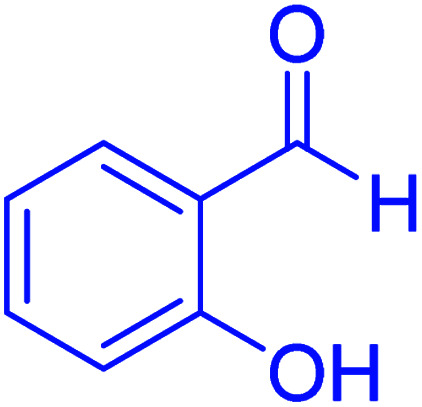	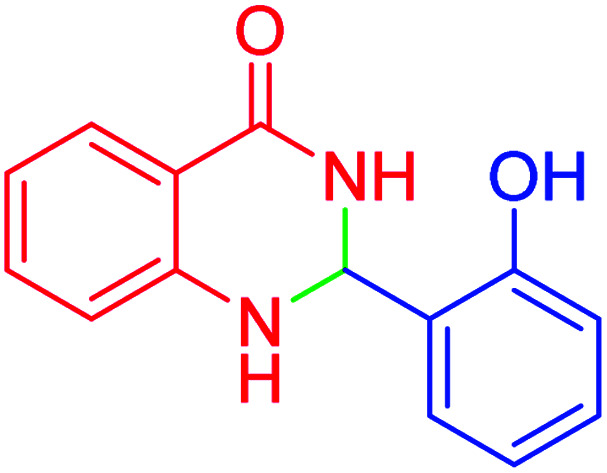	60	96	222–224	223–226 (ref. 54)
9	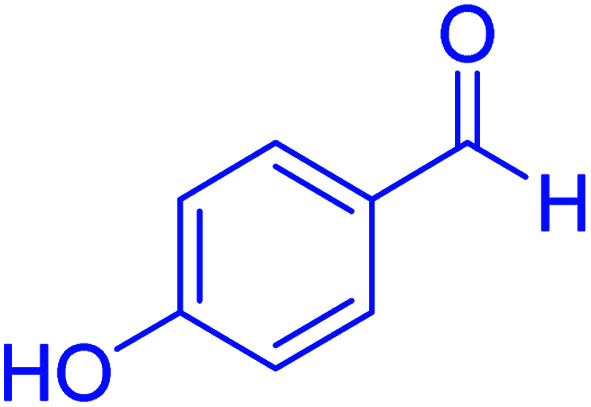	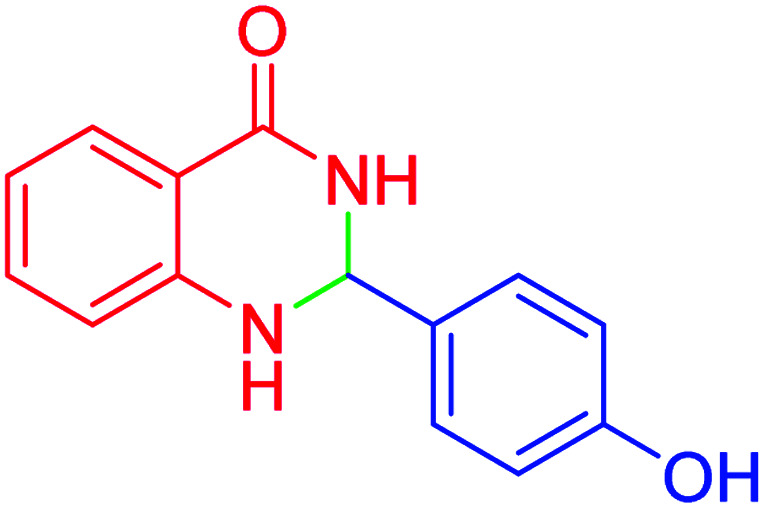	95	93	272–275	274–276 (ref. 20)
10	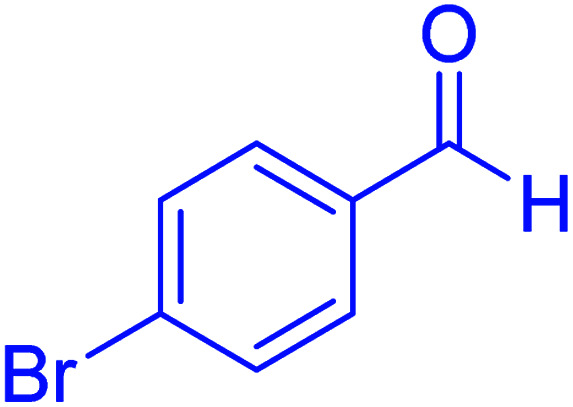	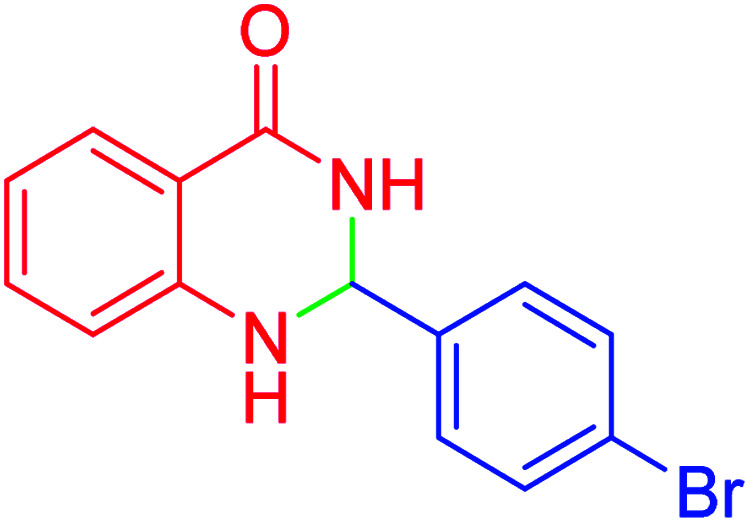	45	95	200–202	201–202 (ref. 20)
11	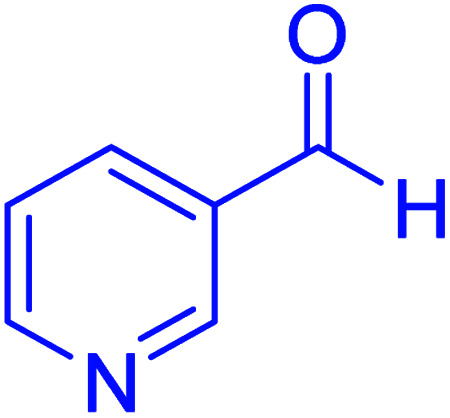	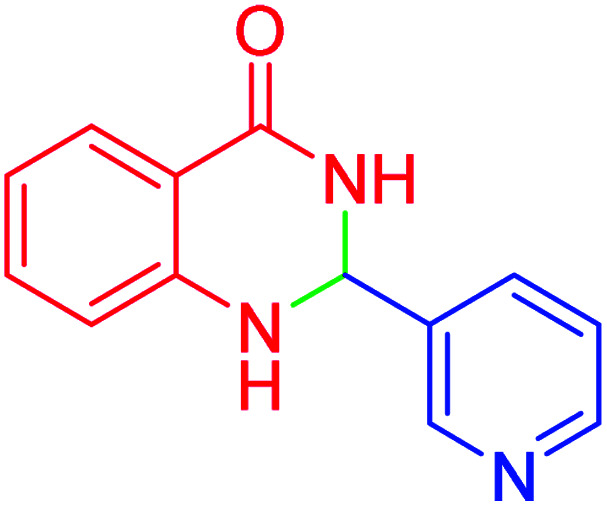	110	90	218–220	219–221 (ref. 55)
12	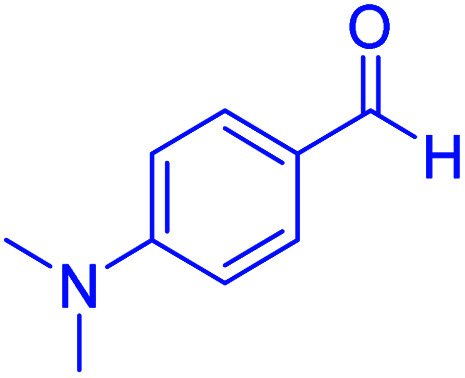	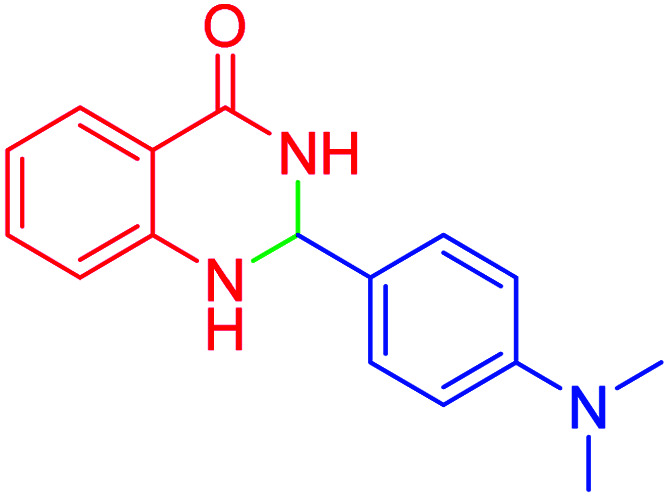	95	92	206–208	206–208 (ref. 56)

a Isolated yield.

b Reaction conditions: aromatic aldehyde (1 mmol), anthranilamide (2-aminobenzamide) (1 mmol), hercynite@sulfuric acid (9 mg) and ethanol (3 mL) under reflux conditions.

After optimizing the reaction conditions, we explored the scope of the reaction with various electron-donating and electron-withdrawing groups of aldehydes. In all cases, the products were made in high yields. Although the results indicate that hindered aryl aldehydes react slowly, as compared to the *para* isomers, electron-releasing and electron-withdrawing groups give excellent yield of products in short reaction times. The experimental details and the obtained results are mentioned in Table 4.

It is worth noting that the appropriate mechanism for the synthesis of 2,3-dihydroquinazolin-4(1*H*)-ones *via* the cyclocondensation of anthranilamide with aldehydes catalyzed by hercynite@sulfuric acid is described in Scheme 3.^20^ Firstly, the reaction was granted to start through the activation of the carbonyl group of the aldehyde *via* protonation by hercynite@sulfuric acid as a Brønsted acid catalyst. The second step involves the nucleophilic addition of the nitrogen of the anthranilamide's amino group (Ar-NH_2_) on the activated carbonyl group (I) with high electrophilicity to form the intermediate (II). Subsequently, the proton-transfer forms intermediate (III). Afterwards, an iminium ion intermediate (IV) is formed through the elimination of a water molecule from (III), followed by amide-iminol tautomerization (IV ⇌ V). The ring closure forming intermediate (VI) can be generated from intramolecular cyclization through the nucleophilic attack of the nitrogen on the carbon of the iminium ion. Eventually, the deprotonation of (VI) provides the corresponding 2,3-dihydroquinazolin-4(1*H*)-one product (VII) and, then, the catalyst re-enters the catalytic cycle.

### 3.4 Catalyst reusability studies

**Fig. 7:**
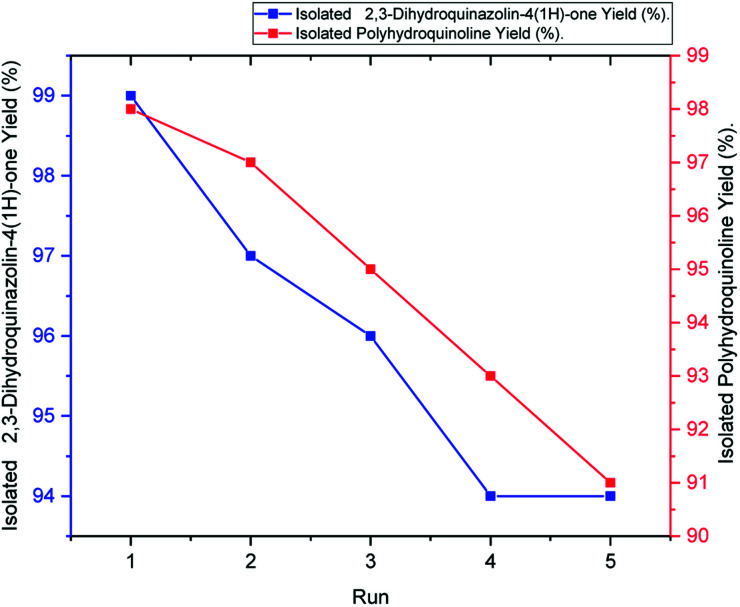
Reusability of hercynite@sulfuric acid MNPs in the synthesis of polyhydroquinoline (red line) and 2,3-dihydroquinazolin-4(1*H*)-ones (blue line).

Easy isolation and reusability of the heterogeneous catalyst put an end to the use of harmful and costly acid catalysts while decreasing the cost of products. Recyclability of the hercynite@sulfuric acid MNPs was investigated in the Hantzsch synthesis of polyhydroquinolines and the synthesis of 2,3-dihydroquinazolin-4(1*H*)-ones model reactions. The catalyst was separated after completion of the reaction, washed with acetone and ethyl acetate and, then, dried for reuse in further cycles. The recycled catalyst was employed in the five sequential cycles. Moreover, the yield of the reaction was moderately decreased after the fifth run of the reactions, as illustrated in Fig. 7.

### 3.5. Hot filtration

Additionally, in order to study the true heterogeneity of the hercynite@sulfuric acid MNPs and chances of leaching of sulfuric acid cites and also to check the type of catalyst absorption on the hercynite surface, a hot filtration test was conducted with the Hantzsch model reaction under the optimized reaction condition. While running the fresh batch, the catalyst was isolated off at 10 min when it was of 68% yield and the catalyst-free reaction mixture was stirred for another 10 min keeping other conditions same. Incidentally, the reaction afforded no augmentation in its yield. This, in turn, also signifies that no active catalytic species was leached out of the nanocomposite and the catalyst can be considered as a true heterogeneous catalyst. Furthermore, it shows that the hercynite@sulfuric acid played a catalytic role in the reaction without the sulfuric acid leaching into the solution or framework degradation. Besides, the stability of the sulfuric acid groups on the surface of hercynite support confirms the covalent adsorption of SO_3_H on the catalytic support.

### 3.6. Comparison

In the last part of our studies, in order to demonstrate the profit of nanoporous hercynite@sulfuric acid as a heterogeneous catalyst in Hantzsch synthesis of polyhydroquinolines and the synthesis of 2,3-dihydroquinazolin-4(1*H*)-ones, our resultant and reaction conditions were compared with those of the reported acid, base and metal catalysts (Table 5). As depicted in Table 5, the nanoporous hercynite@sulfuric acid is the most efficient catalyst for the mentioned reactions. Significantly, most of the reported methods toil from the absence of commonness for the condensation reactions of the deactivated aldehydes. In addition, the reported synthetic paths have some limitations, such as requiring extreme temperature or long duration, large amounts of the catalyst, and most importantly, the use of hazardous solvents to give excellent yields.

**Table 5:** Comparison of the synthesis of polyhydroquinolines and 2,3-dihydroquinazolin-4(1*H*)-ones in the presence of various catalysts

Entry	Reaction	Catalyst	Time (min)	Yield^a^ (%)	Ref
1	Polyhydroquinoline	FeAl_2_O_4_	180	90	45
2	Polyhydroquinoline	CoFe_2_O_4_@Pr	145	96	57
3	Polyhydroquinoline	Fe_3_O_4_@D–NH–(CH_2_)_4_–SO_3_H	90	86	58
4	Polyhydroquinoline	AIL-SCMNPs	15	80	59
5	Polyhydroquinoline	Fe_3_O_4_@GA@IG	45	89	60
6	Polyhydroquinoline	SBA-15@*n*-Pr-THAM-Zr	45	98	20
7	Polyhydroquinoline	Hercynite@sulfuric acid	20	99	This work
8	2,3-Dihydroquinazolin-4(1*H*)-one	α-d-glucose	180	61	61
9	2,3-Dihydroquinazolin-4(1*H*)-one	SBA-16/GPTMS-TSC-Cu^I^	35	95	62
10	2,3-Dihydroquinazolin-4(1*H*)-one	CoFe_2_O_4_@Pr	60	97	63
11	2,3-Dihydroquinazolin-4(1*H*)-one	Amberlyst-15	60	85	64
12	2,3-Dihydroquinazolin-4(1*H*)-one	Lactic acid	30	90	65
13	2,3-Dihydroquinazolin-4(1*H*)-one	SBA-15@*n*-Pr-THAM-Zr	35	98	20
14	2,3-Dihydroquinazolin-4(1*H*)-one	Hercynite@sulfuric acid	15	98	This work

a Isolated yield.

## 4. Conclusion

In summary, we have developed a green method for the synthesis of silica sulfuric acid functionalized hercynite MNP as an advanced magnetic solid acid catalyst using Zolfigol's method. The core-shell-like environment in the hercynite@sulfuric acid composite provided rigidity. Moreover, the high concentration of the surface of OH groups helps to anchor the incoming SO_3_H functional groups. The catalytic cites generated at the outer layer are also stabilized by the obtained covalent bonds. Physicochemical features of the as-engineered material (hercynite@sulfuric acid) were assessed *via* several analytical methods. While exploring its catalytic activity, we found it suitable in the multicomponent Hantzsch condensations of aromatic aldehyde with dimedone, ethyl acetoacetate and ammonium acetate towards a wide range polyhydroquinoline derivatives under solvent-free conditions and the cyclocondensation of aromatic aldehydes with anthranilamide for the synthesis of 2,3-dihydroquinazolin-4(1*H*)-ones in ethanol under reflux conditions. Besides, it is worth mentioning that the reactions were highly productive. The role of sulfuric acid was inevitable as the bare hercynite catalyst failed to make significant impact on the reaction. After the reaction, the catalyst was easily retrieved by magnet and reused for five times with consistent catalytic reactivity. There was also no leaching of sulfuric acid species in the reaction medium, justifying its true-heterogeneity.

## Data availability

The data that support the findings of this study are available in the ESI† of this article.

## Conflicts of interest

The authors declare that they have no known competing financial interests or personal relationships that could have appeared to influence the work reported in this paper.

## Supplementary Material

RA-012-D1RA07381H-s001
