# Supplementary material for: Synthesis and characterization of novel hercynite@sulfuric acid and its catalytic applications in the synthesis of polyhydroquinolines and 2,3-dihydroquinazolin-4(1H)-ones
Source: RSC Adv. 2022 Jan 20;12(5):2770–87. doi: 10.1039/d1ra07381h (PMC8979139; doi:10.1039/d1ra07381h)
Supplement: RA-012-D1RA07381H-s001 [file RA-012-D1RA07381H-s001.pdf]

Electronic Supplementary Material (ESI) for RSC Advances.
This journal is © The Royal Society of Chemistry 2022

Supplementary information

Synthesis and characterization of novel hercynite sulfuric acid and its catalytic applications in the synthesis of polyhydroquinolines and 2,3-pihydroquinazolin-4(1H)-ones

Masoud Mohammadi[a], Arash Ghorbani-Choghamarani[b,*]

[a]Department of Chemistry, Faculty of Science, Ilam University, P.O. Box 69315516, Ilam, Iran.

[b]Department of Organic Chemistry, Bu–Ali Sina University, Hamedan, 6517838683, Iran.

*correspondence to A. Ghorbani-Choghamarani, Tel: +98 8138282807; Fax: +98 8138380709; E-mail address: arashghch58@yahoo.com or a.ghorbani@basu.ac.ir

Spectral Data:

Ethyl 4-(pyridin-3-yl)-2,7,7-trimethyl-5-oxo-1,4,5,6,7,8- hexayidroquinolin-3-carboxylate:

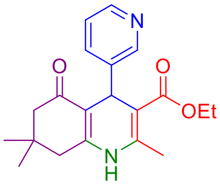

Mp = 230-233 °C, ^{1}H NMR (500 MHz, DMSO-d$_6$): δ = 0.80 (s, 3H), 0.99 (s, 3H), 1.09 (t, J = 7 Hz, 3H), 1.94–1.99 (m, 1H), 2.14–2.18 (m, 1H), 2.25-2.31 (m, 4H), 2.29–2.43 (m, 1H), 3.93-4.0 (q, J = 7 Hz, 2H), 4.82 (s, 1H), 7.20–7.22 (m, 1H), 7.46–7.48 (m, 1H), 8.23–8.27 (m, 1H), 8.35–8.36 (d, J = 5 Hz, 1H), 9.15 (s, 1H, NH). ^{13}C NMR (126 MHz, DMSO-d$_6$): δ = 14.54, 18.75, 26.87, 29.47, 32.63, 34.55, 50.53, 59.61, 103.11, 109.70, 123.71, 135.26, 143.20, 146.35, 147.37, 149.34, 150.35, 166.95, 194.69 ppm.

Ethyl 4-(4-dimethylamino)phenyl)-2,7,7-trimethyl-5-oxo-1,4,5,6,7,8- hexayidroquinolin-3-carboxylate:

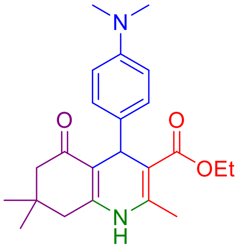

MP = 234-237 °C, ^{1}H NMR (500 MHz, DMSO-d_6): δ = 0.86 (s, 3H), 0.99 (s, 3H), 1.12–1.15 (t, J = 7.0 Hz, 3H), 1.93–1.97 (m, 1H), 2.12–2.15 (m, 1H), 2.24–2.28 (m, 4H), 2.37-2.40 (m, 1H), 2.78 (s, 6H), 3.94–4.01 (m, 2H), 4.72 (s, 1H), 6.52–6.54 (d, J = 10 Hz, 3H), 6.93–6.95 (d, J = 10 Hz, 3H), 8.93 (s, 1H), ^{13}C NMR (126 MHz, DMSO-d_6): δ = 14.66, 18.70, 27.05, 29.64, 32.58, 35.02, 50.80, 59.37, 104.74, 110.81, 112.48, 128.40, 136.53, 144.55, 149.12, 149.47, 167.54, 194.72 ppm.

Ethyl 2,7,7-trimethyl-5-oxo-4-(p-tolyl)-1,4,5,6,7,8-hexahydroquinoline-3- carboxylate:

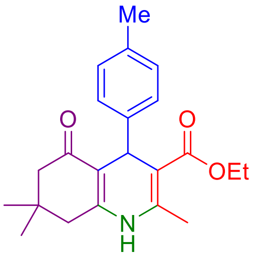

MP = 194-195 °C, ^{1}H NMR (500 MHz, DMSO) δ: 0.83 (s, 3H), 0.99 (s, 3H), 1.11-1.13 (t, 3H, J= 7 Hz), 1.93-1.96 (d, J= 15 Hz, 1H), 2.12-2.18 (m, 4H), 2.22-2.31 (m, 4H), 2.38-2-41 (d, J= 15 Hz, 1H),3.93-3.97 (q, 2H, J= 7 Hz), 4.80 (s, 1H), 6.95-6.96 (d, J= 5 Hz, 2H), 6.95-6.96 (d, J= 5 Hz, 2H), 7.01-7.02 (d, J= 5 Hz, 2H), 9.00 (s, 1H), ^{13}C NMR (126 MHz, DMSO-d_6): δ =14.61, 18.72, 21.02, 26.92, 29.61, 32.58, 35.84, 39.92, 50.72, 59.44, 104.24, 110.55, 127.82, 128.74, 134.97, 145.19, 145.25, 149.80, 167.35, 194.67 ppm.

2,7,7-Trimethyl-4-(4-nitro-phenyl)-5-oxo-1,4,4a,5,6,7,8,8a-octahydro-quinoline-3-carboxylic acid ethyl ester:

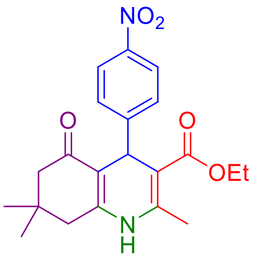

Mp = 234-236 °C. ^{1}H NMR (500 MHz, DMSO-d_6): δ = 0.81 (s, 3H), 0.99 (s, 3H), 1.08–1.11 (t, J = 7 Hz, 3H), 1.96–1.98 (d, J = 10 Hz, 1H,), 2.15–2.19 (m, 1H), 2.28–2.38 (m, 4H), 2.42 (m, 1H), 3.90–4.00 (m, 2H), 4.97 (s, 1H), 7.48–7.62 (m, 2H), 7.94–7.99 (m, 2H), 9.25 (s, 2H). 13C NMR (125 MHz, DMSO-d_6):

d = 14.44, 18.67, 26.75, 29.48, 31.61, 32.61, 36.89, 50.48, 59.67, 103.10, 109.68, 121.31, 122.49, 129.82, 134.76, 146.59, 147.82, 150.23, 150.59, 166.84, 194.74 ppm.

4-(4-Chloro-phenyl)-2,7,7-trimethyl-5-oxo-1,4,4a,5,6,7,8,8aoctahydro-quinoline-3-carboxylic acid ethyl ester:

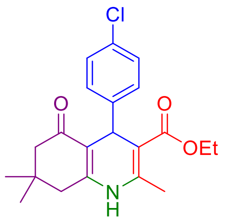

Mp = 234-245 °C. ^{1}H NMR (500 MHz, DMSO-d_6): δ = 0.81 (s, 3H), 0.98 (s, 3H), 1.10 (t, J = 7. Hz, 3H), 1.96 (d, J=20 Hz, 1H), 2.15 (d, J=20 Hz, 1H), 2.25–2.28 (m, 4H), 2.38–41 (m, 1H), 3.92–3.99 (q, J = 7. Hz, 2H), 4.84 (s, 1H), 7.14–7.16 (d, J = 10 Hz, 2 H), 7.21–7.23 (d, J = 10 Hz, 2 H) 9.09 (s, 1H). ^{13}C NMR (126 MHz, DMSO-d_6): δ =14.56, 18.75, 26.88, 29.53, 32.57, 36.06, 39.88, 50.63, 59.54, 103.58, 110.13, 128.12, 129.78, 130.65, 145.86, 147.02, 150.05, 167.10, 194.68 ppm.

Ethyl 1,4,5,6,7,8-hexahydro-4-(4-isopropylphenyl)-2,7,7-trimethyl-5-oxoquinoline-3-carboxylate:

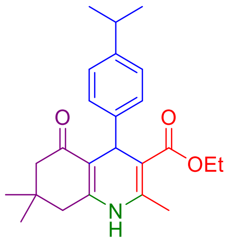

MP 182 – 184 °C, ^{1}H NMR (500 MHz, DMSO-d_6): δ = 0.86 (s, 3H), 0.98 (s, 3H), 1.10-1.19 (m, 9H), 1.95–1.99 (m, 1H), 2.12–2.16(m, 1H), 2.24(s, 3H), 2.28(m, 1H), 2.37(m, 1H), 2.71–2.79 (se, *J* = 5 Hz, 1H), 3.94–3.98 (q, *J* = 7 Hz, 2H), 4.82 (s, 1H), 6.98–7.06(m, 4H), 9.05 (s, *1*H); ^{13}C NMR (126 MHz, DMSO-d_6) δ =14.58, 18.71, 24.24, 24.39, 27.09, 29.51, 32.58, 33.40, 35.74, 50.71, 59.51, 104.39, 110.42, 126.06, 127.77, 145.12, 145.56, 146.01, 150.16, 167.43, 194.91 ppm.

Ethyl-2,7,7-trimethyl-5-oxo-4-(pyridin-4-yl)-1,4,5,6,7,8-hexahydroquinoline-3-carboxylate:

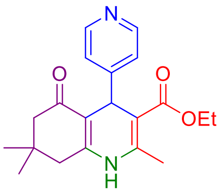

M.p: 218-220 °C; ^{1}H NMR (500 MHz, DMSO-d$_6$): δ = 0.80 (s, 3H), 0.98 (s, 3H), 1.07-1.10 (t, J = 7 Hz, 3H), 1.98 (m, 1H), 2.16 (m, 1H), 2.27–2.34 (m, 4H), 2.40 (m, 1H), , 3.94-3.98 (q, J = 7 Hz, 2H), 4.85 (s, 1H), 7.12 (d, J = 5 Hz, 2H), 8.37 (s, 2H), 9.19 (s, 1H); ^{13}C NMR (126 MHz, DMSO-d$_6$) δ = 194.73, 166.90, 155.82, 150.66, 149.68, 146.70, 123.27, 109.21, 102.42, 59.66, 50.53, 39.84, 36.25, 32.56, 29.46, 26.83, 18.75, 14.52.

Ethyl 2,7,7-trimethyl-5-oxo-4-phenyl-1,4,5,6,7,8-hexahydroquinolin-3- carboxylate:

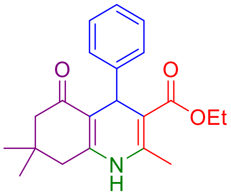

M.p: 201-203 °C; ^{1}H NMR (500 MHz, DMSO-d$_6$): δ = 0.83 (s, 3H), 0.99 (s, 3H), 1.09–1.12 (t, J = 7.0 Hz, 3H), 1.96 (d, 1H), 2.15 (d, 1H), 2.23-2.31(m, 4H), 2.38–2.42 (m, 1H), 3.93-3.98 (q, J = 7 Hz, 2H), 4.84 (s, 1H), 7.3-7.06 (m, 1H), 7.11 – 7.19 (m, 4H), 9.04(s, 1H); ^{13}C NMR (126 MHz, DMSO-d$_6$) δ = 194.70, 167.30, 149.95, 148.10, 145.43, 128.16, 127.91, 126.12, 110.43, 104.07, 59.47, 50.70, 39.92, 36.31, 32.58, 29.58, 26.90, 18.73, 14.59.

Ethyl 4-(4-methoxyphenyl)-2,7,7-trimethyl-5-oxo-1,4,5,6,7,8- hexahydroquinoline-3-carboxylate

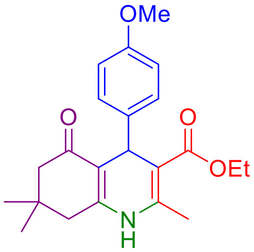

M.p: 257-260 °C; ^{1}H NMR (500 MHz, DMSO-d$_6$): δ = 0.83 (s, 3H), 0.99 (s, 3H), 1.08–1.11 (t, J = 7.0 Hz, 3H), 1.86-1.90 (d, 1H), 2.08-2.14 (d, 1H), 2.17-2.25 (m, 4H), 2.36–2.39 (m, 1H), 3.68 (s, 3H), 3.87-3.96

(m, 2H), 5.03 (s, 1H), 6.72-6.75 (m, 1H), 6.79 – 6.82 (m, 1H), 7.01-7.04 (m, 1H), 7.08-7.09 (m, 1H), 8.92 (s, 1H); ^{13}C NMR (126 MHz, DMSO-d_6) δ = 194.27, 167.75, 157.60, 150.42, 144.57, 135.42, 130.96, 127.39, 119.93, 111.45, 109.13, 103.39, 59.21, 55.62, 50.85, 33.27, 32.43, 29.77, 26.64, 18.49, 14.54.

Ethyl 4-(4-bromophenyl)-2,7,7-trimethyl-5-oxo-1,4,5,6,7,8-hexahydroquinoline-3 carboxylate:

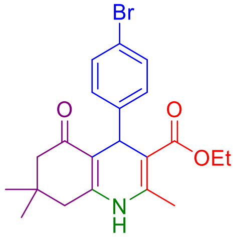

M.p: 249-252 °C; ^{1}H NMR (500 MHz, DMSO-d_6): δ = 0.85 (s, 3H), 1.0 (s, 3H), 1.13 (b, s, 3H), 1.95-1.98 (d, 1H), 2.19 (s, 1H), 2.28 (s, 3H), 2.39 (m, 1H), 3.97 (b, s, 2H), 4.82 (s, 1H), 6.97-7.04 (m, 4H), 9.01 (s, 1H); ^{13}C NMR (126 MHz, DMSO-d_6) δ = 14.61, 18.73, 21.02, 26.93, 29.62, 32.58, 35.87, 50.74, 59.44, 104.27, 110.58, 127.84, 128.74, 134.97, 145.20, 145.27, 149.82, 167.35, 194.68 ppm.

Ethyl 2,7,7-trimethyl-5-oxo-4-(4-hydroxy)-1,4,5,6,7,8-hexahydroquinoline-3- carboxylate:

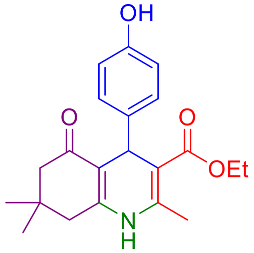

M.p: 225-228 °C; ^{1}H NMR (500 MHz, DMSO-d_6): δ = 0.85 (s, 3H), 1.00 (s, 3H), 1.12- 1.15 (t, J= 7 Hz, 3H), 1.95-1.98 (d, J= 15 Hz, 1H), 2.13-2.16 (d, J= 15 Hz, 1H), 2.26-2.28 (m, 4H), 2.38-2.41 (m, 1H), 2.40-2.44 (d, J= 16Hz, 1H), 3.96-3.98 (q, J= 7 Hz, 2H), 4.74 (s, 1H), 6.54-6.56 (m, 2H), 6.92-6.93 (m, 2H), 8.92 (s, 1H), 9.02 (s, 1H); ^{13}C NMR (126 MHz, DMSO-d_6) δ = 14.64, 18.71, 26.94, 29.63, 32.59, 35.25, 39.94 50.78, 59.41,104.58, 110.80, 114.90, 128.79, 138.88, 144.84, 149.58, 155.70, 167.48, 194.74 ppm.

***Dimethyl 4-(3-ethoxy-4-hydroxyphenyl)-2,6-dimethyl-1,4-dihydropyridine-3,5-dicarboxylate*:**

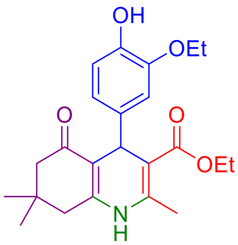

Mp: 197-199 °C; ^{1}H NMR (500 MHz, DMSO-d_6): δ = 0.87 (s, 3H), 1.00 (s, 3H), 1.11-1.16 (t, 3H, *J* = 7 Hz), 1.27-1.30 (t, 3H, *J* = 7 Hz), 1.96-1.99 (d, 1H), 2.14-2.18 (d, 1H), 2.22-2.28 (m, 4H), 2.39-2.42(d, 1H), 3.85-3.93 (m, 2H), 3.97-4.01 (q, J = 7 Hz, 2H), 4.73 (s, 1H), 6.50 (d, 1H, *J* = 7 Hz), 6.58 (d, 1H, *J* = 7 Hz), 6.64 (s, 1H); ^{13}C NMR (126 MHz, DMSO-d_6) δ = 14.69, 15.25, 18.70, 26.84, 29.69, 32.57, 35.44, 50.78, 59.44, 64.24, 104.49, 110.68, 114.01, 115.47, 120.12, 139.42, 144.86, 145.35, 146.24, 149.67, 167.51, 194.82 ppm.

Ethyl 4-(3,4-dimethoxyphenyl)-2,7,7-trimethyl-5-oxo-1,4,5,6,7,8-hexahydroquinoline-3-carboxylate:

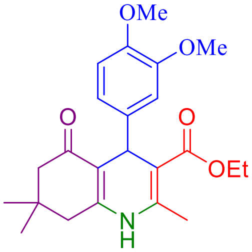

MP: 199-201 ºC; ^{1}H NMR (500 MHz, DMSO-d_6): δ = 0.88 (s, 3H), 1.01 (s, 3H, CH_3), 1.14-1.17 (t, J = 7. Hz, 3H), 1.97-2.00 (d, J = 5 Hz, 1H), 2.15-2.19 (d, J = 15 Hz, 1H), 2.27-2.30 (m, 4H), 2.40-2.44 (m, 1H), 3.66 (s, 3H), 3.66 (s, 3H), 3.97-4.02 (q, J = 7 Hz, 2H), 4.79 (s, 1H), 6.61-6.63 (t, J = 5 Hz, 1H), 6.74–6.77 (m, 2H), 9.02 (s, 1H). ^{13}C NMR (126 MHz, DMSO-d_6) δ = 14.71, 18.72, 26.89, 29.68, 32.58, 35.60, 50.75, 55.76, 55.87, 59.48, 104.33, 110.52, 111.94, 112.18, 119.70, 140.96, 145.07, 147.43, 148.45, 149.87, 167.44, 194.83 ppm.

1,4-bis(3-ethoxylcarbonyl-1,4,5,6,7,8-hexahydro-5-oxo-2,7,7-trimethylquinoline-4-yl)benzene:

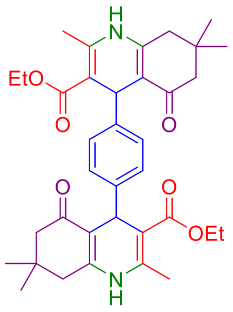

MP: 305-307 ºC; ^{1}H NMR (500 MHz, DMSO-d_6): δ = 0.84 (s, 6H), 0.98 (s, 6H), 1.07-1.14 (t, J =7 Hz, 6H), 1.92– 2.14 (m, 4H), 2.26 (s, 6H), 2.30-240 (m, 4H), 3.96-3.98 (q, H= 7 Hz, 4H), 4.81 (s, 2H), 6.90–6.97 (m, 4H), 9.00 (s, 2H); ^{13}C NMR (126 MHz, DMSO-d_6) δ = 14.49, 14.59, 18.71, 18.79, 26.61, 27.25, 29.36, 29.68, 32.60, 32.63, 35.28, 35.81, 50.73, 59.36, 59.47, 103.86, 104.27, 110.34, 110.57, 127.11, 127.28, 145.15, 145.45, 145.53, 150.09, 167.33, 167.40, 194.68, 194.82 ppm.

2-Phenyl-2,3-dihydroquinazolin-4(1*H*)-one:

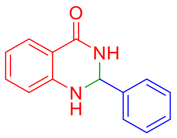

M.P: 166-168 °C; ^{1}H NMR (500 MHz, DMSO-d_6): δ = 5.76 (s, 1H); 6.68 (m, 1H), 6.76 (m, 1H), 7.10 (m, 1H), 7.26 (m, 1H), 7.33 (m, 1H), 7.40 (m, 1H), 7.50-7.64 (m, 3H), 8.19 (m, 1H), 8.29 (m, 1H); ^{13}C NMR (126 MHz, DMSO-d_6) δ = 46.50, 67.05, 114.72, 117.58, 127.33, 127.82, 128.78, 128.91, 129.08, 133.78, 135.09, 142.09, 148.34, 164.08 ppm.

2-(Pyridin-4-yl)-2,3-dihydroquinazolin-4(1*H*)-one:

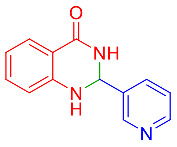

M.P: 218-220 °C; ^{1}H NMR (500 MHz, DMSO-d_6): δ = 5.86 (s, 1H,); 6.69-6.77 (m, 2H), 7.17 (s,1H), 7.25-7.29 (m, 1H), 7.41-7.44 (m, 1H), 7.62-7.64 (m, 1H), 7.85-7.91 (m, 1H), 8.38 (s, 1H), 8.55-8.56 (m, 1H), 8.67-

8.68 (m, 1H); ^{13}C NMR (126 MHz, DMSO-d_6) δ = 65.15, 115.03, 115.50, 117.98, 124.01, 127.87, 133.94, 135.13, , 137.29, 148.17, 148.84, 150.13, 164.05 ppm.

2-(4-chlorophenyl)-2, 3-dihydroquinazolin-4(1*H*)-one:

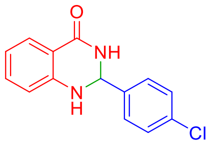

M.P: 193-191°C; ^{1}H NMR (500 MHz, DMSO-d_6): δ = 5.77 (s, 1 H), 6.78 (m, 1H), 6.76 (m, 1 H), 7.14 (s, 1H), 7.23-7.27 (m, 1 H), 7.41-7.55 (m, 4 H), 7.60-7.64 (m, 1 H), 8.33 (s, 1 H); ^{13}C NMR (126 MHz, DMSO-d_6) δ = 66.22, 114.93, 115.41, 117.74, 127.83, 128.77, , 129.22, 130.10, 133.86, 141.15, 148.11, 163.96 ppm.

2-(4-Bromophenyl)-2,3-dihydroquinazolin-4(1*H*)-one:

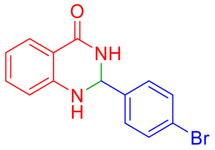

M.P: 200-202 °C; ^{1}H NMR (500 MHz, DMSO-d_6) δ = 6.34 (t, *J* = 2.2 Hz, 1H), 6.72 (t, *J* = 7.5 Hz, 1H), 6.78 (d, *J* = 8.1 Hz, 1H), 7.01 (s, 1H), 7.26 (td, *J* = 7.8, 1.7 Hz, 1H), 7.69 – 7.56 (m, 2H), 7.82 – 7.75 (m, 1H), 7.86 (dd, *J* = 7.9, 1.6 Hz, 1H), 8.07 (dd, *J* = 8.2, 1.3 Hz, 1H), 8.22 (s, 1H); ^{13}C NMR (126 MHz, DMSO-d_6) δ = 62.64, 114.98, 115.37, 118.14, 125.17, 127.78, 129.40, 130.35, 134.02, 134.37, 136.38, 147.58, 148.12, 163.83 ppm.

2-(4-Nitrophenyl)-2,3-dihydroquinazolin-4(1*H*)-one:

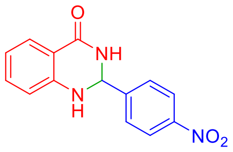

M.P: 195-197 °C; ^{1}H NMR (500 MHz, DMSO-d$_6$) δ = 5.92 (d, *J* = 2.6 Hz, 1H), 6.68 (q, *J* = 6.3 Hz, 1H), 6.78 (d, *J* = 8.1 Hz, 1H), 7.30 – 7.23 (m, 1H), 7.32 (s, 1H), 7.62 (d, *J* = 7.7 Hz, 1H), 7.75 (d, *J* = 8.4 Hz, 2H), 8.28 – 8.17 (m, 2H), 8.52 (s, 1H); ^{13}C NMR (126 MHz, DMSO-d$_6$) δ = 65.77, 115.02, 115.37, 117.94, 124.04, 127.88, 128.49, 134.03, 147.70, 147.89, 149.78, 163.77 ppm.

2-(2-Hydoxyphenyl)-2,3-dihydroquinazolin-4(1*H*)-one:

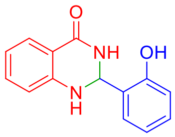

M.P: 222-224 °C; ^{1}H NMR (500 MHz, DMSO-d$_6$) δ = 7.04 – 6.87 (m, 3H), 7.42 (d, J = 7.8 Hz, 1H), 7.52 (t, J = 7.5 Hz, 1H), 7.72 (d, J = 8.2 Hz, 1H), 7.82 (t, J = 7.7 Hz, 1H), 8.18 (dd, J = 40.3, 8.0 Hz, 3H), 12.44 (s, 1H), 13.78 (s, 1H); ^{13}C NMR (126 MHz, DMSO-d$_6$) δ = 114.13, 118.34, 119.24, 121.17, 126.48, 127.35, 128.13, 134.14, 135.41, 146.53, 154.19, 160.57, 161.86 ppm.

2,2'-(1,4-phenylene)bis(2,3-dihydroquinazolin-4(1H)-one):

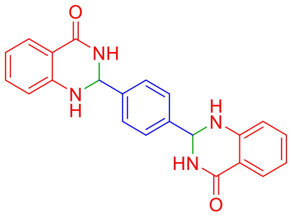

M.P: 242-245 °C; ^{1}H NMR (500 MHz, DMSO-d$_6$) δ = 6.80 – 6.60 (m, 2H), 7.26 (dq, *J* = 24.9, 8.9 Hz, 3H), 7.58 – 7.46 (m, 3H), 7.87 – 7.69 (m, 4H), 8.25 – 8.02 (m, 3H), 8.36 (d, *J* = 8.6 Hz, 1H), 9.01 – 8.60 (m, 1H), 10.16 – 9.85 (m, 1H), ^{13}C NMR (126 MHz, DMSO-d$_6$) δ = 56.54, 121.57, 121.63, 126.37, 127.15, 129.00, 135.14, 148.77, 163.07 ppm

2-(4-Hydroxy)-2, 3-dihydroquinazolin-4(1*H*)-one:

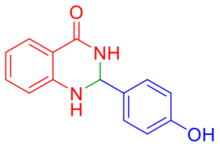

M.P: 272-275 °C; ^{1}H NMR (500 MHz, DMSO-d_6) δ = 6.91 (d, *J* = 8.3 Hz, 3H), 7.44 (t, *J* = 7.5 Hz, 1H), 7.67 (d, *J* = 8.2 Hz, 1H), 7.77 (t, *J* = 7.7 Hz, 1H), 8.11 (dd, *J* = 12.0, 8.1 Hz, 4H), 10.16 (s, 1H), 12.45 – 12.07 (m, 1H); ^{13}C NMR (126 MHz, DMSO-d_6) δ = 113.36, 115.83, 121.05, 123.70, 126.28, 126.33, 127.65, 130.06, 134.90, 149.52, 152.60, 161.03, 162.81 ppm.

2-(4-Methoxyphenyl)-2, 3-dihydroquinazolin-4(1*H*)-one:

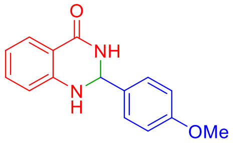

M.P: 183-185°C; ^{1}H NMR (400 MHz, DMSO-d_6): *δ* = 3.75 (s, 3H), 5.71 (t, *J* = 1.5 Hz, 1H), 6.68 (td, *J* = 7.4, 1.1 Hz, 1H), 6.74 (dd, *J* = 8.2, 1.0 Hz, 1H), 6.97 – 6.89 (m, 2H), 7.02 (s, 1H), 7.30 – 7.20 (m, 1H), 7.46 – 7.38 (m, 2H), 7.61 (dd, *J* = 7.7, 1.7 Hz, 1H), 8.20 (d, *J* = 2.2 Hz, 1H).

2-(4-Tolyl)-2, 3-dihydroquinazolin-4(1*H*)-one:

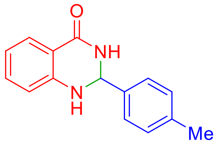

M.P: 219-220 °C; ^{1}H NMR (400 MHz, DMSO-d_6): *δ* = 2.30 (s, 3H), 5.71 (t, J = 1.8 Hz, 1H), 6.67 (td, J = 7.5, 1.1 Hz, 1H), 6.74 (dd, J = 8.1, 1.1 Hz, 1H), 7.06 (s, 1H), 7.28 – 7.16 (m, 3H), 7.41 – 7.34 (m, 2H), 7.61 (dd, J = 7.8, 1.6 Hz, 1H), 8.24 (t, J = 1.9 Hz, 1H) ppm.

2-(pyridin-3-yl)-2,3-dihydroquinazolin-4(1H)-one:

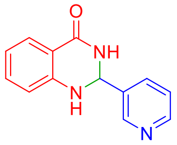

M.P: 218-220 °C; ^{1}H NMR (400 MHz, DMSO-d_6): δ = 5.86 (s, 1H), 6.79 – 6.66 (m, 2H), 7.17 (s, 1H), 7.30 – 7.19 (m, 1H), 7.43 (ddd, J = 7.7, 5.0, 2.2 Hz, 1H), 7.63 (d, J = 7.8 Hz, 1H), 7.93 – 7.83 (m, 1H), 8.38 (s, 1H), 8.55 (dd, J = 4.7, 2.1 Hz, 1H), 8.68 (d, J = 2.5 Hz, 1H); ^{13}C NMR (126 MHz, DMSO-d_6) δ = 65.15, 115.03, 115.50, 117.98, 124.01, 127.87, 133.94, 135.13, 137.29, 148.17, 148.84, 150.13, 164.05 ppm.

2-(3,4-dimethoxyphenyl)-2,3-dihydroquinazolin-4(1*H*)-one:

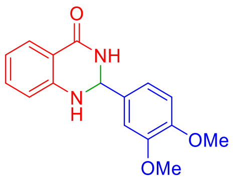

M.P: 210-212 °C; ^{1}H NMR (400 MHz, DMSO-d_6): δ = 3.01 (d, J = 1.9 Hz, 6H), 6.71 – 6.56 (m, 1H), 6.82 – 6.75 (m, 2H), 7.15 – 6.86 (m, 2H), 7.42 (t, J = 7.6 Hz, 1H), 7.67 – 7.50 (m, 2H), 7.77 (t, J = 7.6 Hz, 1H), 8.21 – 8.04 (m, 3H) ppm.

2-(4-(dimethylamino)phenyl)-2,3-dihydroquinazolin-4(1*H*)-one:

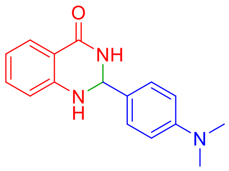

M.P: 206 - 208 °C; ^{1}H NMR (400 MHz, DMSO-d_6): δ = 3.01 (d, J = 1.9 Hz, 6H), 6.71 – 6.56 (m, 1H), 6.82 – 6.75 (m, 2H), 7.15 – 6.86 (m, 2H), 7.42 (t, J = 7.6 Hz, 1H), 7.67 – 7.50 (m, 2H), 7.77 (t, J = 7.6 Hz, 1H), 8.21 – 8.04 (m, 3H); ^{13}C NMR (126 MHz, DMSO-d_6) δ = 40.79, 111.69, 113.28, 119.21, 120.81, 125.86, 126.27, 129.20, 129.34, 129.85, 134.87, 152.75 ppm.

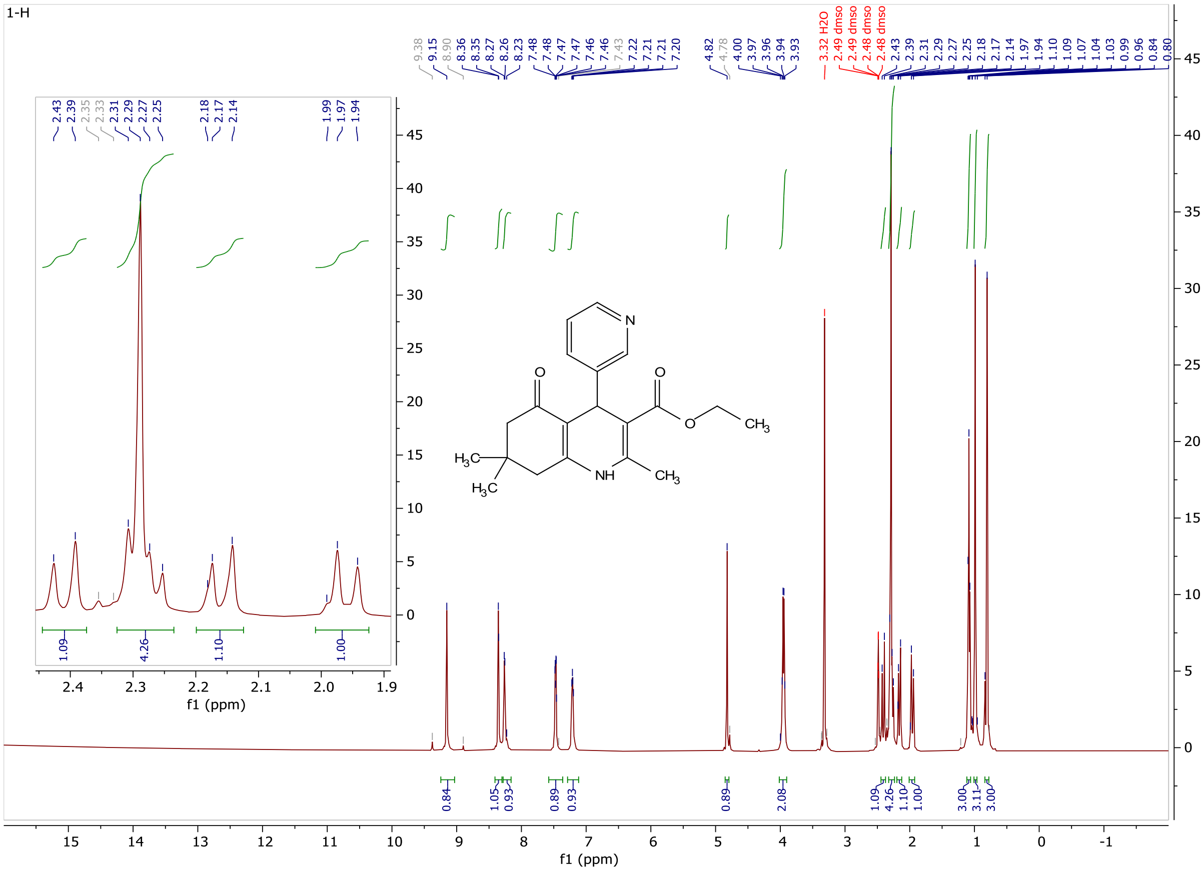

Fig S1. ^{1}H NMR spectrum of Ethyl 4-(pyridin-3-yl)-2,7,7-trimethyl-5-oxo-1,4,5,6,7,8- hexayidroquinolin-3-carboxylate.

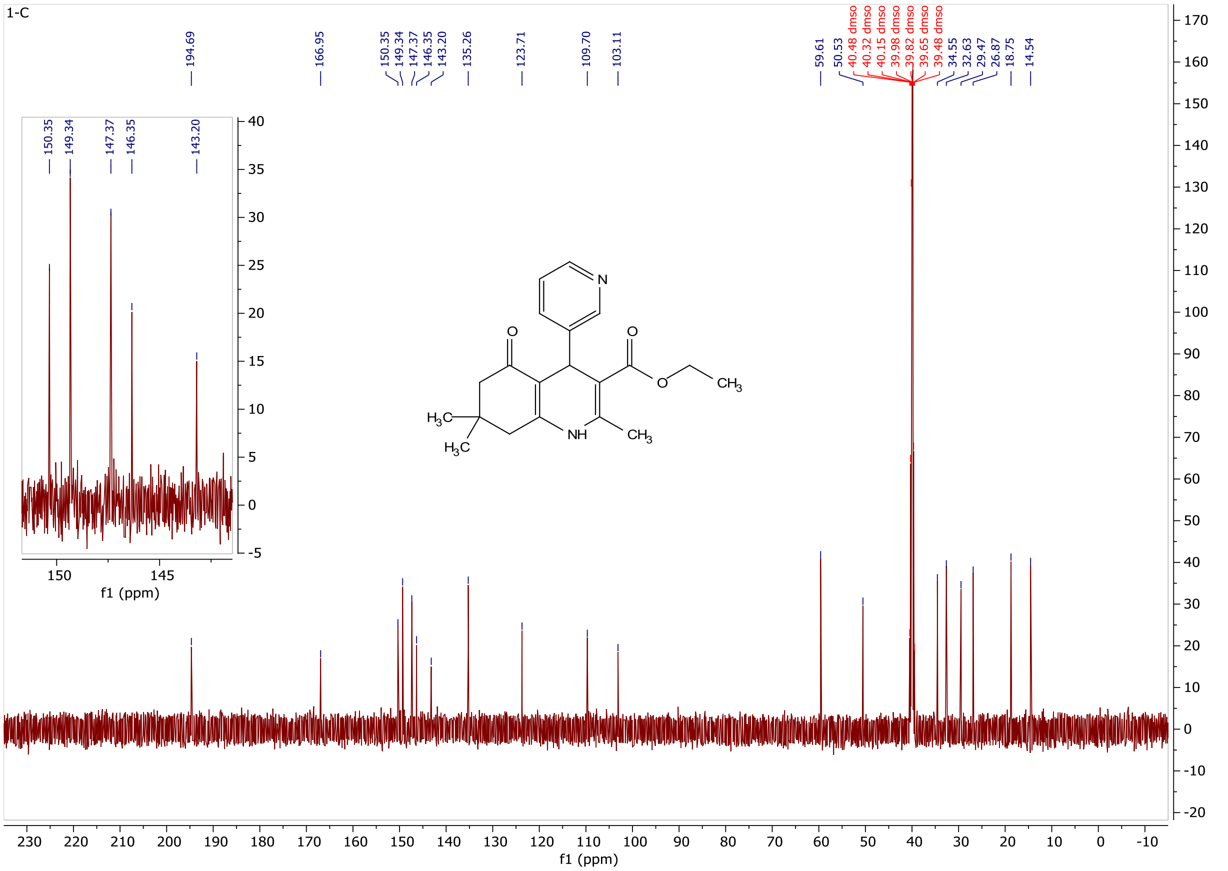

Fig S2. ^{13}C NMR spectrum of Ethyl 4-(pyridin-3-yl)-2,7,7-trimethyl-5-oxo-1,4,5,6,7,8-hexayidroquinolin-3-carboxylate.

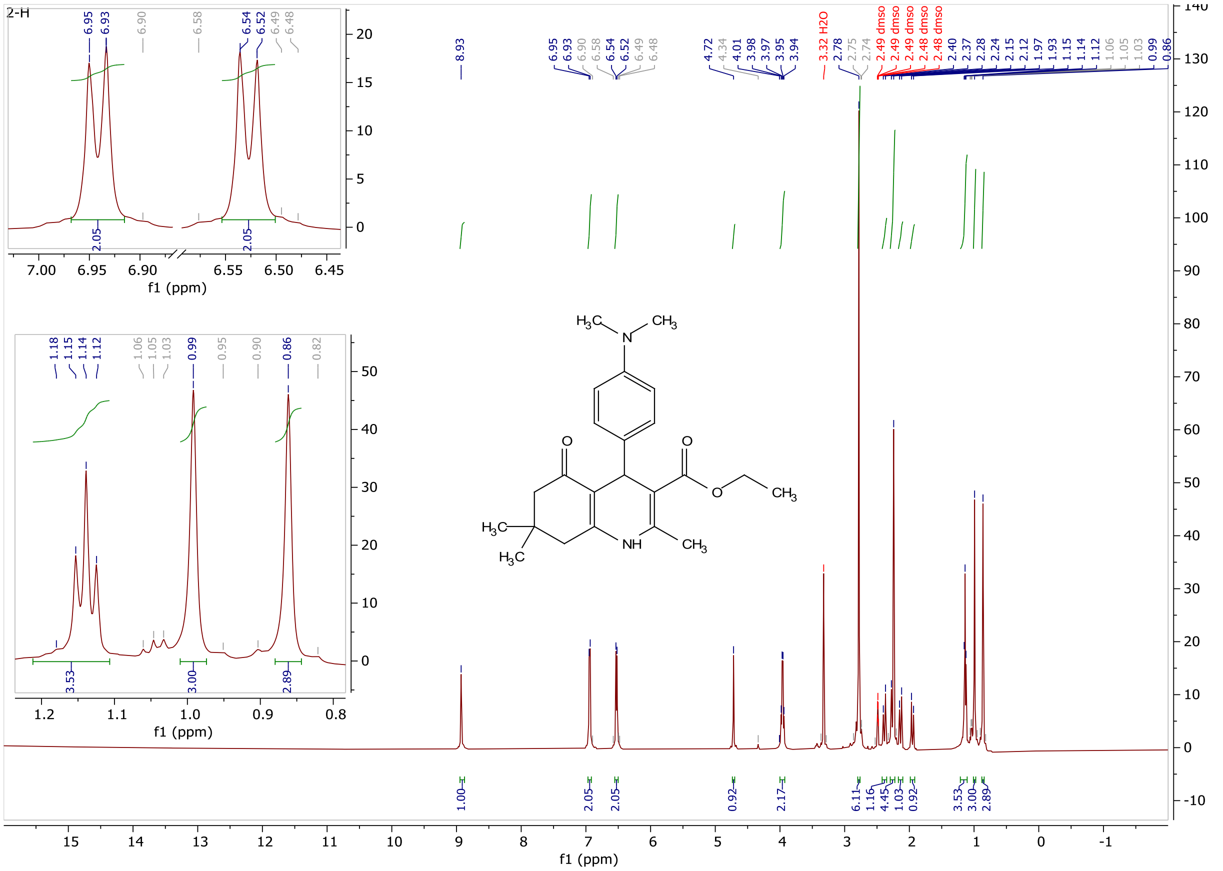

Fig S3. ^{1}H NMR spectrum of Ethyl 4-(4-dimethylamino)phenyl)-2,7,7-trimethyl-5-oxo-1,4,5,6,7,8-hexayidroquinolin-3-carboxylate.

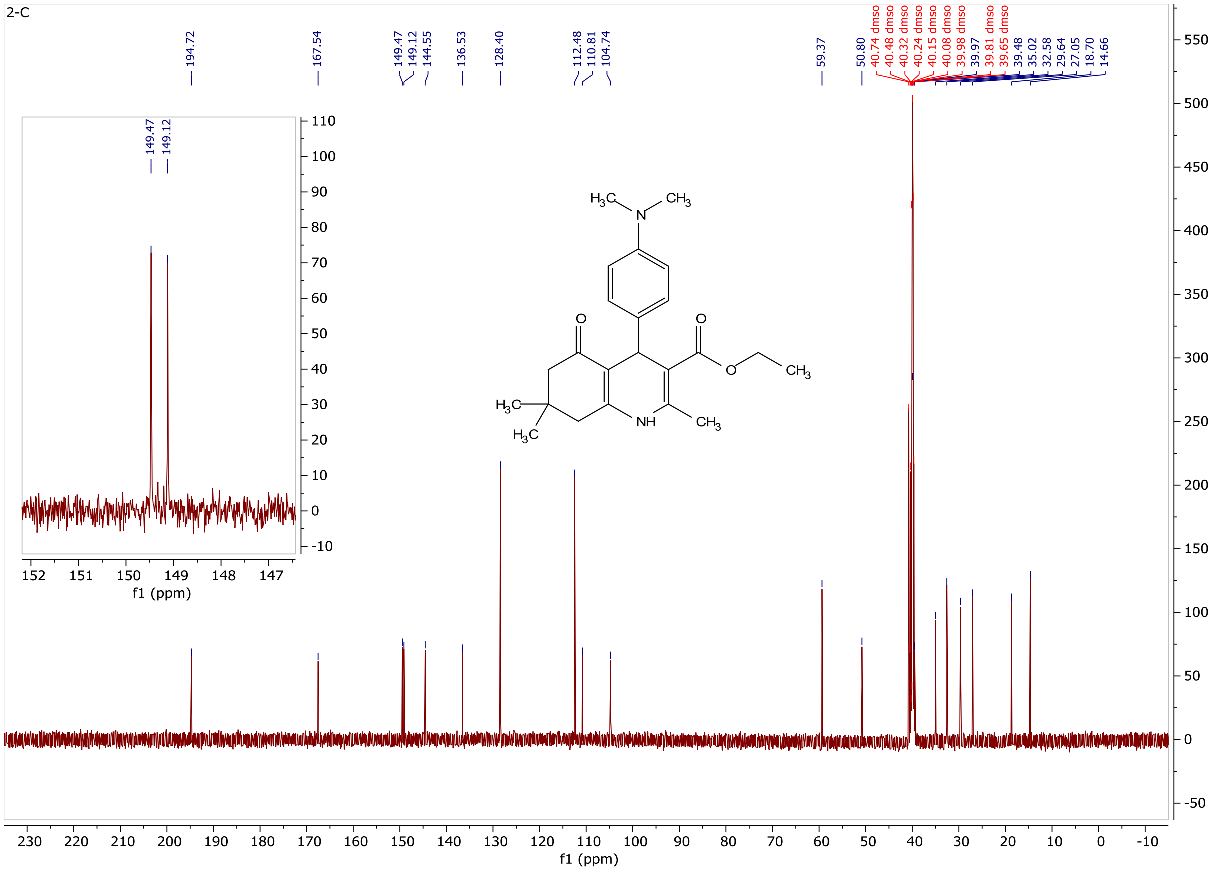

Fig S4. ^{13}C NMR spectrum of Ethyl 4-(4-dimethylamino)phenyl)-2,7,7-trimethyl-5-oxo-1,4,5,6,7,8-hexayidroquinolin-3-carboxylate.

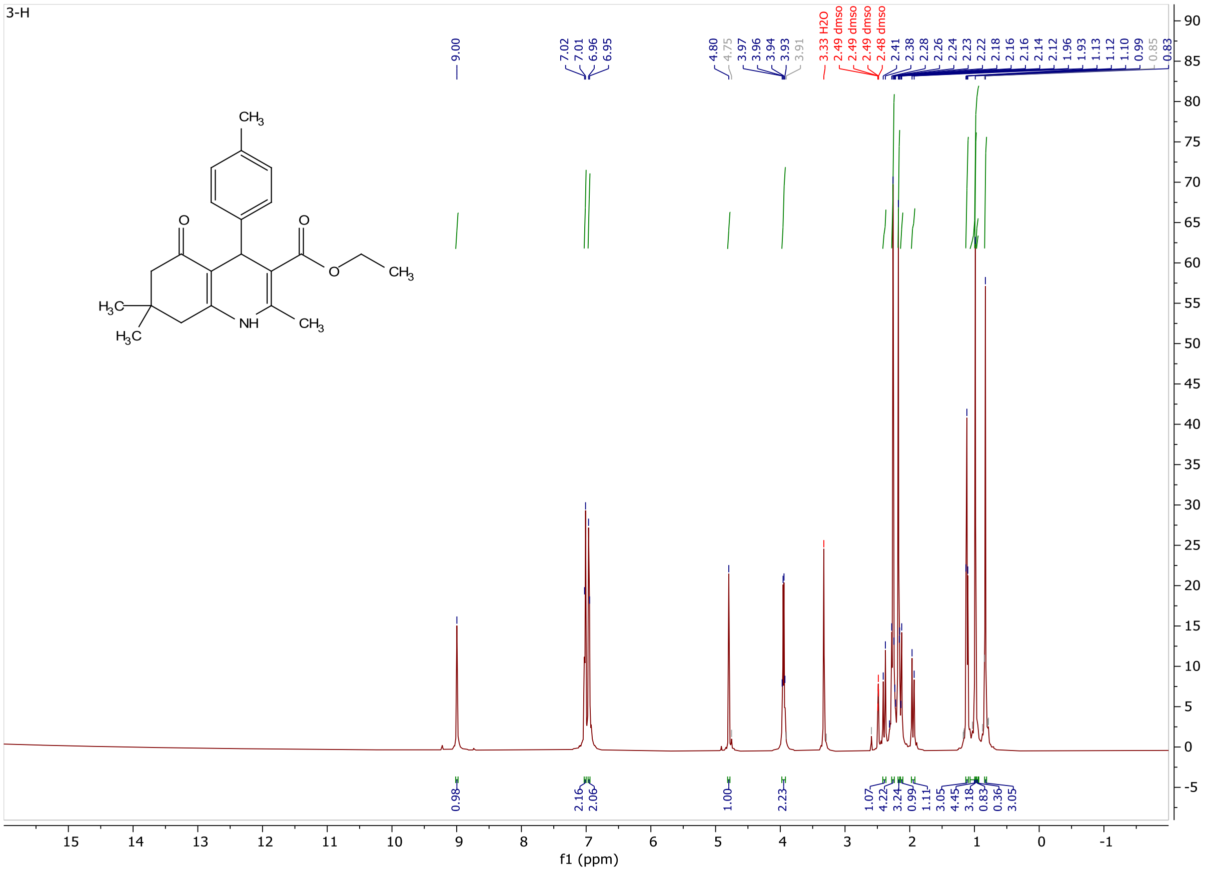

Fig S5. ^{1}H NMR spectrum of Ethyl 2,7,7-trimethyl-5-oxo-4-(p-tolyl)-1,4,5,6,7,8-hexahydroquinoline-3-carboxylate.

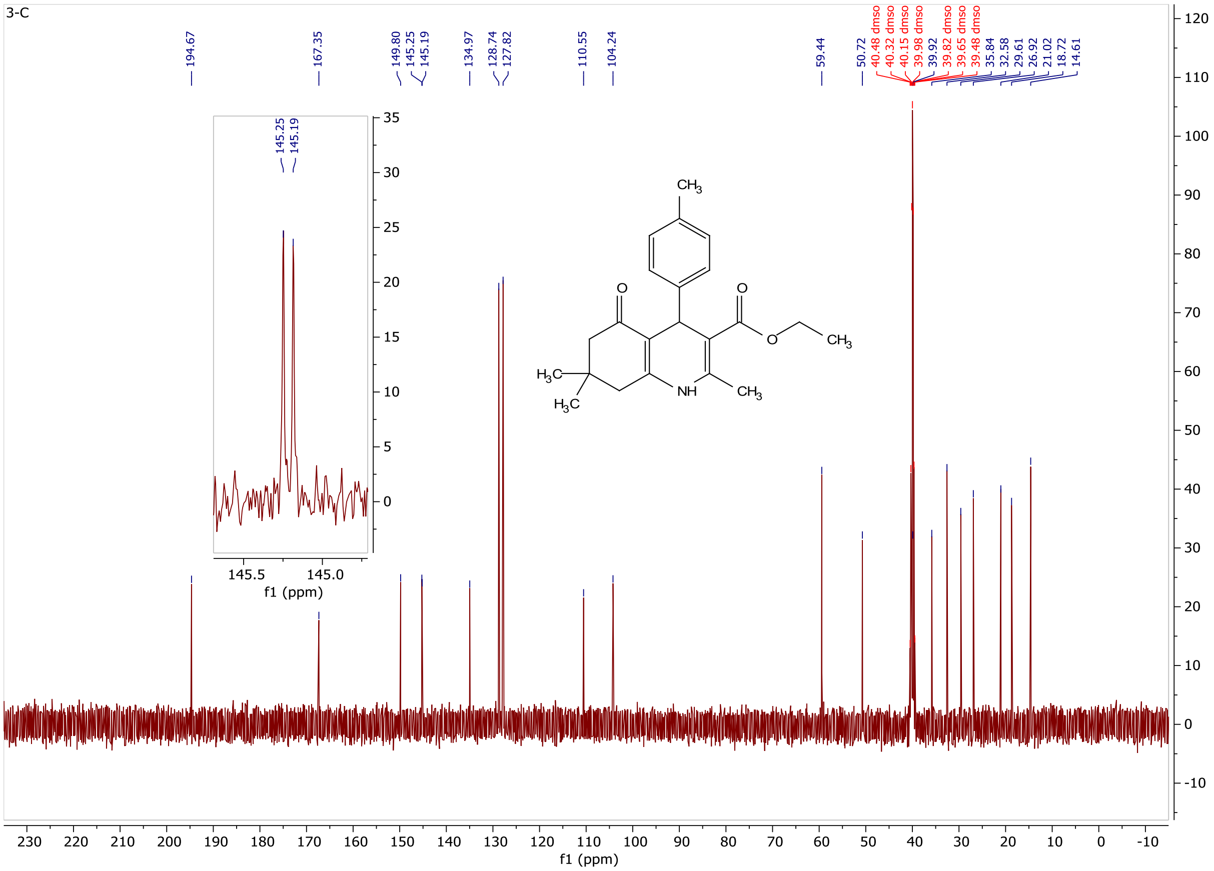

Fig S6. ^{13}C NMR spectrum of Ethyl 2,7,7-trimethyl-5-oxo-4-(p-tolyl)-1,4,5,6,7,8-hexahydroquinoline-3-carboxylate.

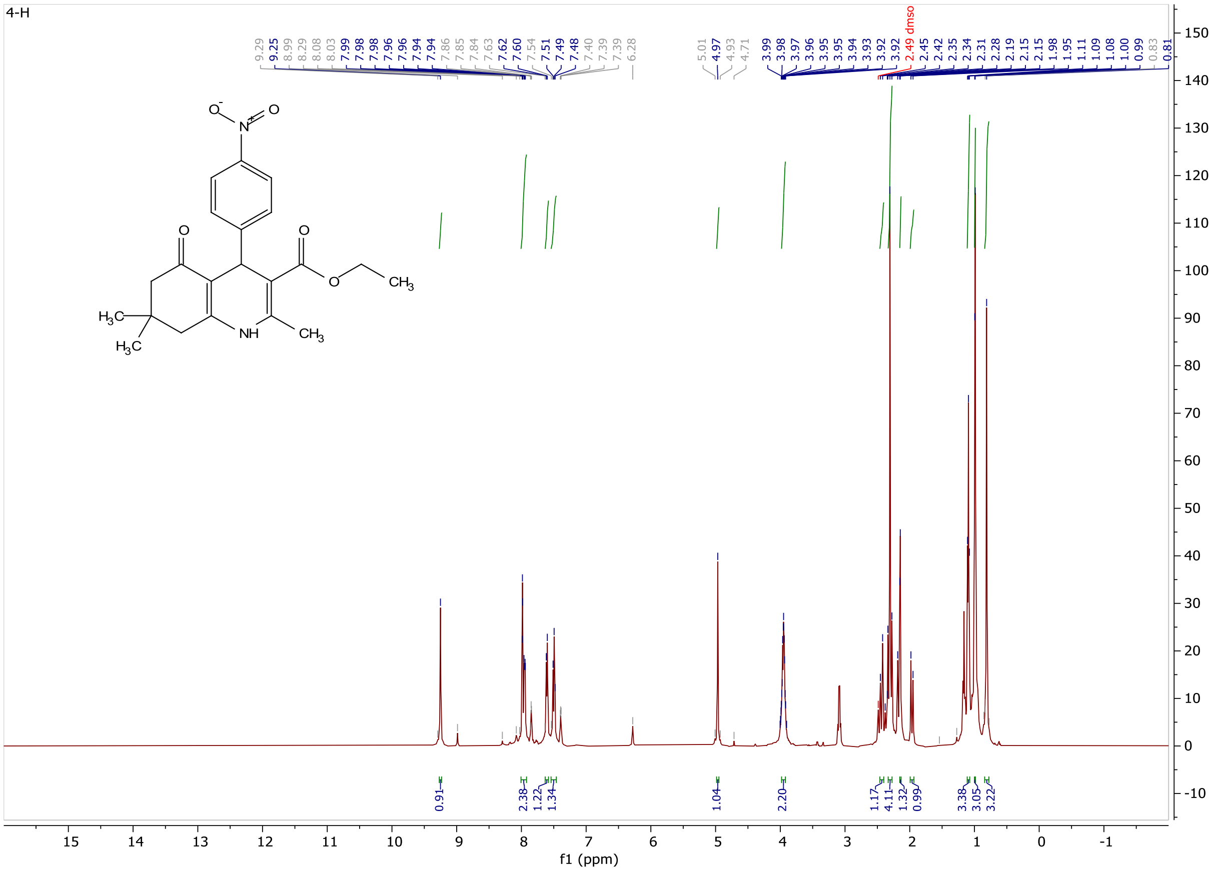

Fig S7. ^{1}H NMR spectrum of 2,7,7-Trimethyl-4-(4-nitro-phenyl)-5-oxo-1,4,4a,5,6,7,8,8a-octahydro-quinoline-3-carboxylic acid ethyl ester.

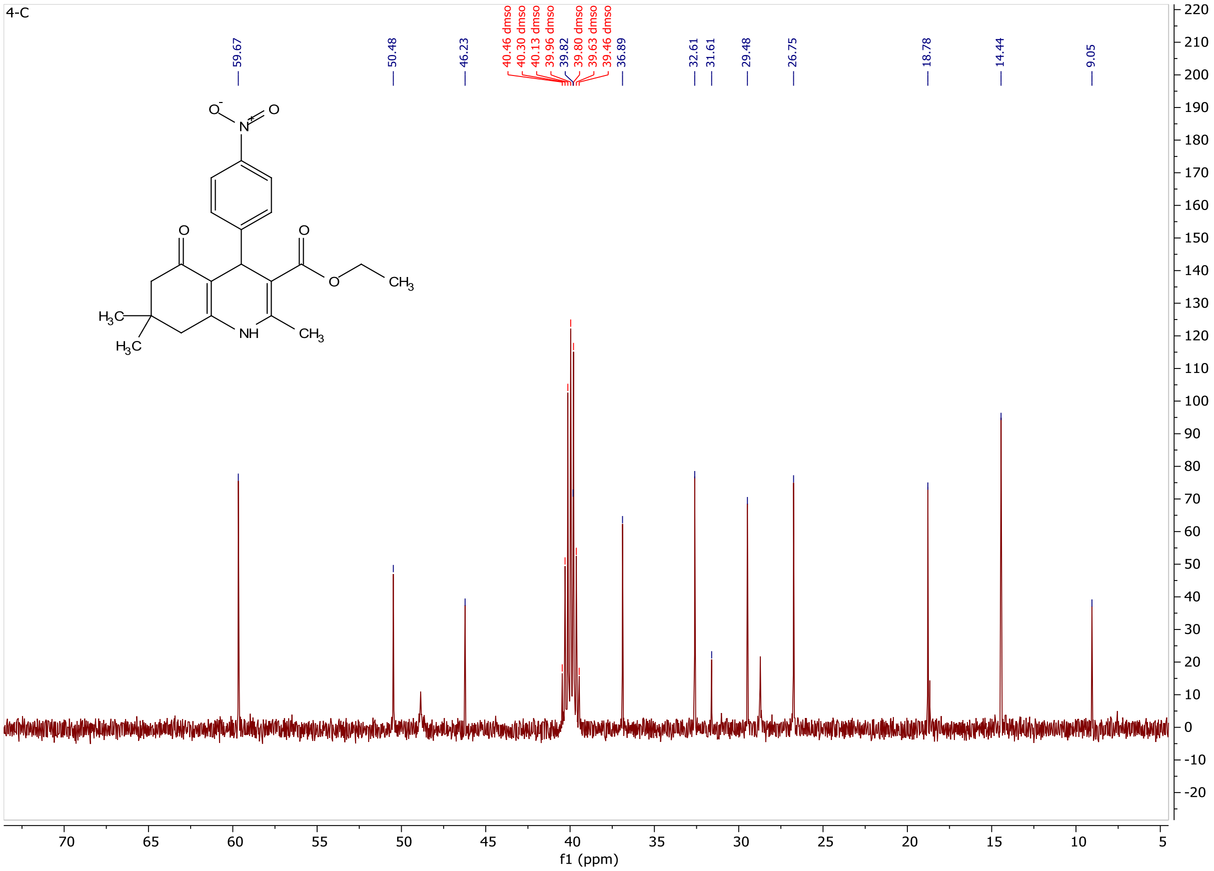

Fig S8. ^{13}C NMR spectrum of 2,7,7-Trimethyl-4-(4-nitro-phenyl)-5-oxo-1,4,4a,5,6,7,8,8a-octahydro-quinoline-3-carboxylic acid ethyl ester.

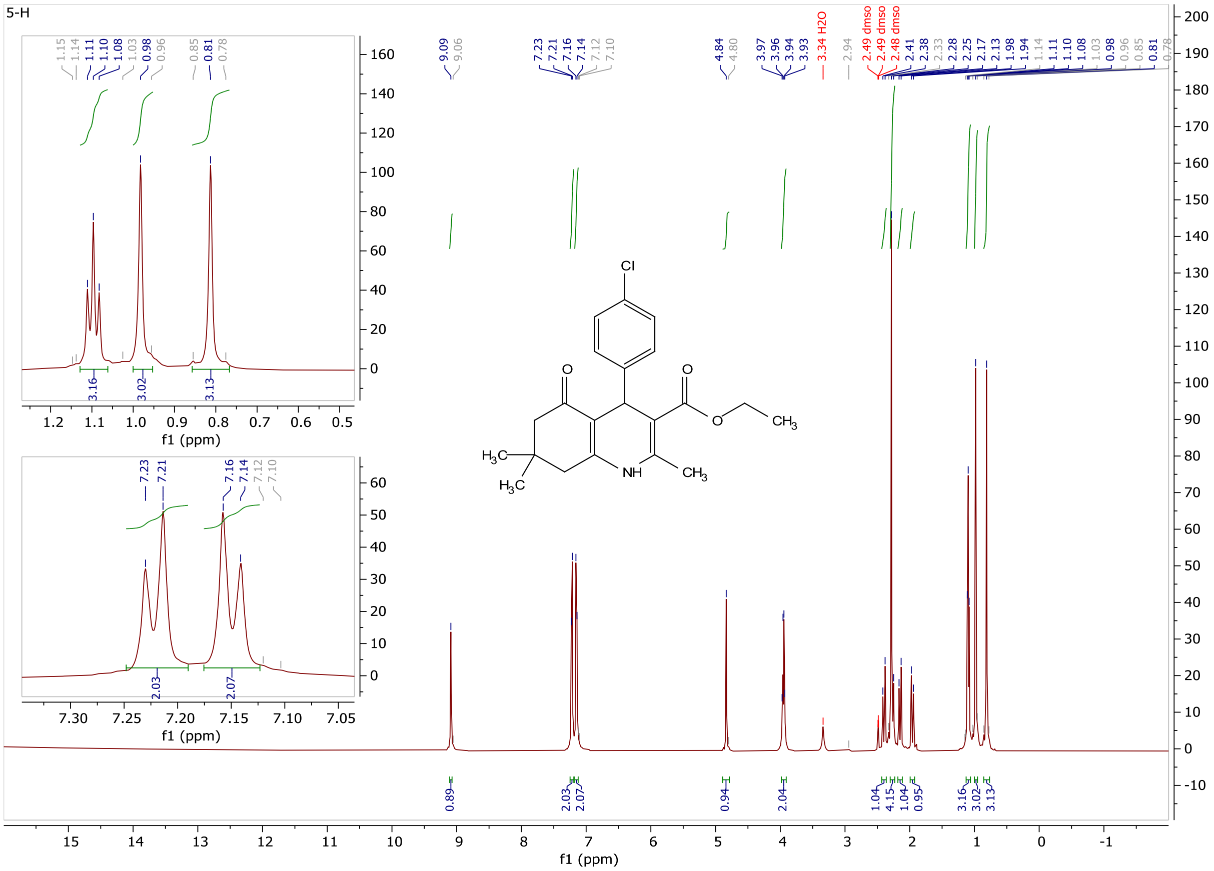

Fig S9. ^{1}H NMR spectrum of 4-(4-Chloro-phenyl)-2,7,7-trimethyl-5-oxo-1,4,4a,5,6,7,8,8aoctahydro-quinoline-3-carboxylic acid ethyl ester

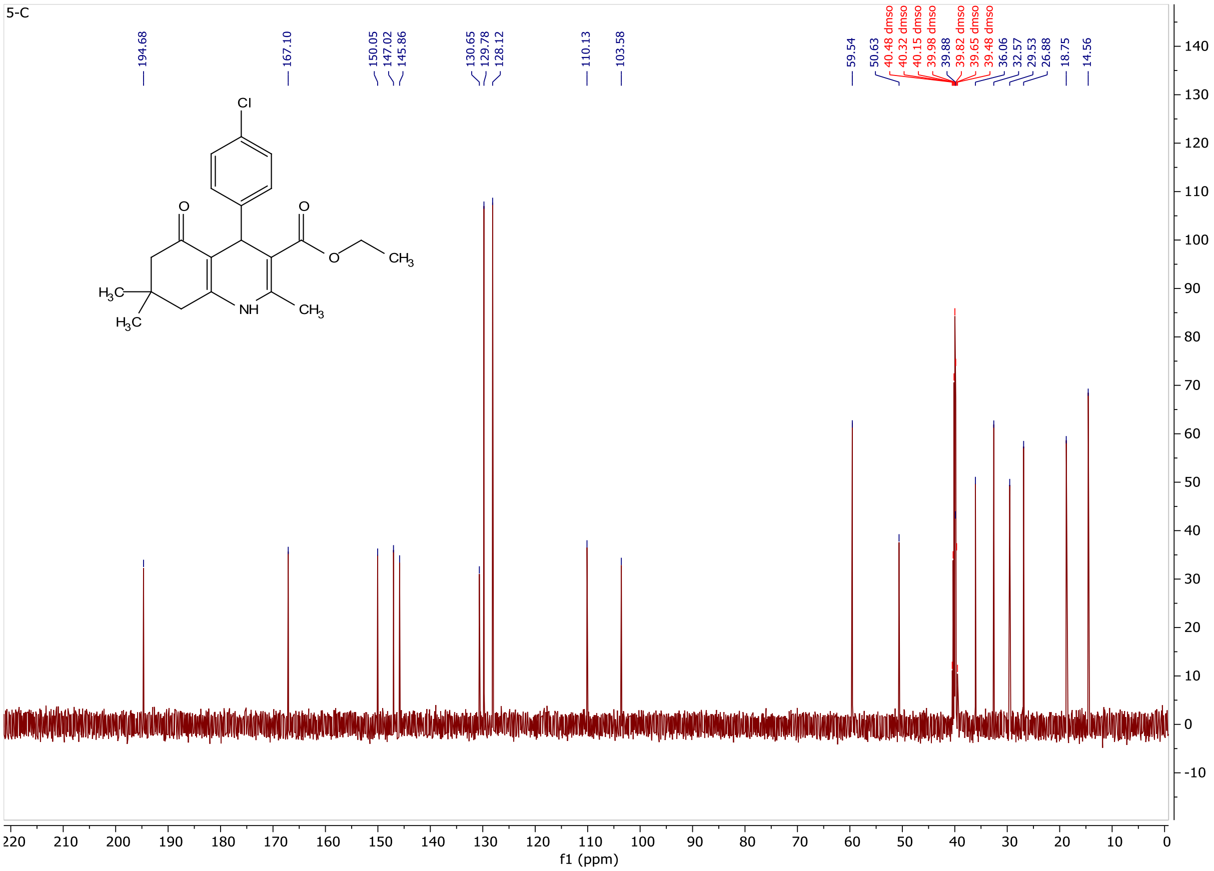

Fig S10. ^{13}C NMR spectrum of 2,7,7-Trimethyl-4-(4-nitro-phenyl)-5-oxo-1,4,4a,5,6,7,8,8a-octahydro-quinoline-3-carboxylic acid ethyl ester.

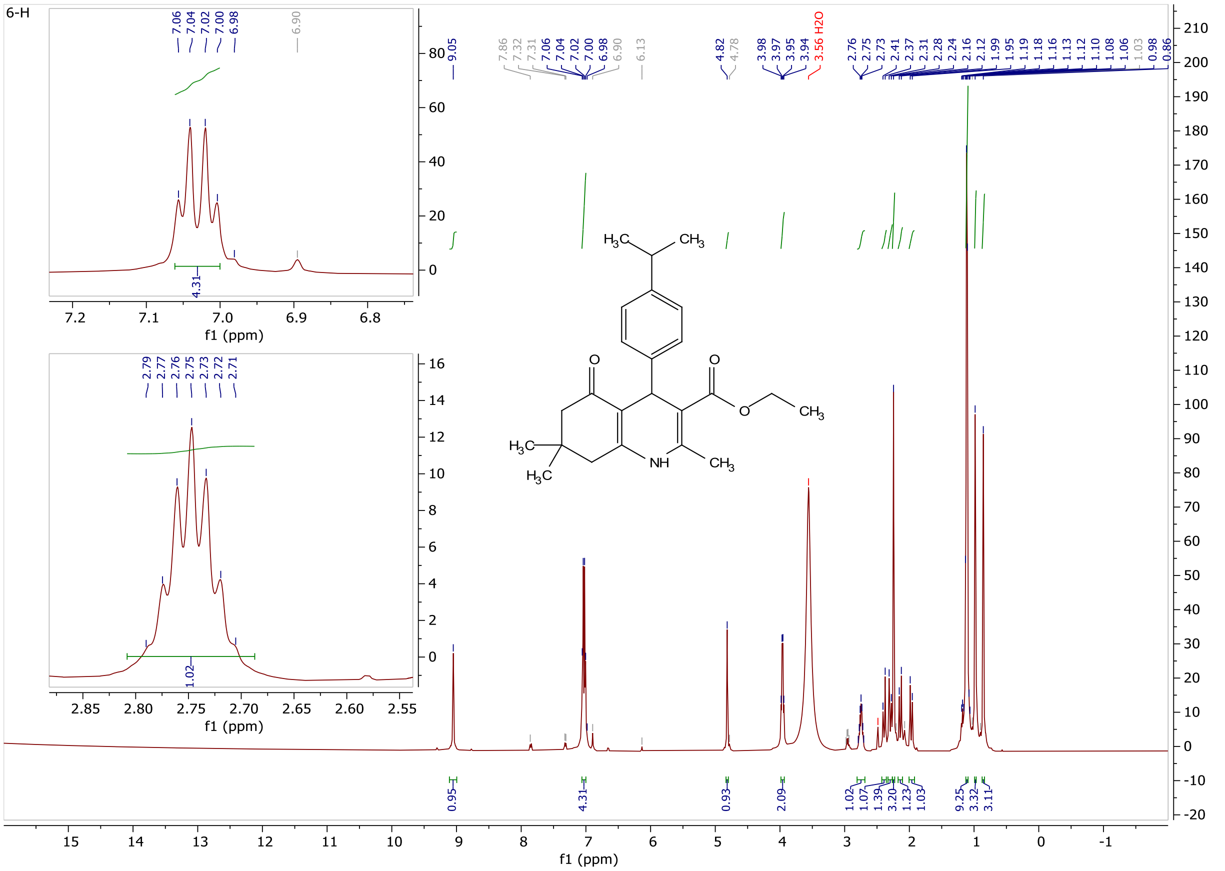

Fig S11. ^{1}H NMR spectrum of Ethyl 1,4,5,6,7,8-hexahydro-4-(4-isopropylphenyl)-2,7,7-trimethyl-5-oxoquinoline-3-carboxylate.

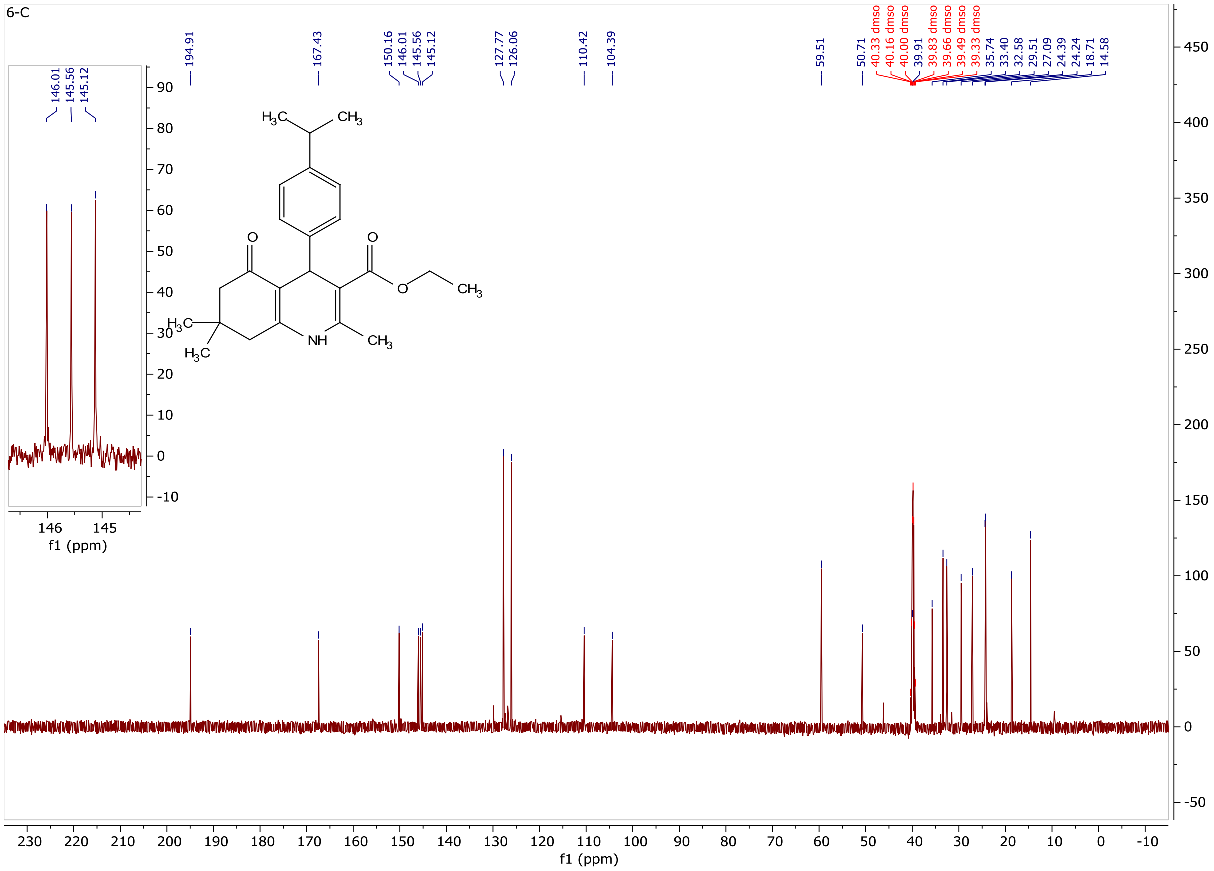

Fig S12. ^{13}C NMR spectrum of Ethyl 1,4,5,6,7,8-hexahydro-4-(4-isopropylphenyl)-2,7,7-trimethyl-5-oxoquinoline-3-carboxylate.

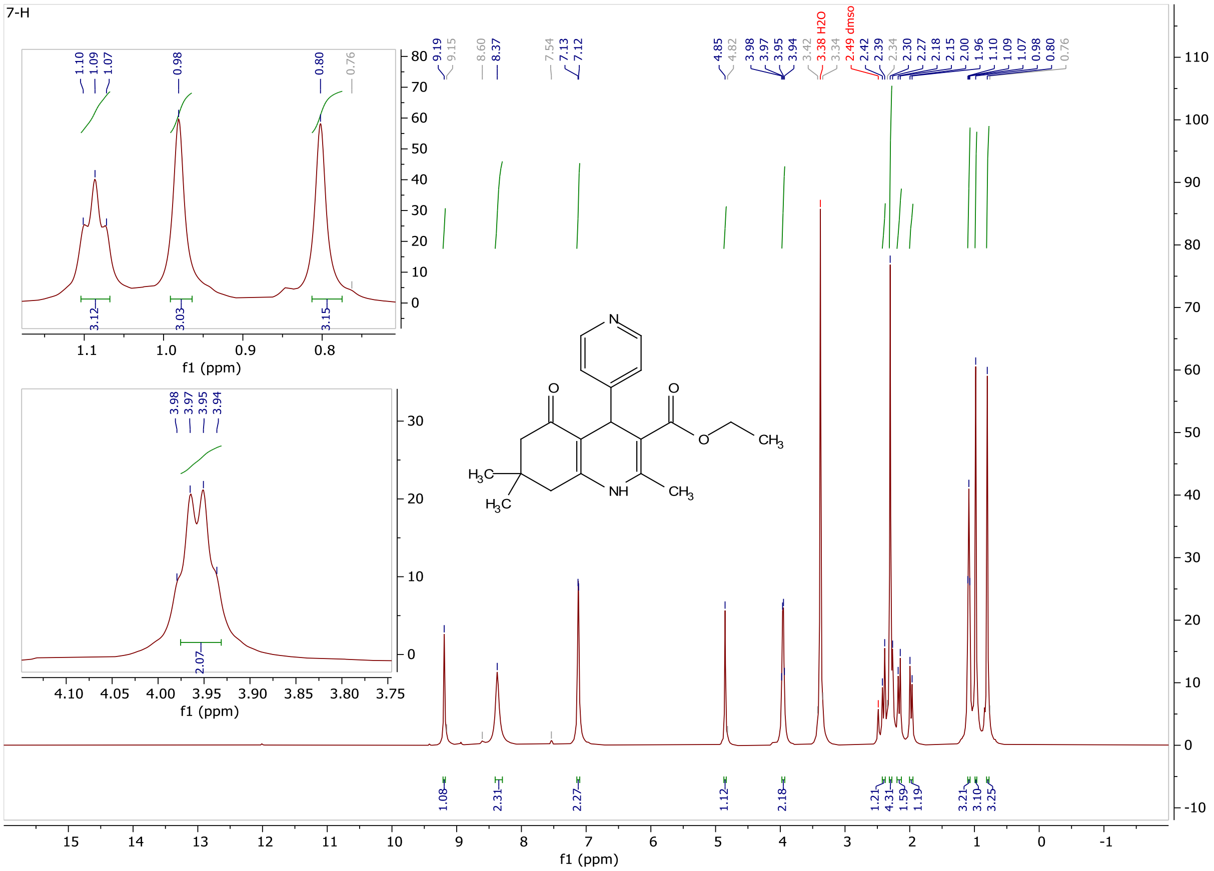

Fig S13. ^{1}H NMR spectrum of Ethyl-2,7,7-trimethyl-5-oxo-4-(pyridin-4-yl)-1,4,5,6,7,8-hexahydroquinoline-3-carboxylate.

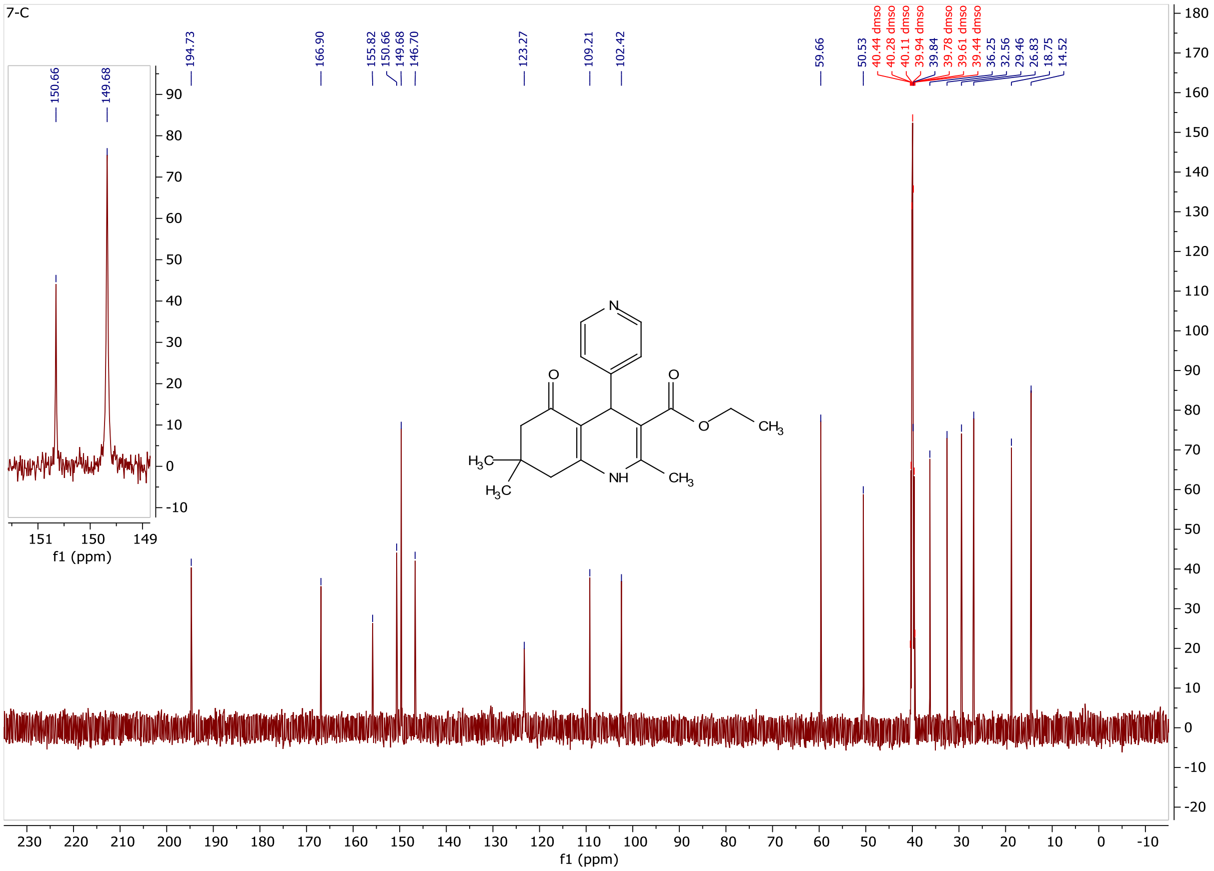

Fig S14. ^{13}C NMR spectrum of Ethyl-2,7,7-trimethyl-5-oxo-4-(pyridin-4-yl)-1,4,5,6,7,8-hexahydroquinoline-3-carboxylate.

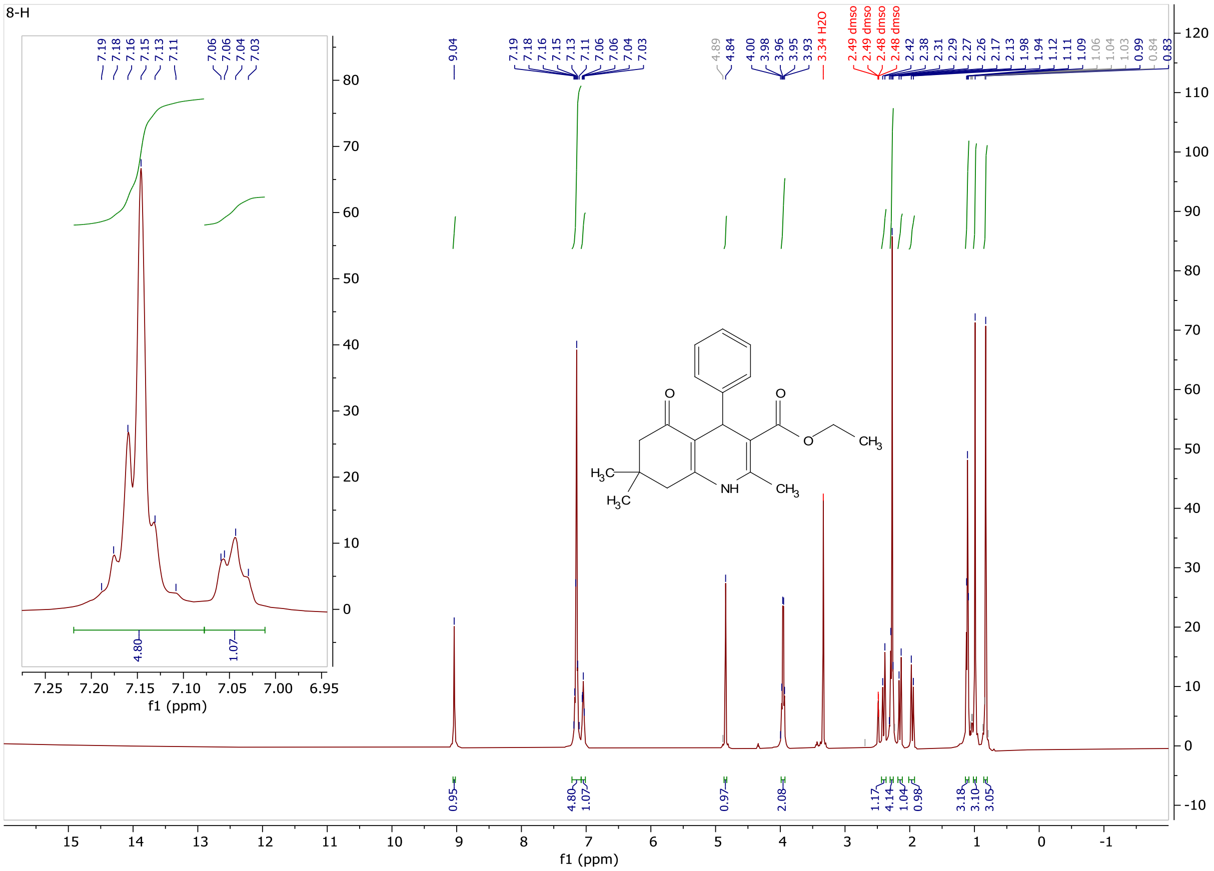

Fig S15. ^{1}H NMR spectrum of Ethyl 2,7,7-trimethyl-5-oxo-4-phenyl-1,4,5,6,7,8-hexahydroquinolin-3-carboxylate.

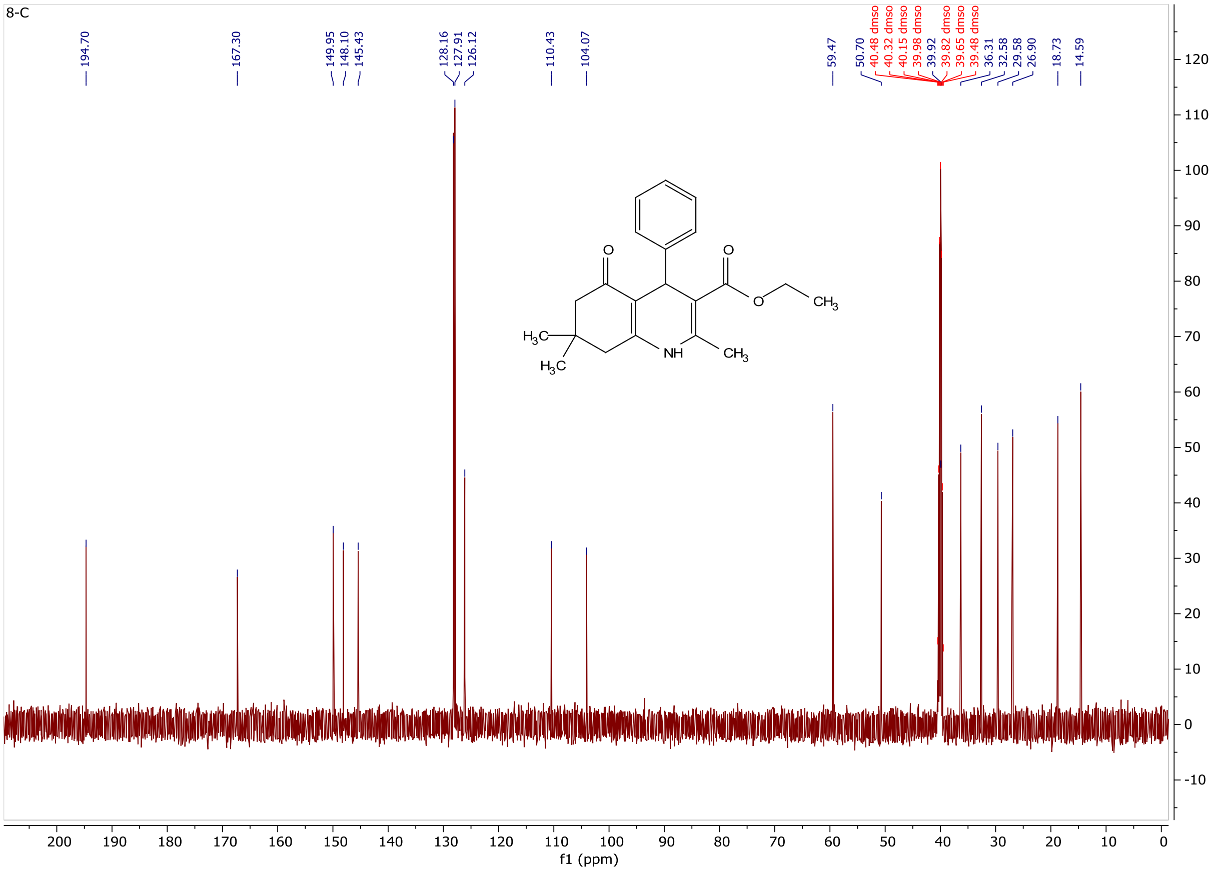

Fig S16. ^{13}C NMR spectrum of Ethyl 2,7,7-trimethyl-5-oxo-4-phenyl-1,4,5,6,7,8-hexahydroquinolin-3-carboxylate.

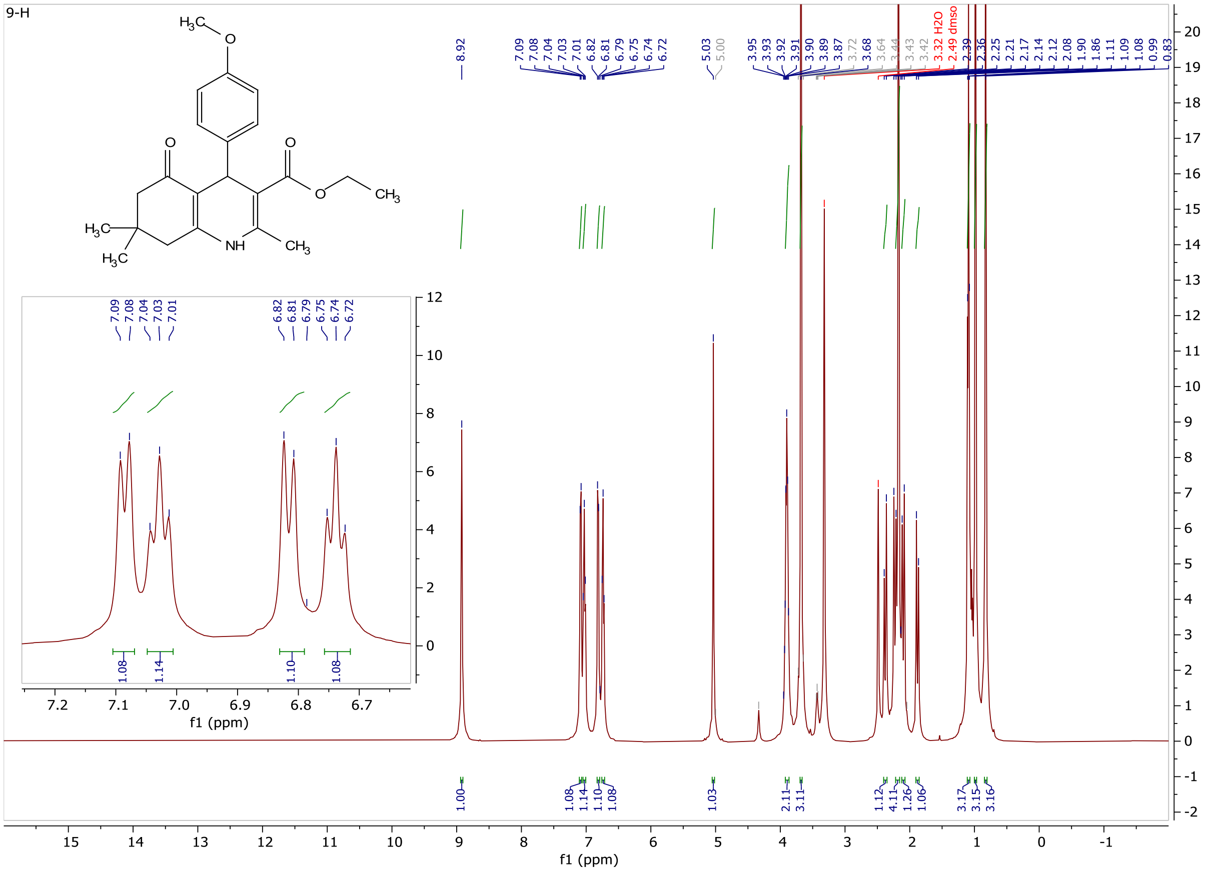

Fig S17. ^{1}H NMR spectrum of Ethyl 4-(4-methoxyphenyl)-2,7,7-trimethyl-5-oxo-1,4,5,6,7,8-hexahydroquinoline-3-carboxylate.

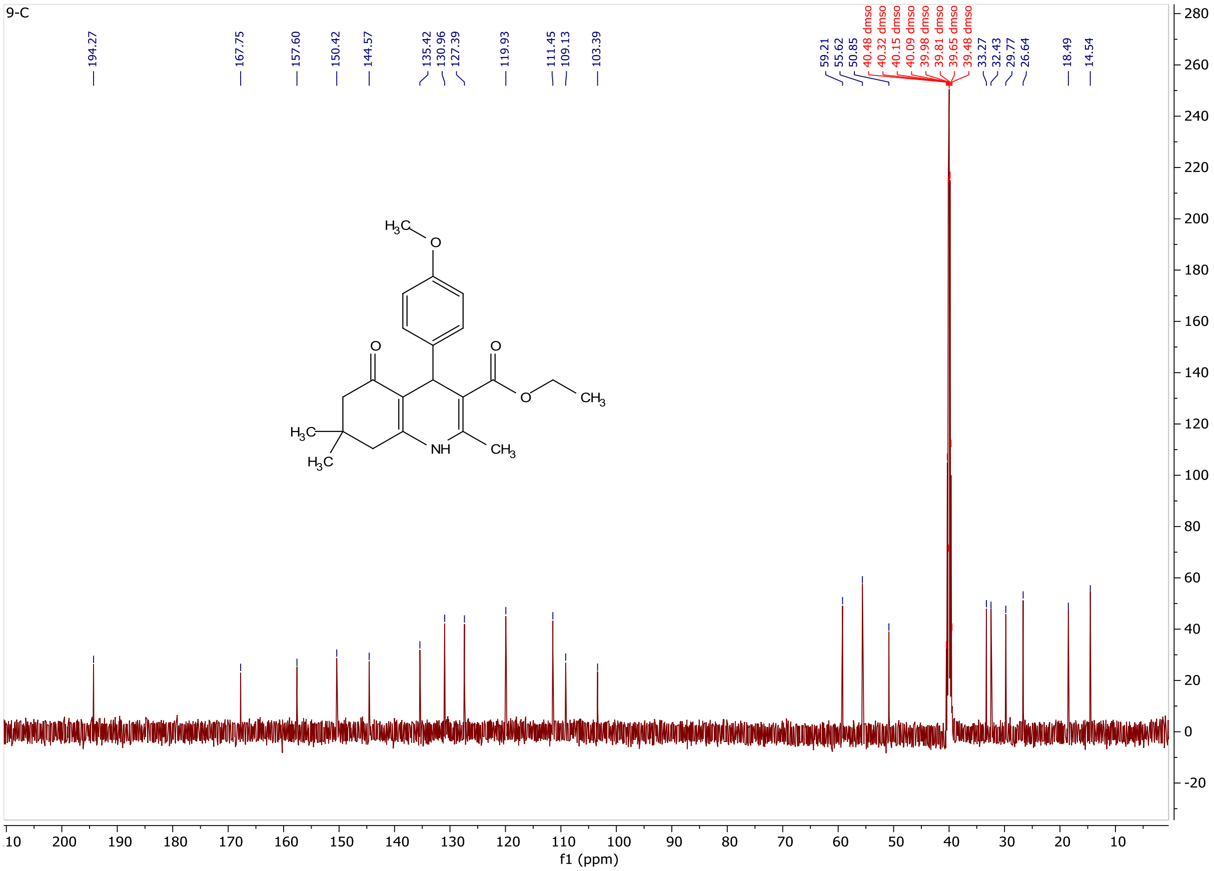

Fig S18. ^{13}C NMR spectrum of Ethyl 4-(4-methoxyphenyl)-2,7,7-trimethyl-5-oxo-1,4,5,6,7,8-hexahydroquinoline-3-carboxylate.

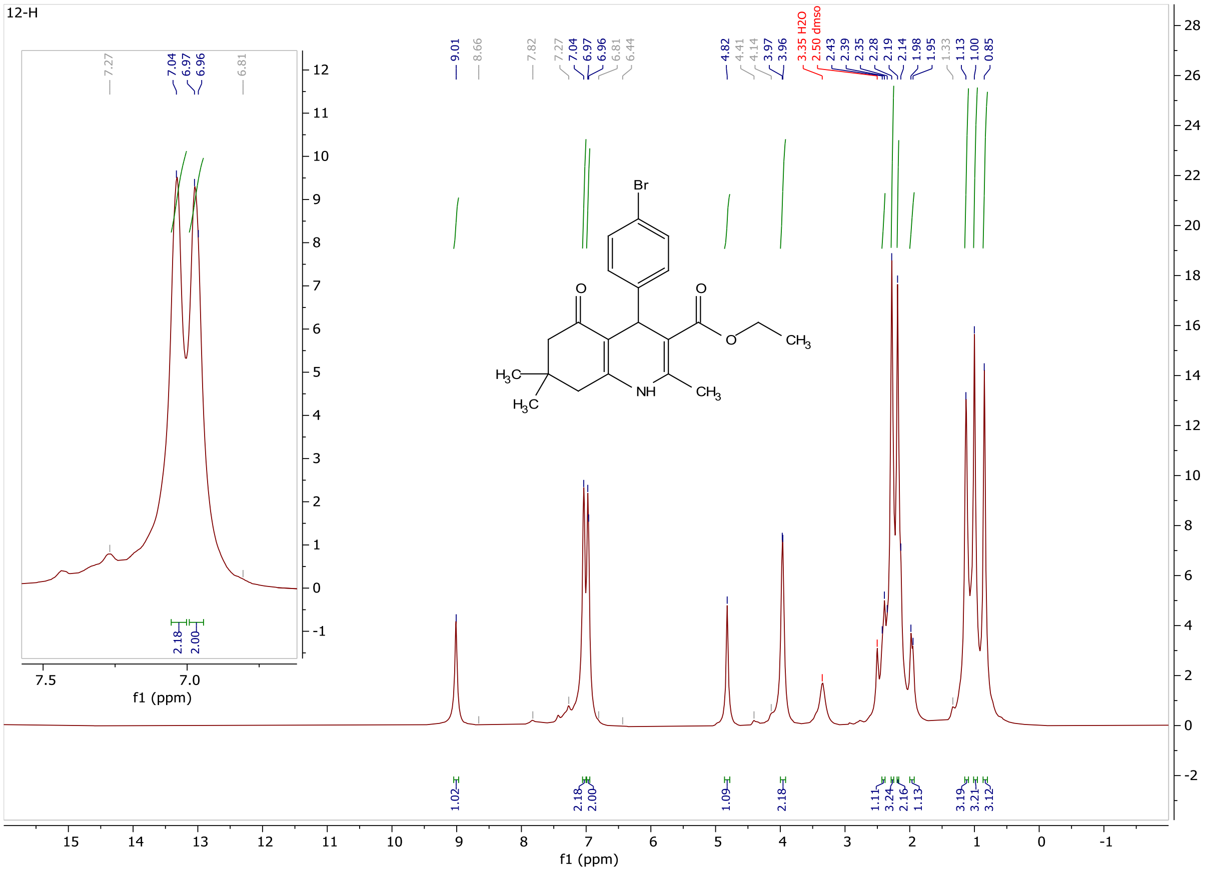

Fig S19. ^{1}H NMR spectrum of Ethyl 4-(4-bromophenyl)-2,7,7-trimethyl-5-oxo-1,4,5,6,7,8-hexahydroquinoline-3 carboxylate.

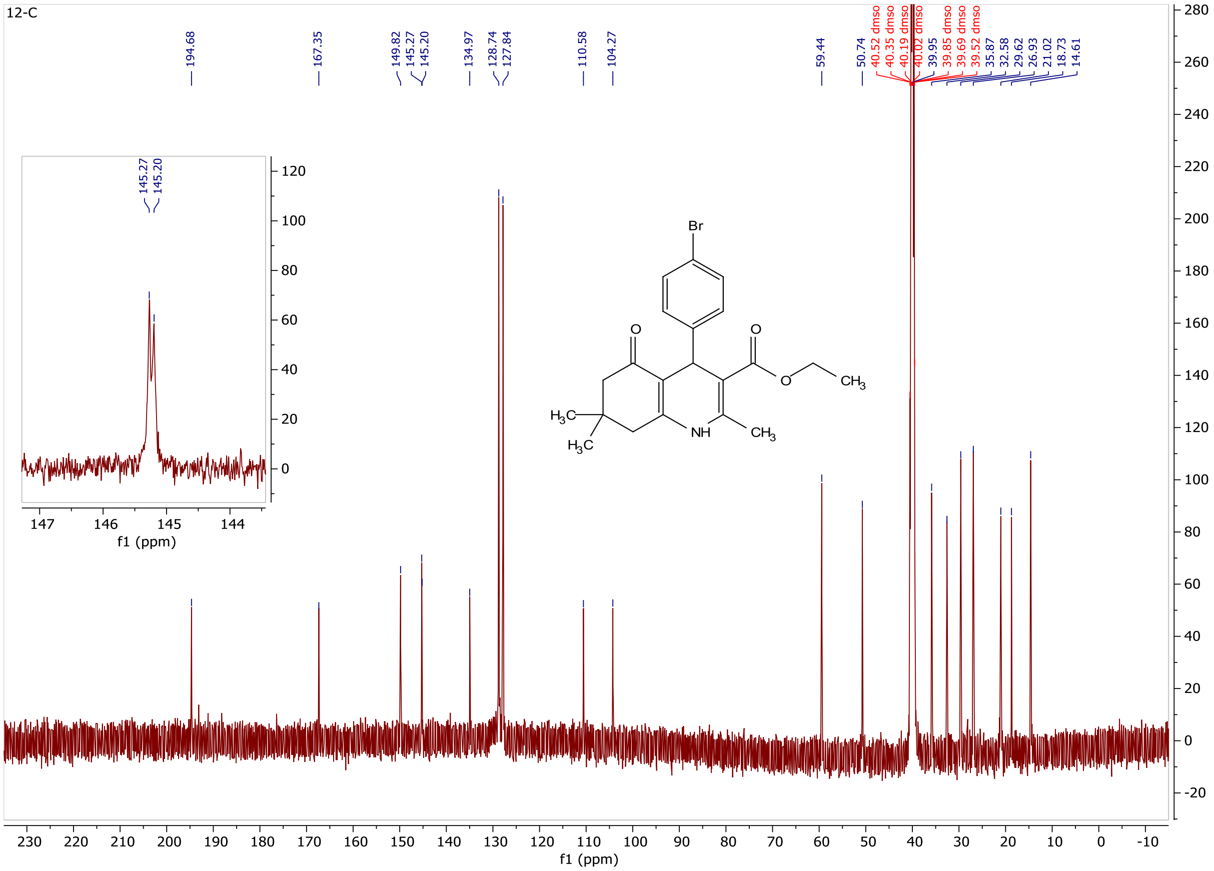

Fig S20. ^{13}C NMR spectrum of Ethyl 4-(4-bromophenyl)-2,7,7-trimethyl-5-oxo-1,4,5,6,7,8-hexahydroquinoline-3 carboxylate.

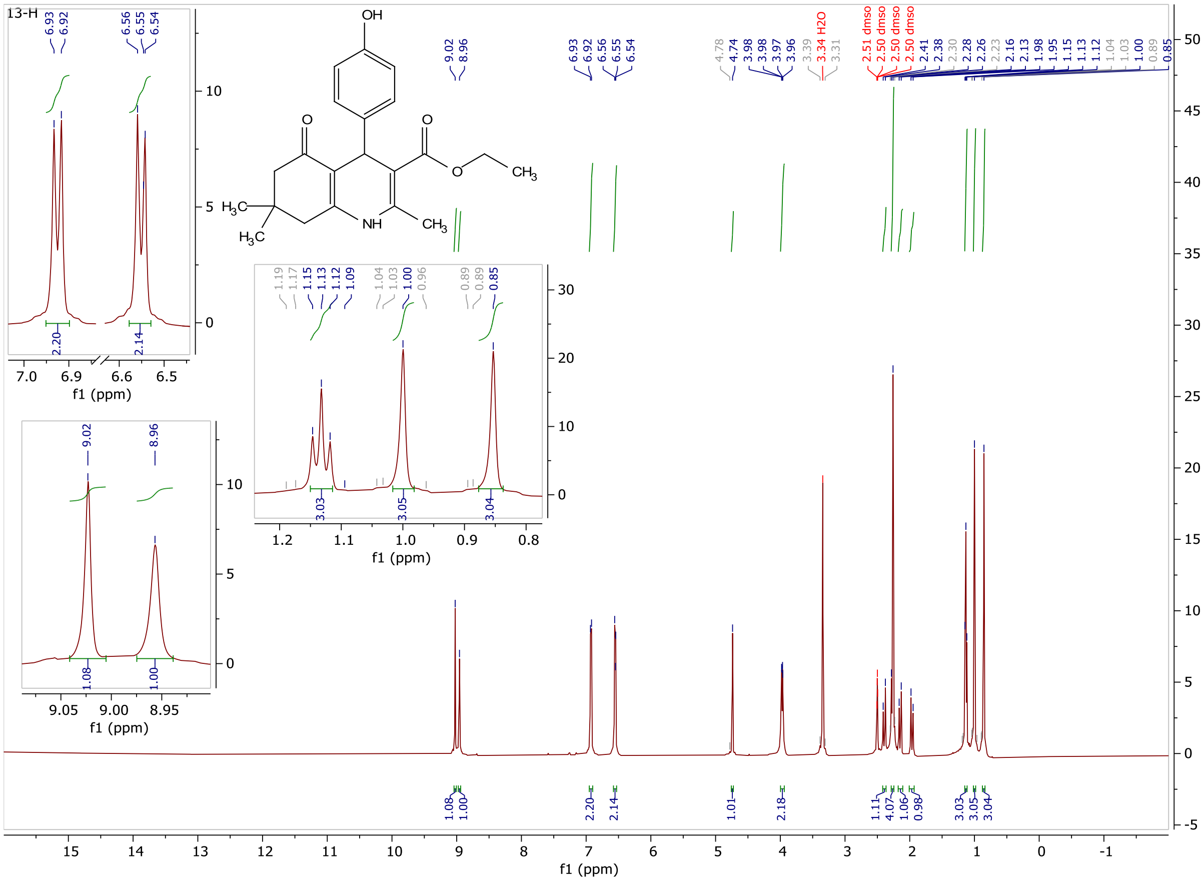

Fig S21. ^{1}H NMR spectrum of Ethyl 2,7,7-trimethyl-5-oxo-4-(4-hydroxy)-1,4,5,6,7,8-hexahydroquinoline-3- carboxylate.

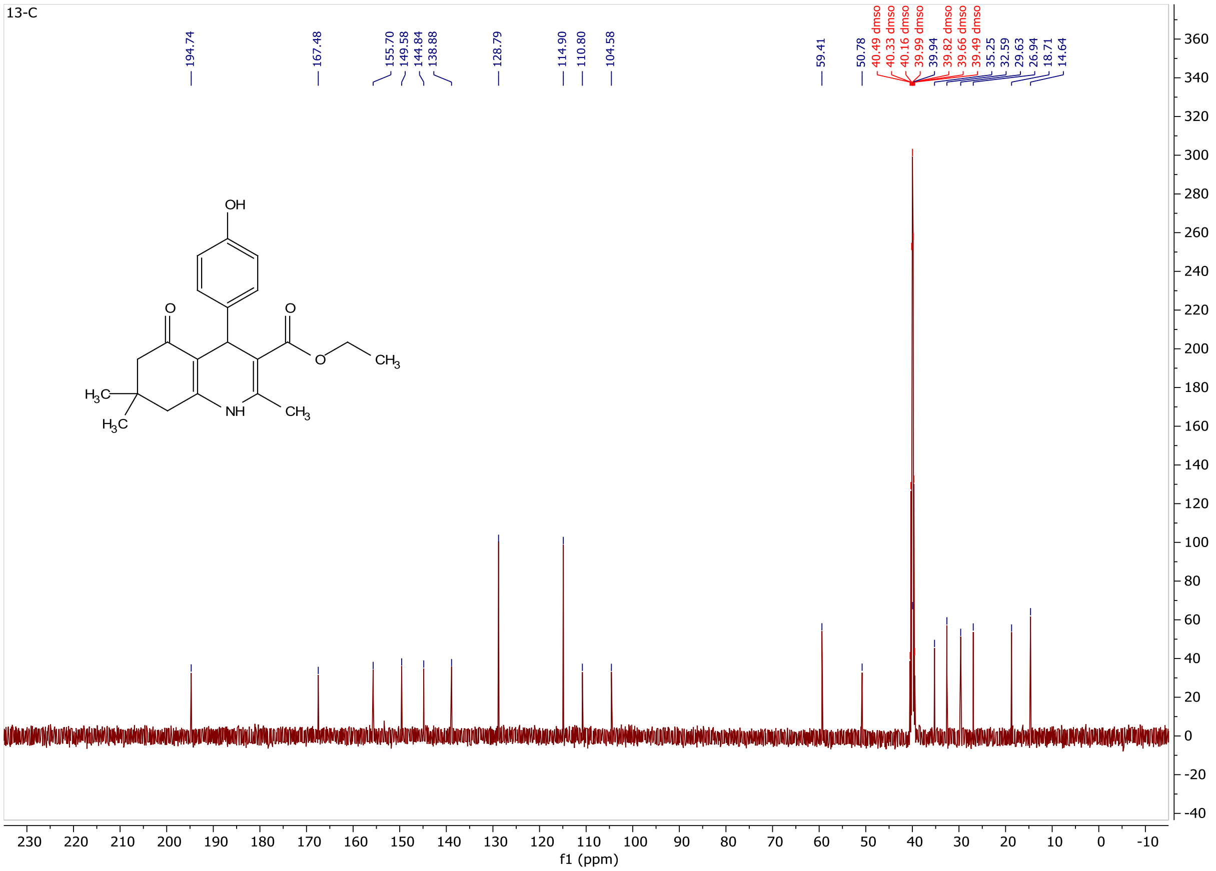

Fig S22. ^{13}C NMR spectrum of Ethyl 2,7,7-trimethyl-5-oxo-4-(4-hydroxy)-1,4,5,6,7,8-hexahydroquinoline-3- carboxylate.

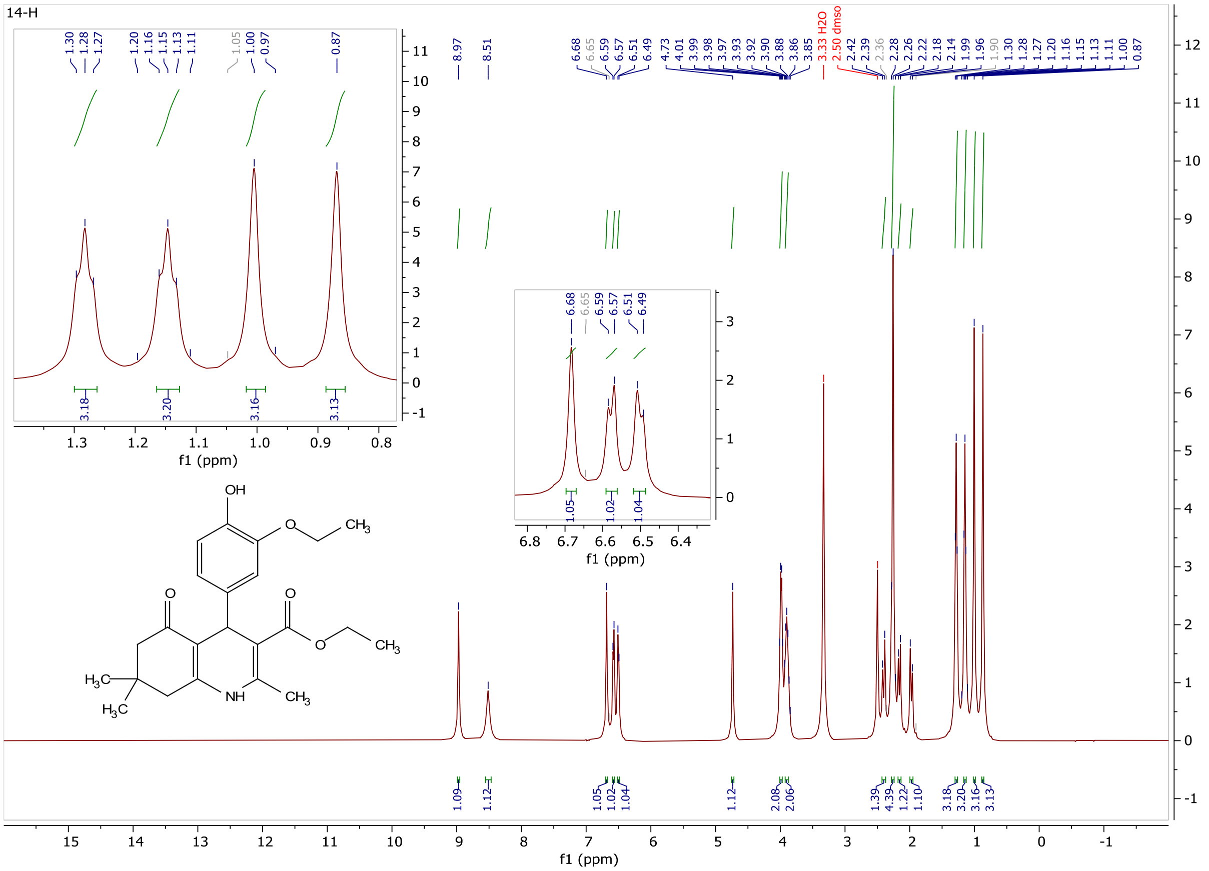

Fig S23. ^{1}H NMR spectrum of Dimethyl 4-(3-ethoxy-4-hydroxyphenyl)-2,6-dimethyl-1,4-dihydropyridine-3,5-dicarboxylate.

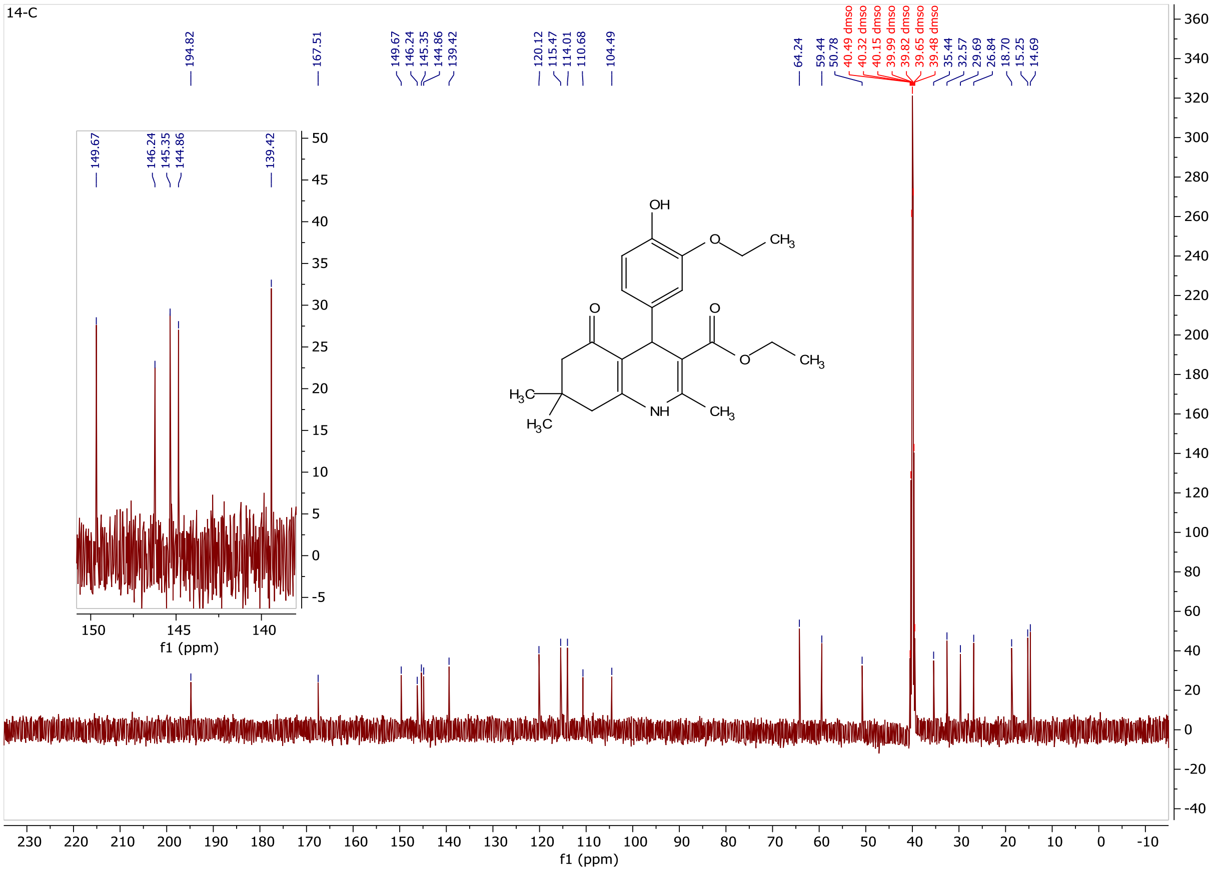

Fig S24. ^{13}C NMR spectrum of Dimethyl 4-(3-ethoxy-4-hydroxyphenyl)-2,6-dimethyl-1,4-dihydropyridine-3,5-dicarboxylate.

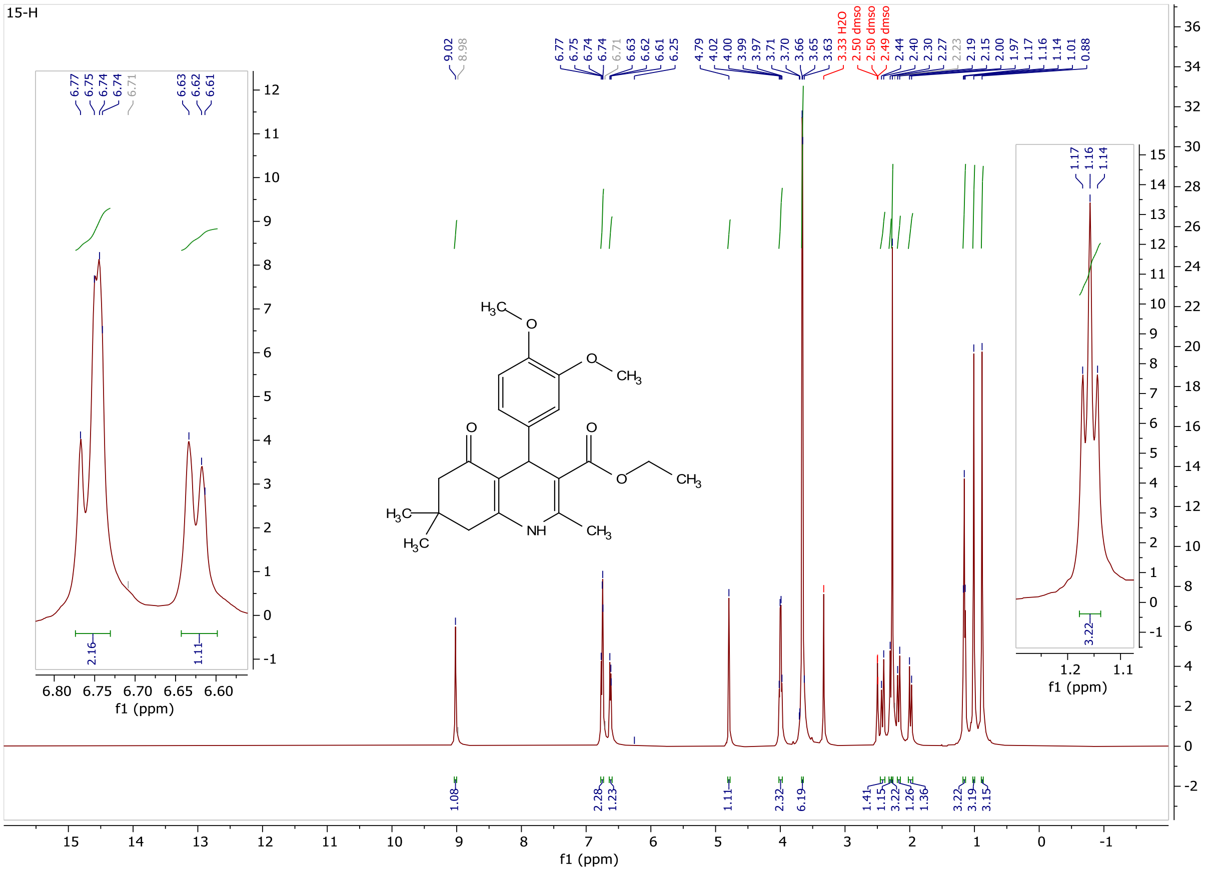

Fig S25. ^{1}H NMR spectrum of Ethyl 4-(3,4-dimethoxyphenyl)-2,7,7-trimethyl-5-oxo-1,4,5,6,7,8-hexahydroquinoline-3-carboxylate.

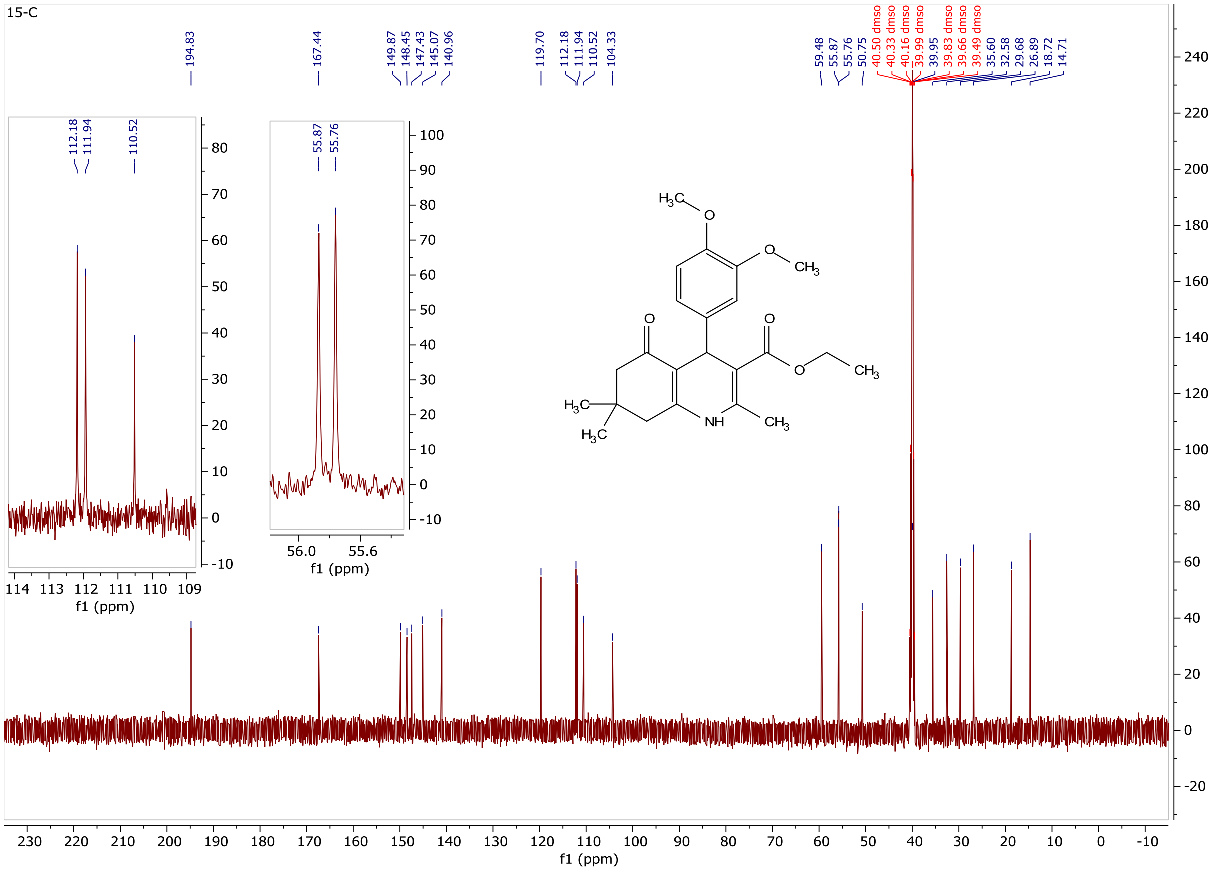

Fig S26. ^{13}C NMR spectrum of Ethyl 4-(3,4-dimethoxyphenyl)-2,7,7-trimethyl-5-oxo-1,4,5,6,7,8-hexahydroquinoline-3-carboxylate.

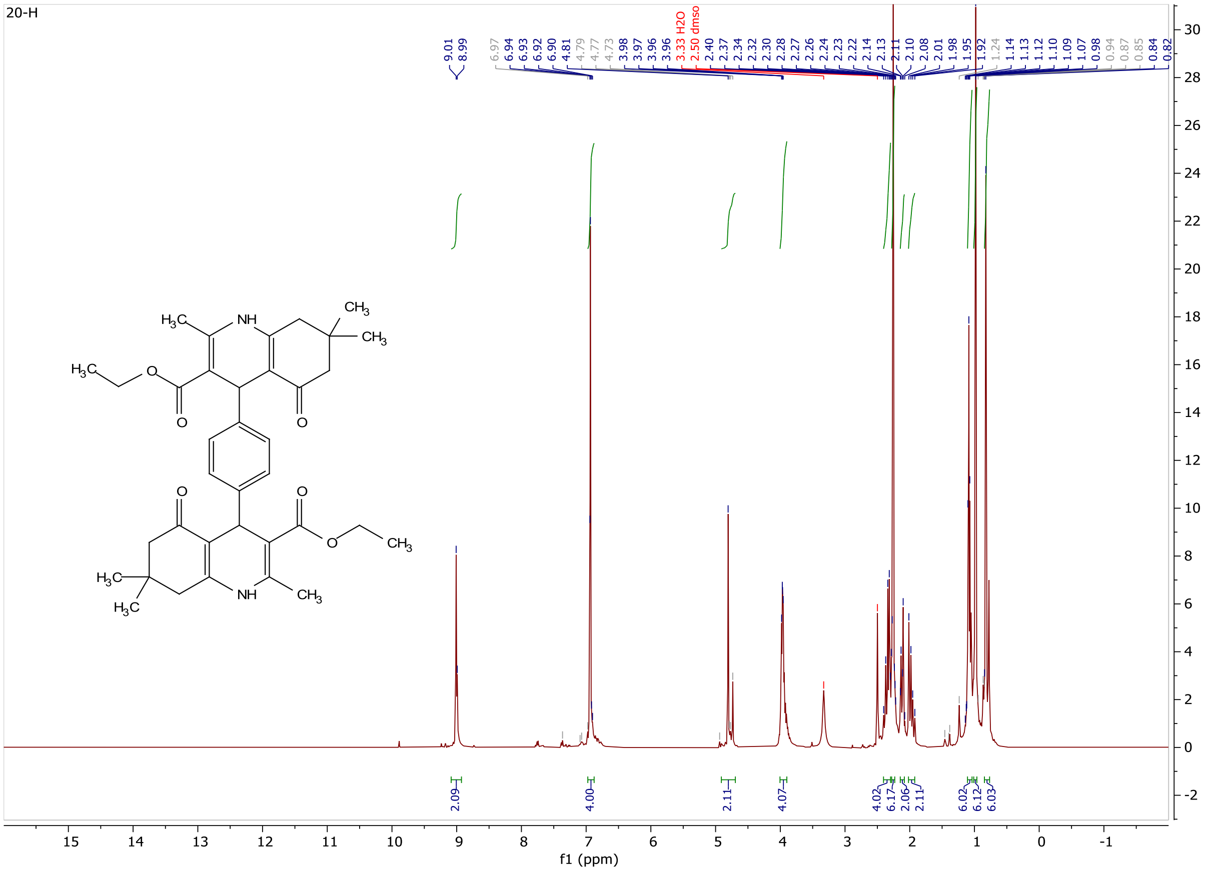

Fig S27. ^{1}H NMR spectrum of 1,4-bis(3-ethoxylcarbonyl-1,4,5,6,7,8-hexahydro-5-oxo-2,7,7-trimethylquinoline-4-yl)benzene.

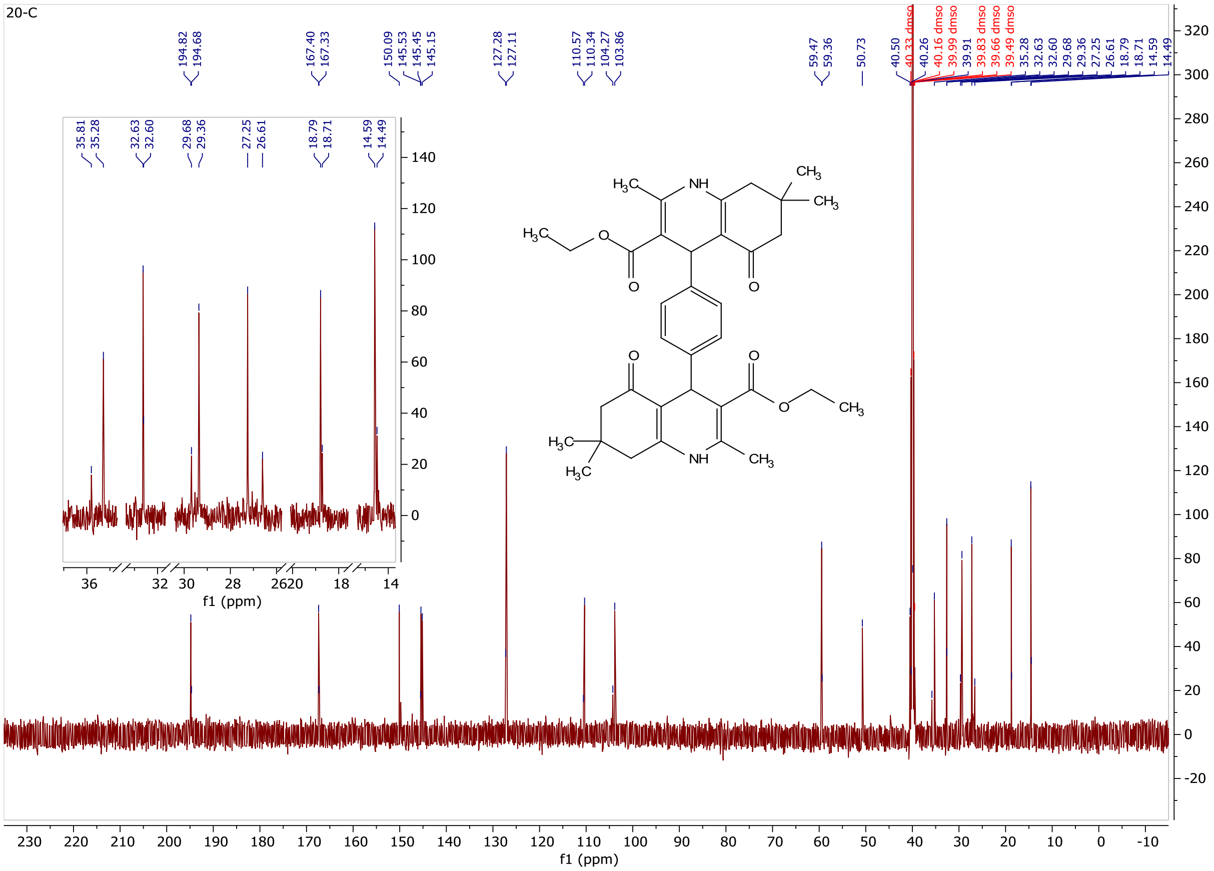

Fig S28. ^{13}C NMR spectrum of 1,4-bis(3-ethoxylcarbonyl-1,4,5,6,7,8-hexahydro-5-oxo-2,7,7-trimethylquinoline-4-yl)benzene.

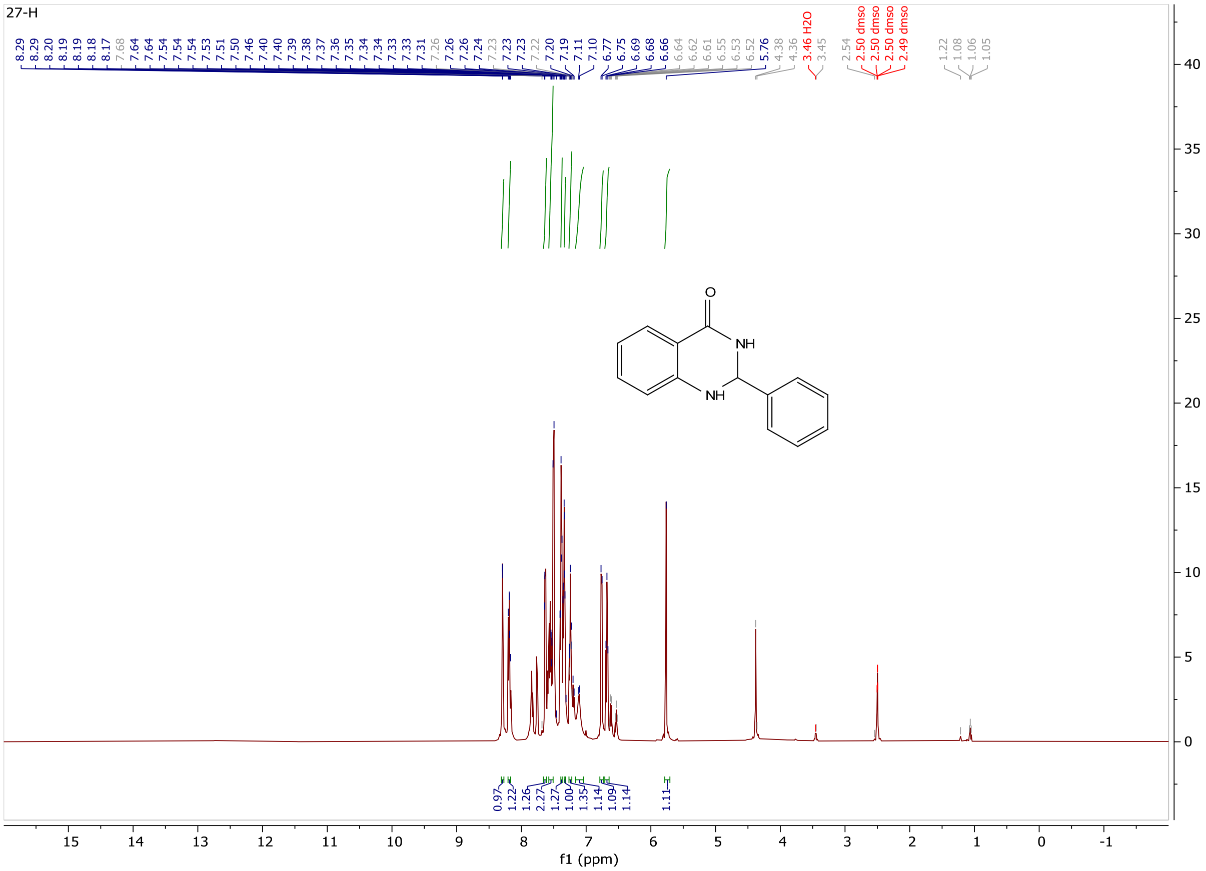

Fig S29. ^{1}H NMR spectrum of 2-Phenyl-2,3-dihydroquinazolin-4(1H)-one.

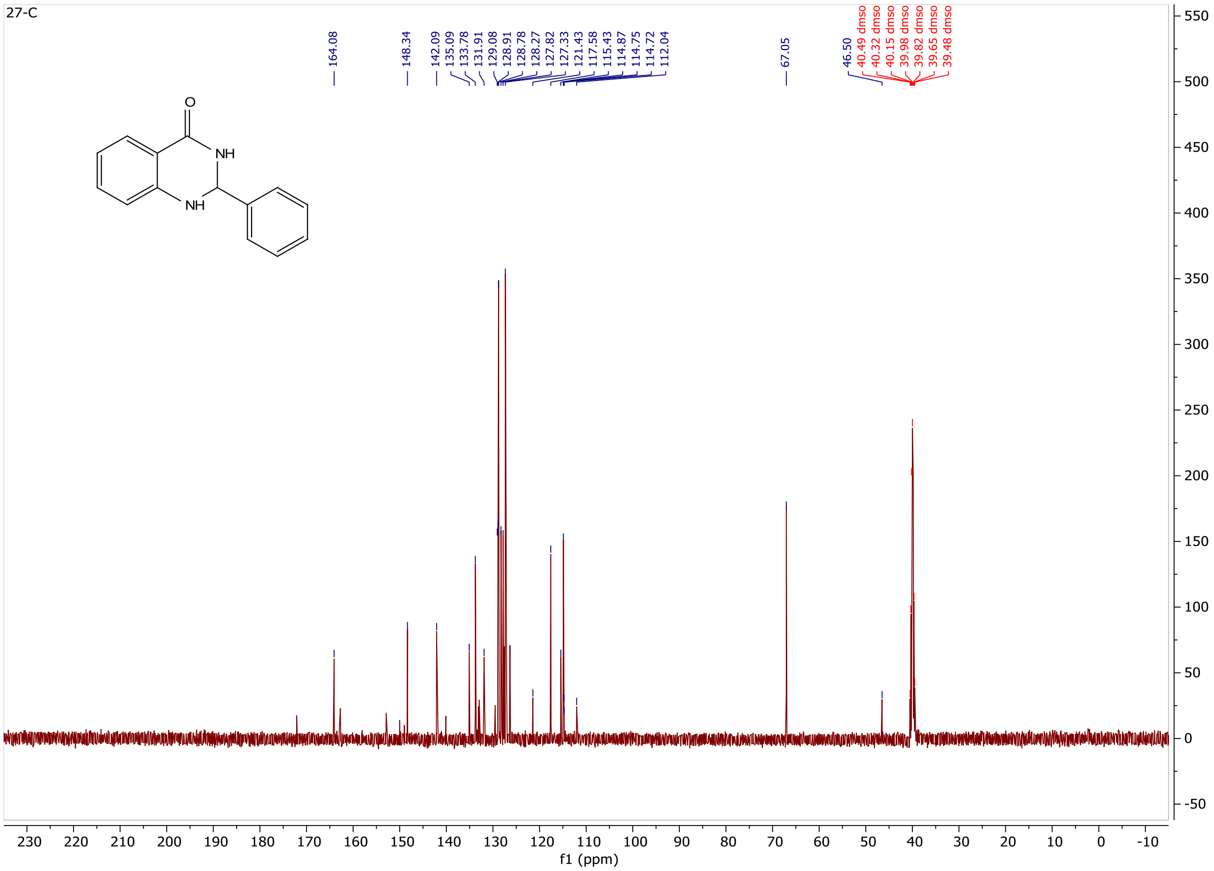

Fig S30. ^{13}C NMR spectrum of 2-Phenyl-2,3-dihydroquinazolin-4(1H)-one.

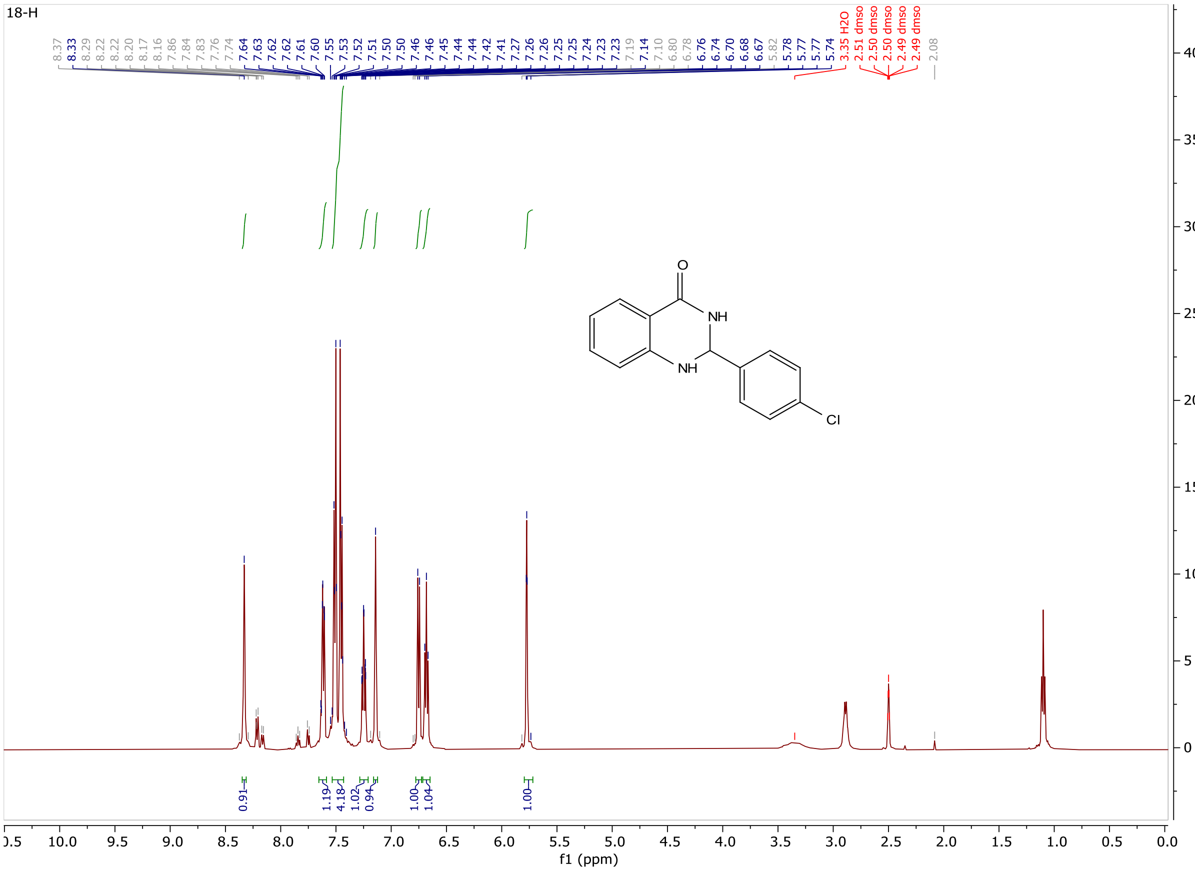

Fig S31. ^{1}H NMR spectrum of 2-(4-chlorophenyl)-2, 3-dihydroquinazolin-4(1H)-one.

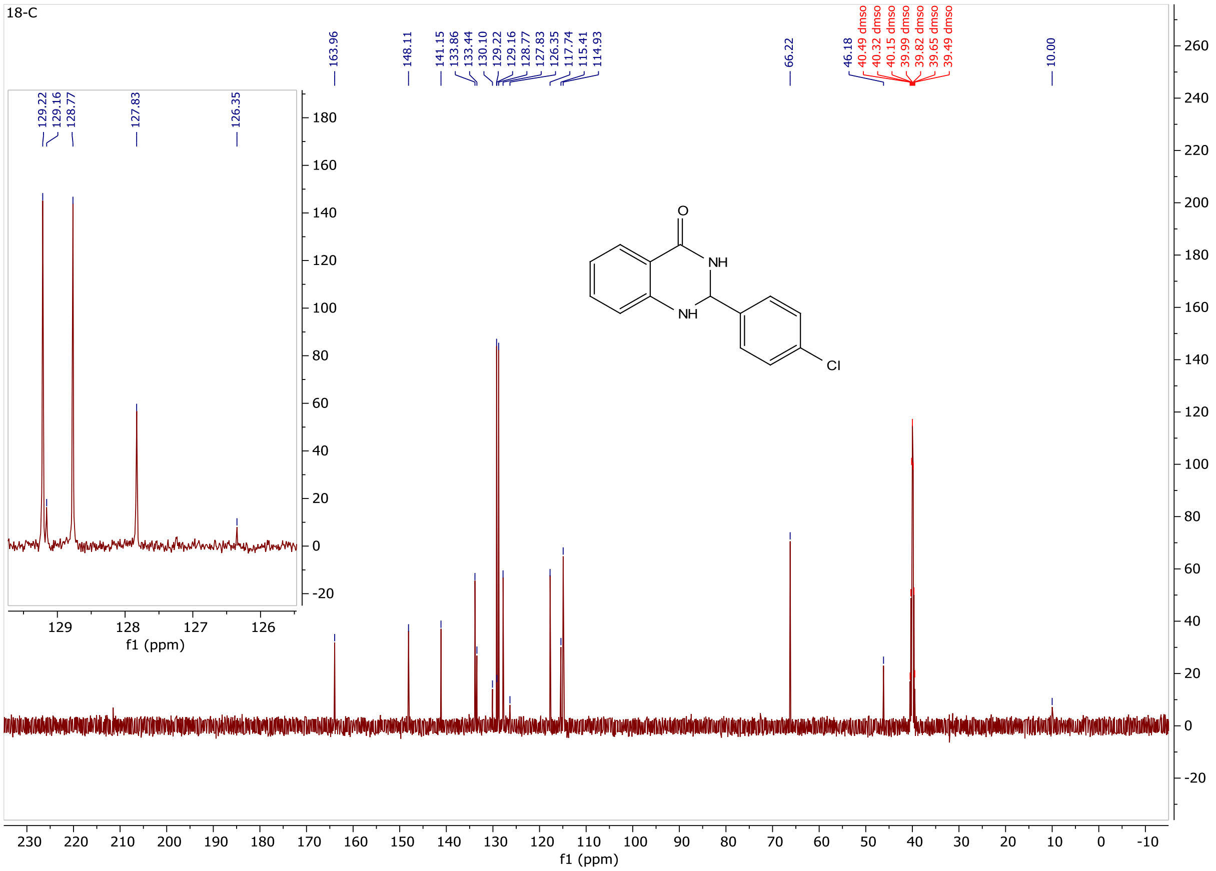

Fig S32. ^{13}C NMR spectrum of 2-(4-chlorophenyl)-2, 3-dihydroquinazolin-4(1H)-one.

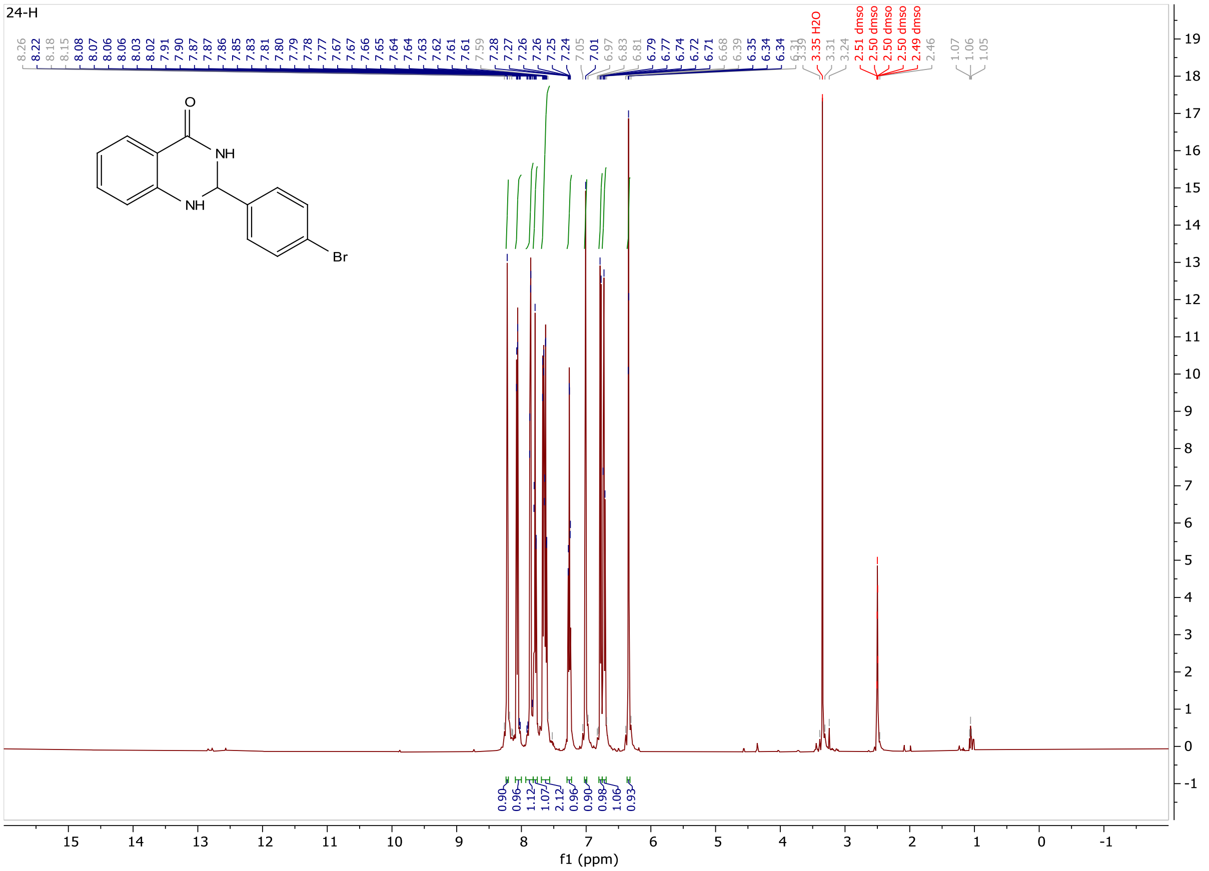

Fig S33. ^{1}H NMR spectrum of 2-(4-Bromophenyl)-2,3-dihydroquinazolin-4(1H)-one.

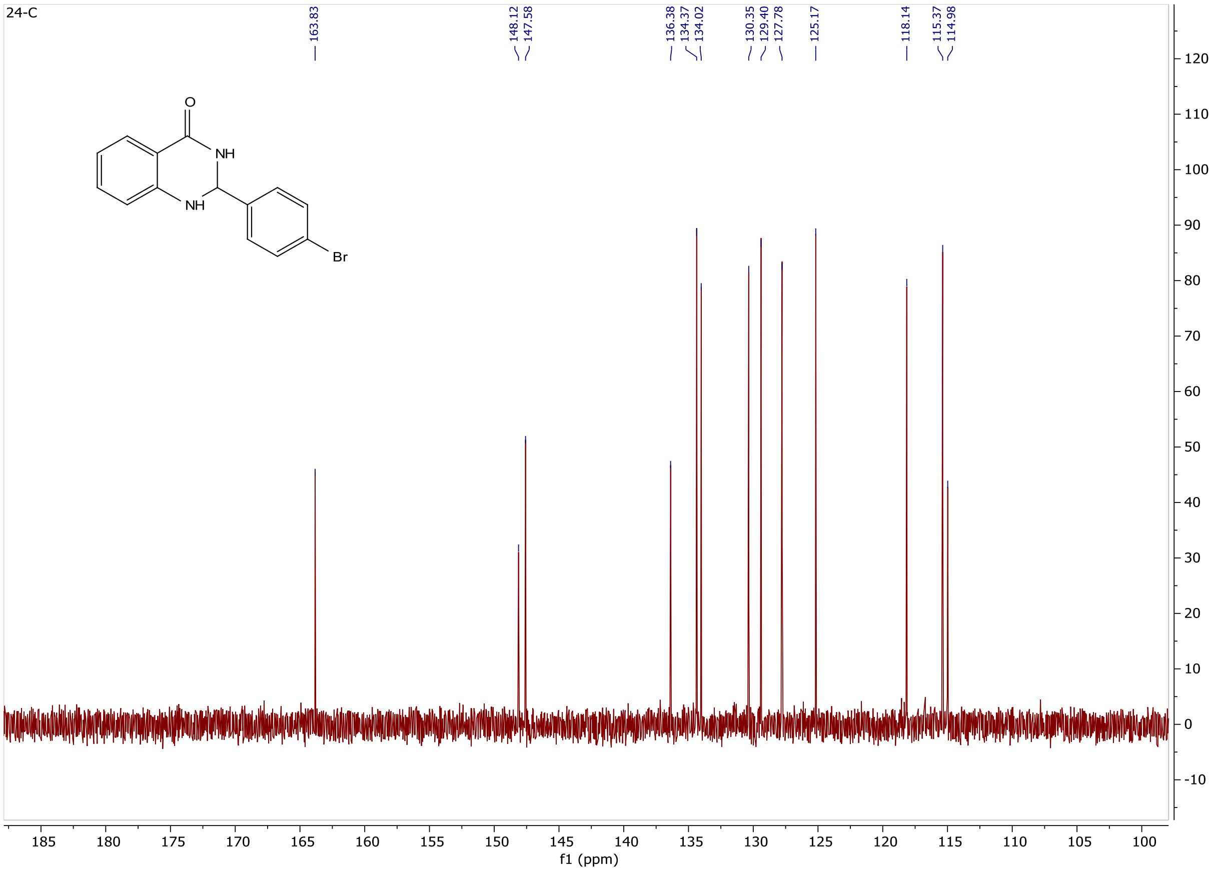

Fig S34. ^{13}C NMR spectrum of 2-(4-Bromophenyl)-2,3-dihydroquinazolin-4(1H)-one.

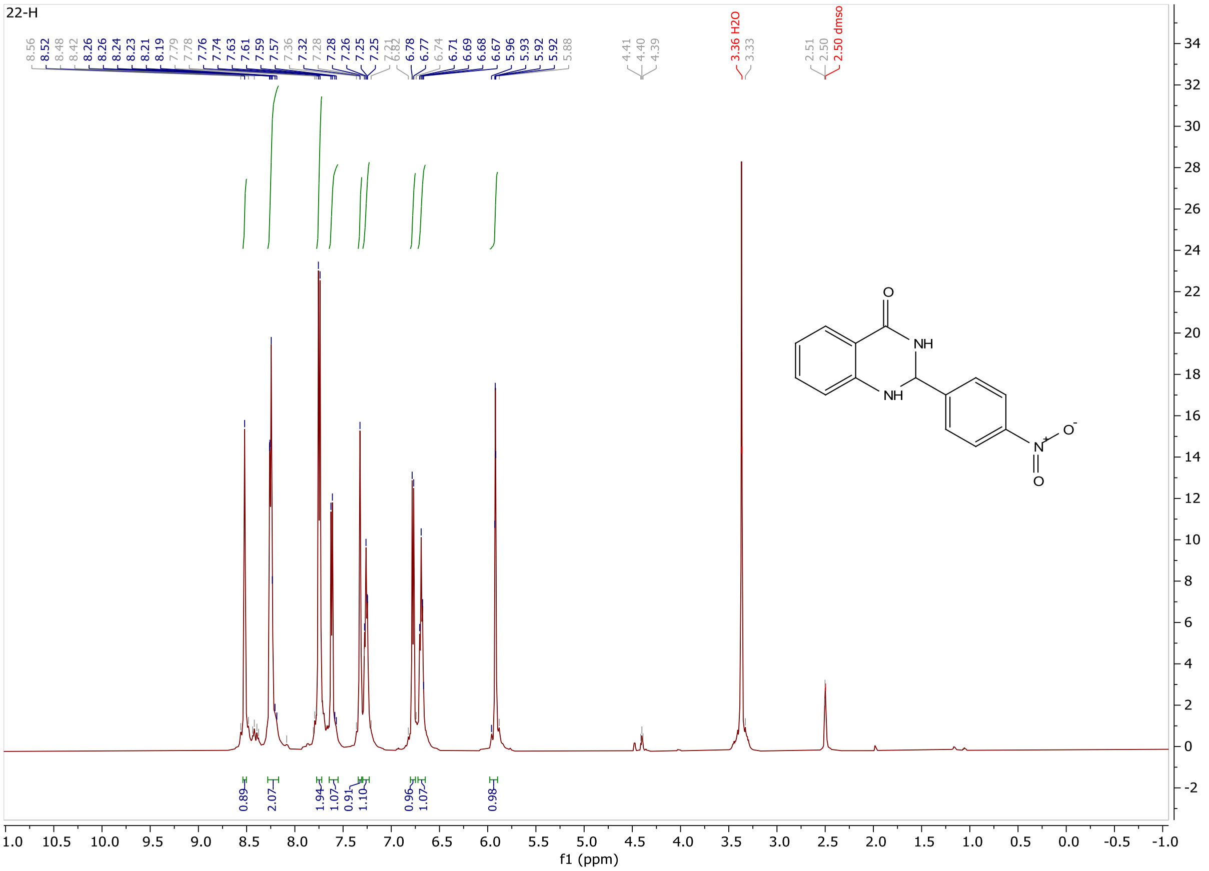

Fig S35. ^{1}H NMR spectrum of 2-(4-Nitrophenyl)-2,3-dihydroquinazolin-4(1H)-one.

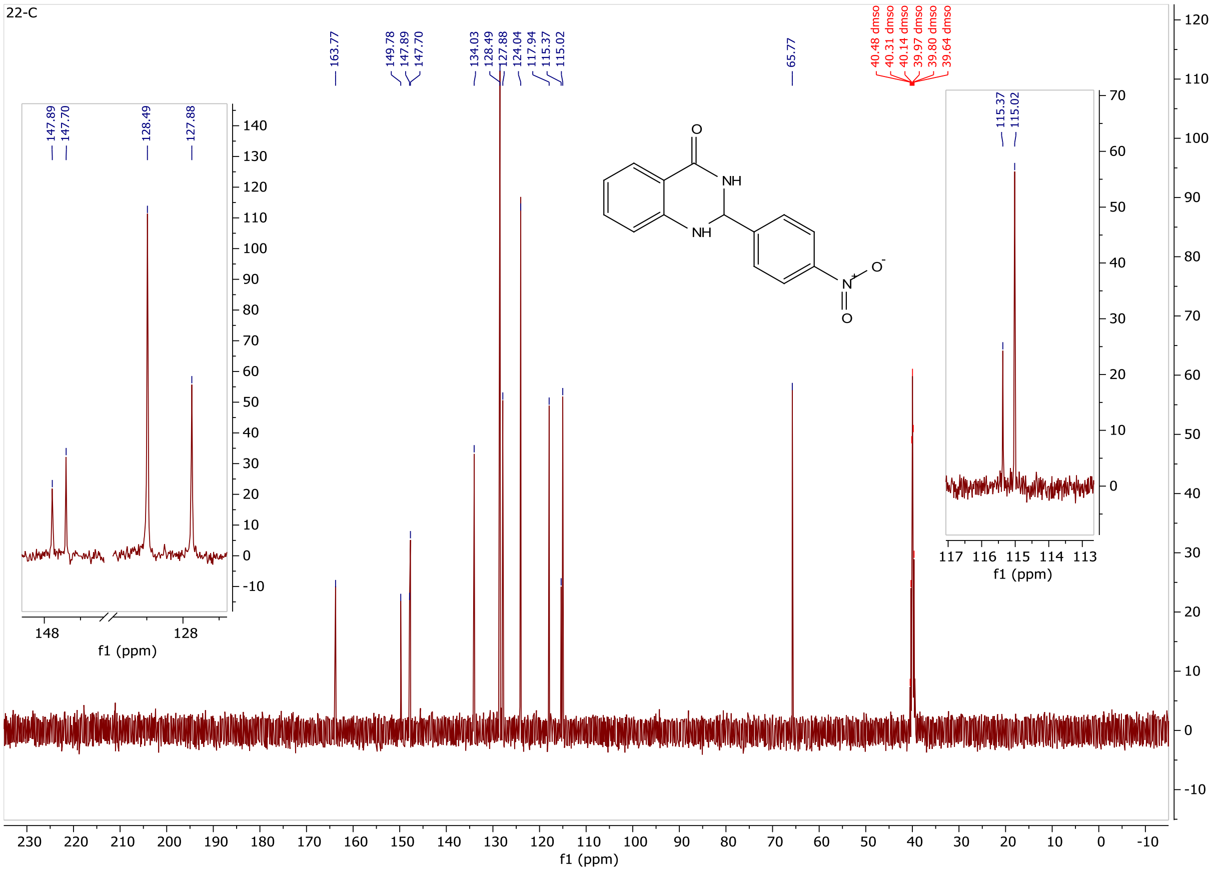

Fig S36. ^{13}C NMR spectrum of 2-(4-Nitrophenyl)-2,3-dihydroquinazolin-4(1H)-one.

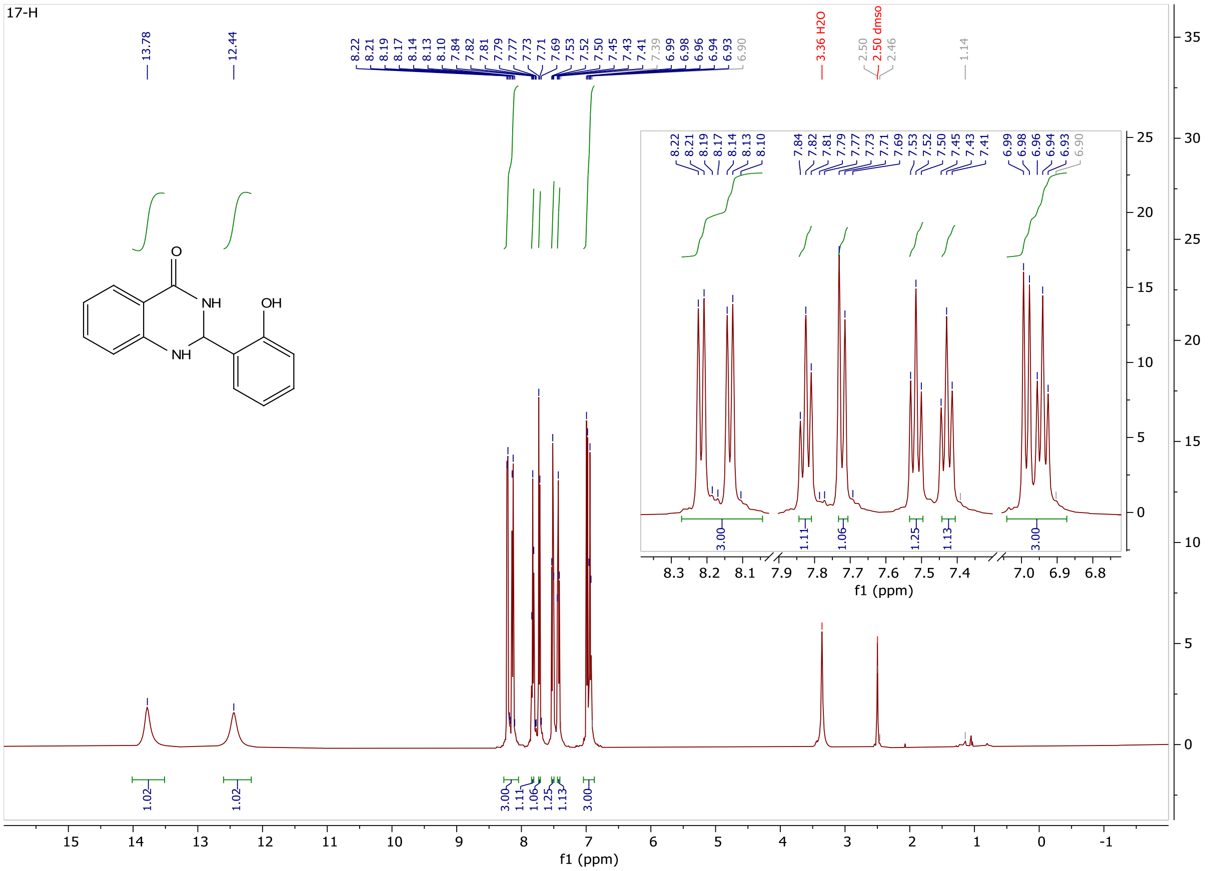

Fig S37. ^{1}H NMR spectrum of 2-(2-Hydoxyphenyl)-2,3-dihydroquinazolin-4(1H)-one.

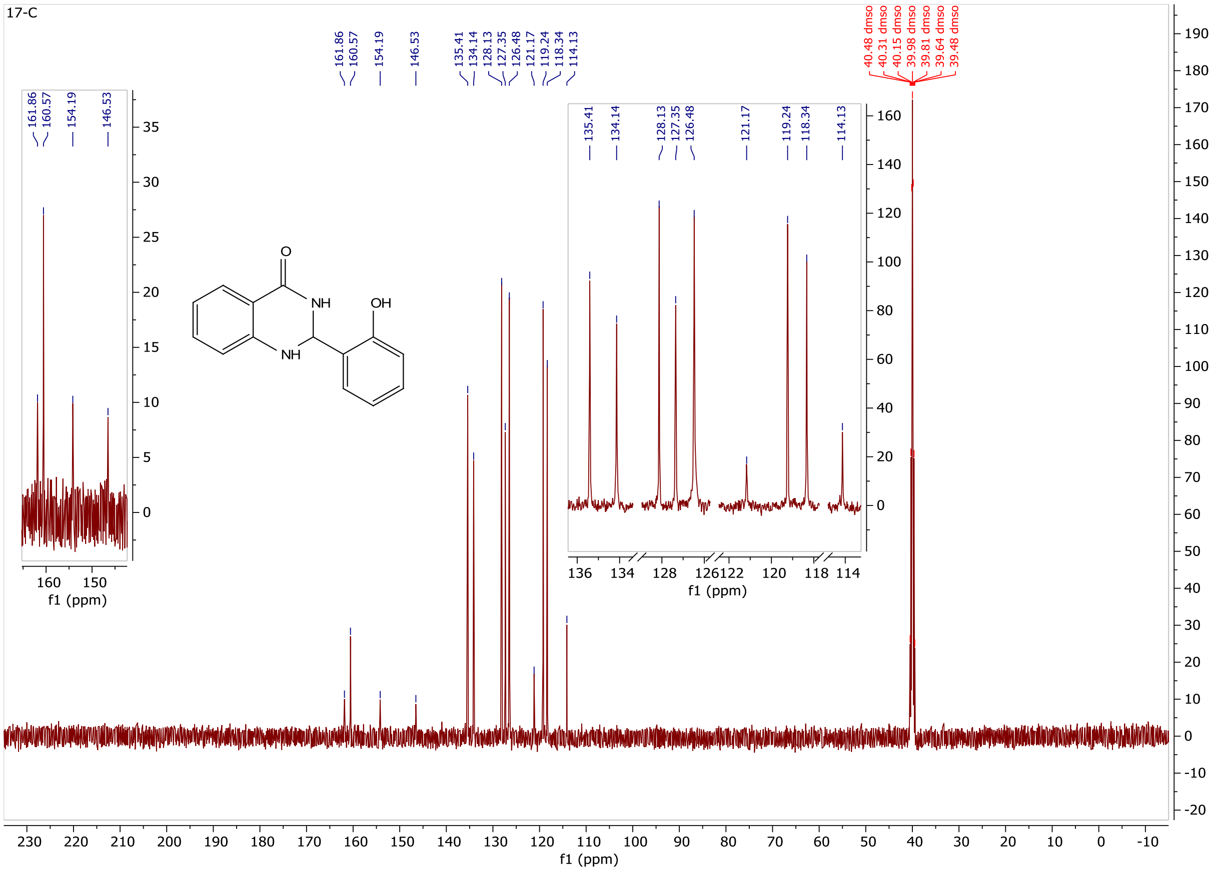

Fig S38. ^{13}C NMR spectrum of 2-(2-Hydoxyphenyl)-2,3-dihydroquinazolin-4(1H)-one.

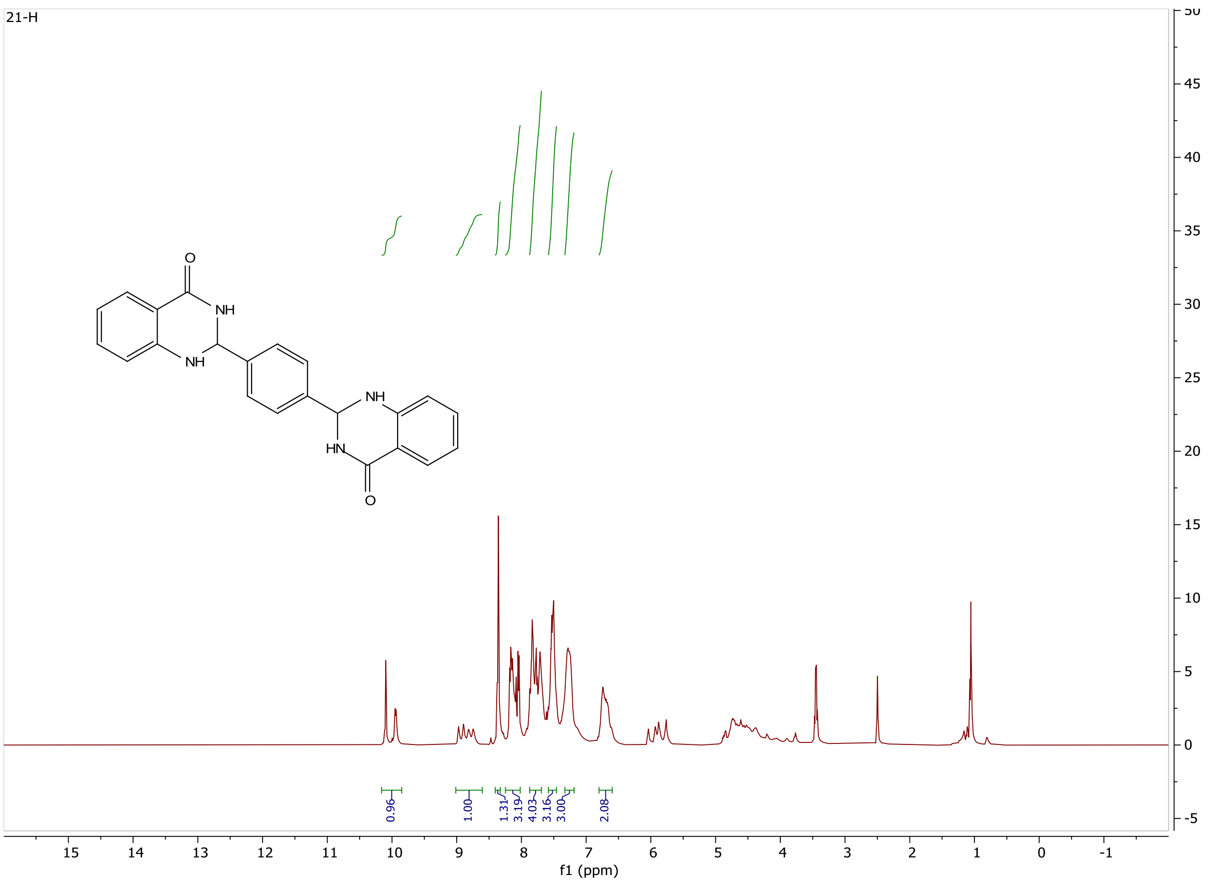

Fig S39. ^{1}H NMR spectrum of 2,2'-(1,4-phenylene)bis(2,3-dihydroquinazolin-4(1H)-one).

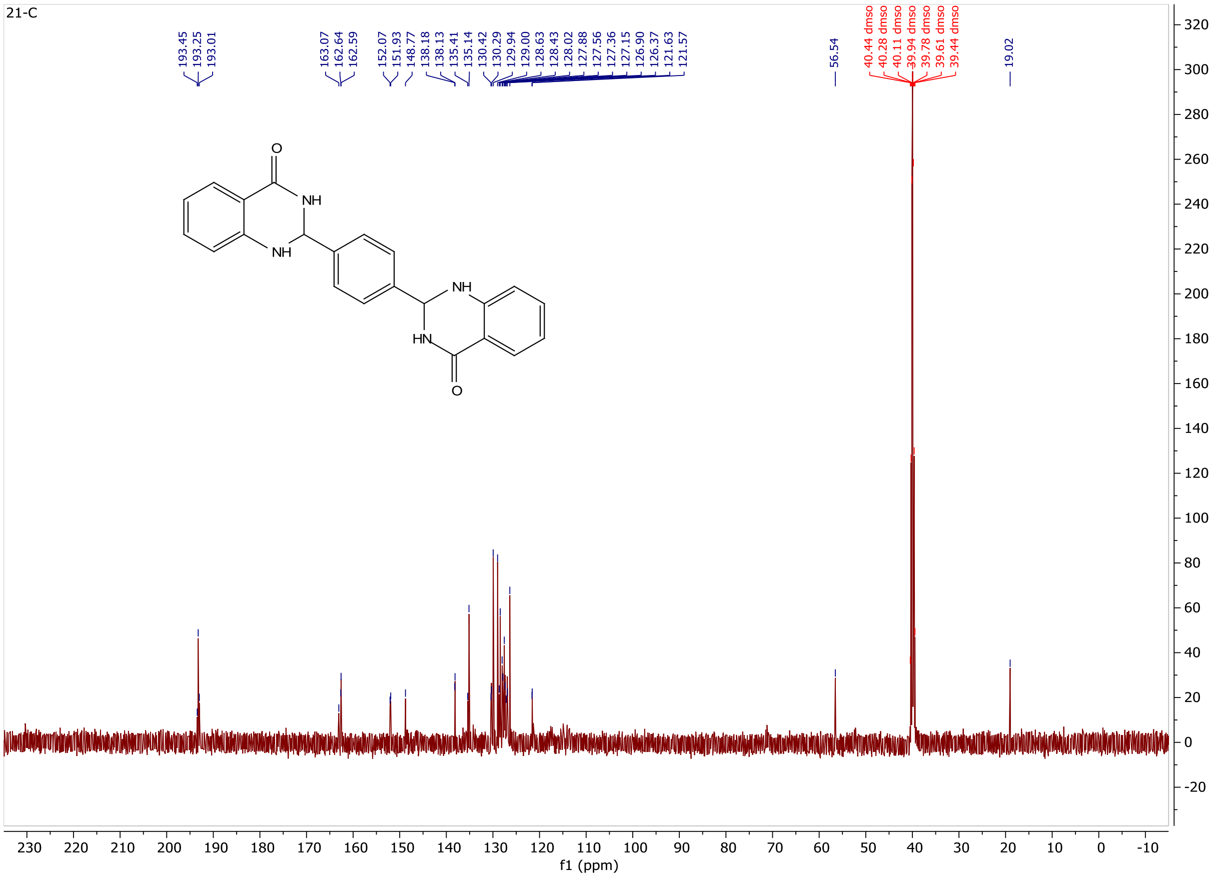

Fig S40. ^{13}C NMR spectrum of 2,2'-(1,4-phenylene)bis(2,3-dihydroquinazolin-4(1H)-one).

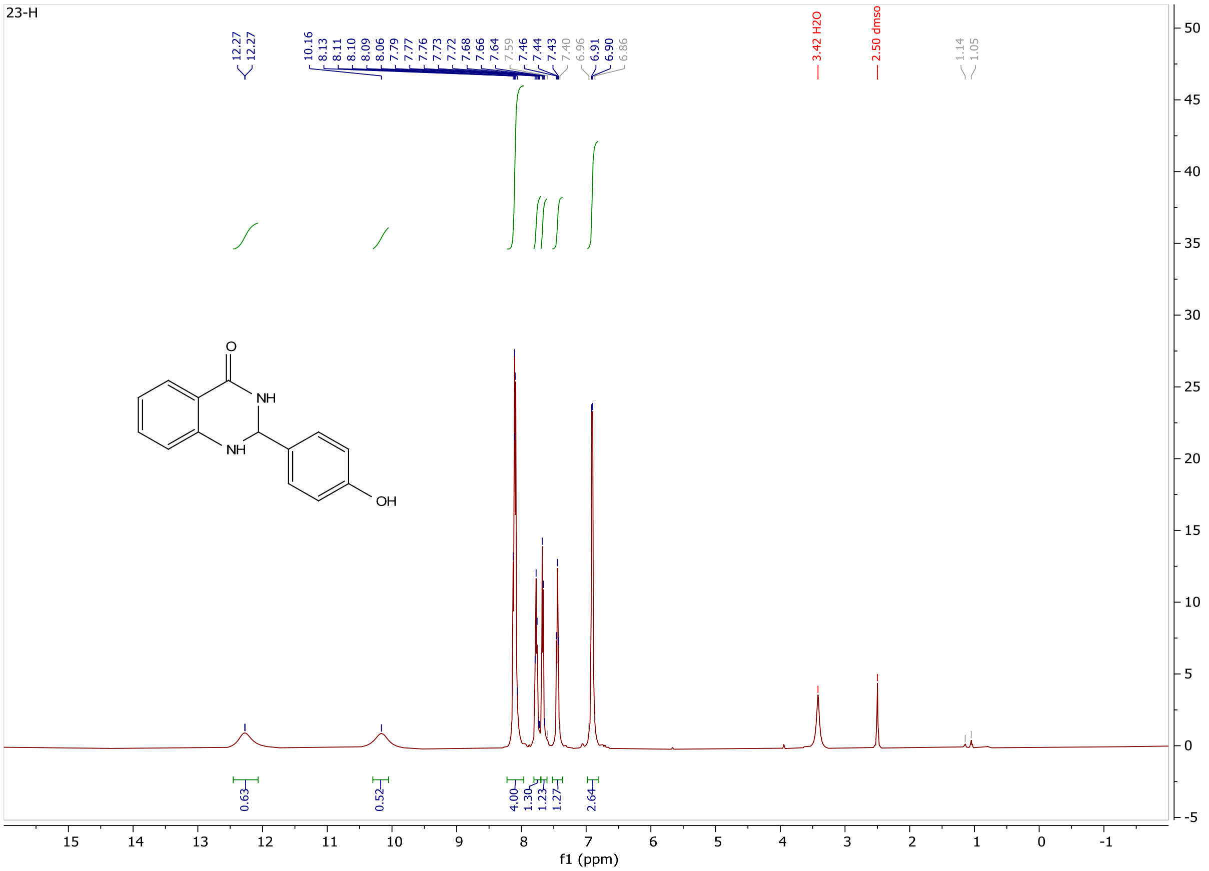

Fig S41. ^{1}H NMR spectrum of 2-(4-Hydroxy)-2, 3-dihydroquinazolin-4(1H)-one.

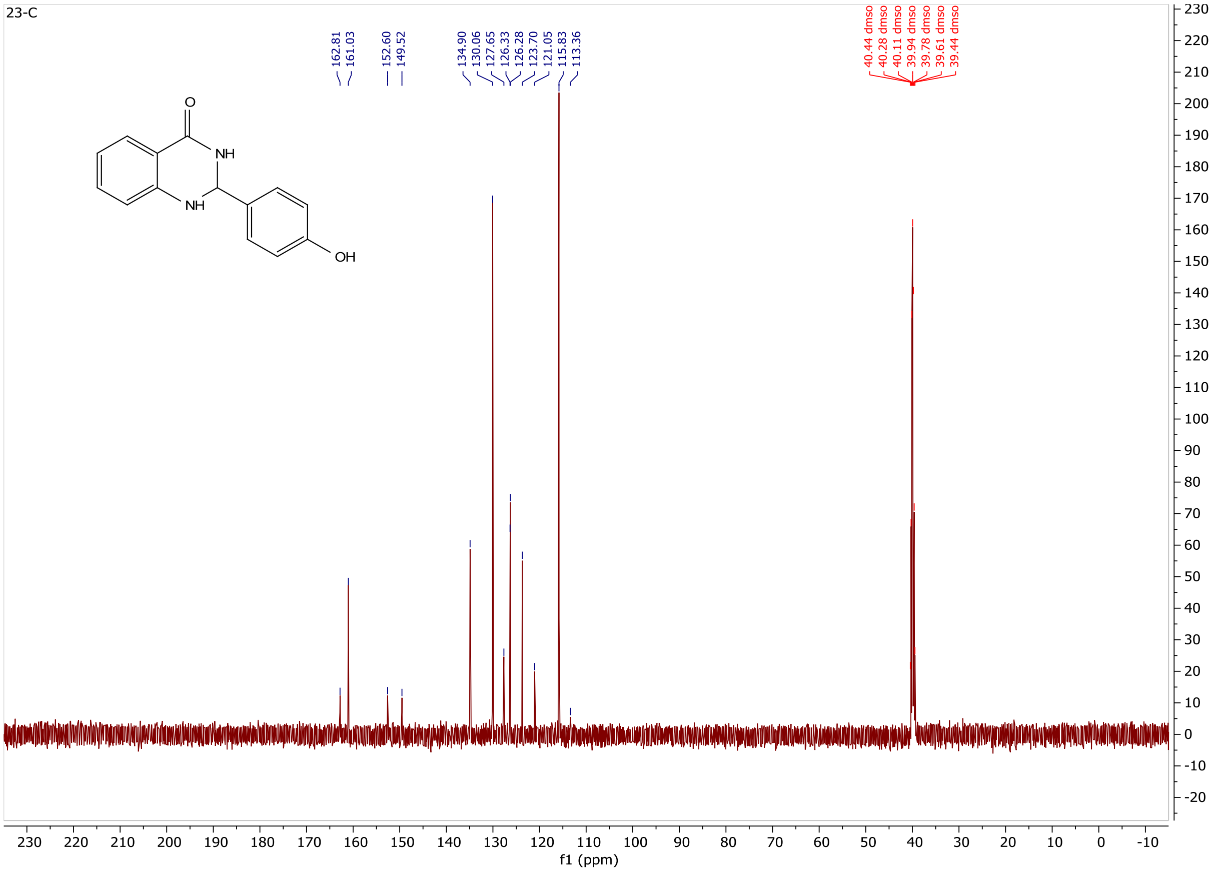

Fig S42. ^{13}C NMR spectrum of 2-(4-Hydroxy)-2, 3-dihydroquinazolin-4(1H)-one.

Supplementary information

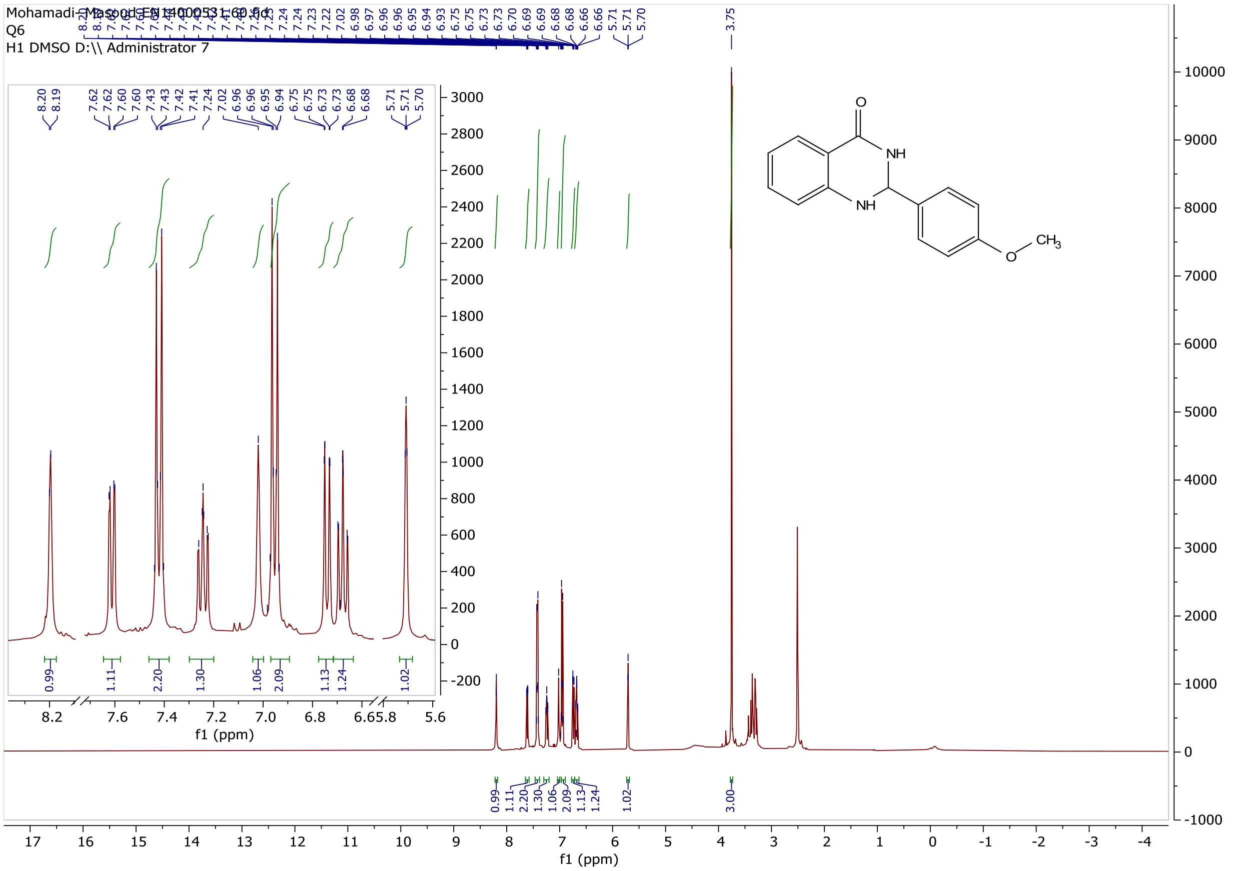

Fig S43. ^{1}H NMR spectrum of 2-(4-Methoxyphenyl)-2, 3-dihydroquinazolin-4(1H)-one.

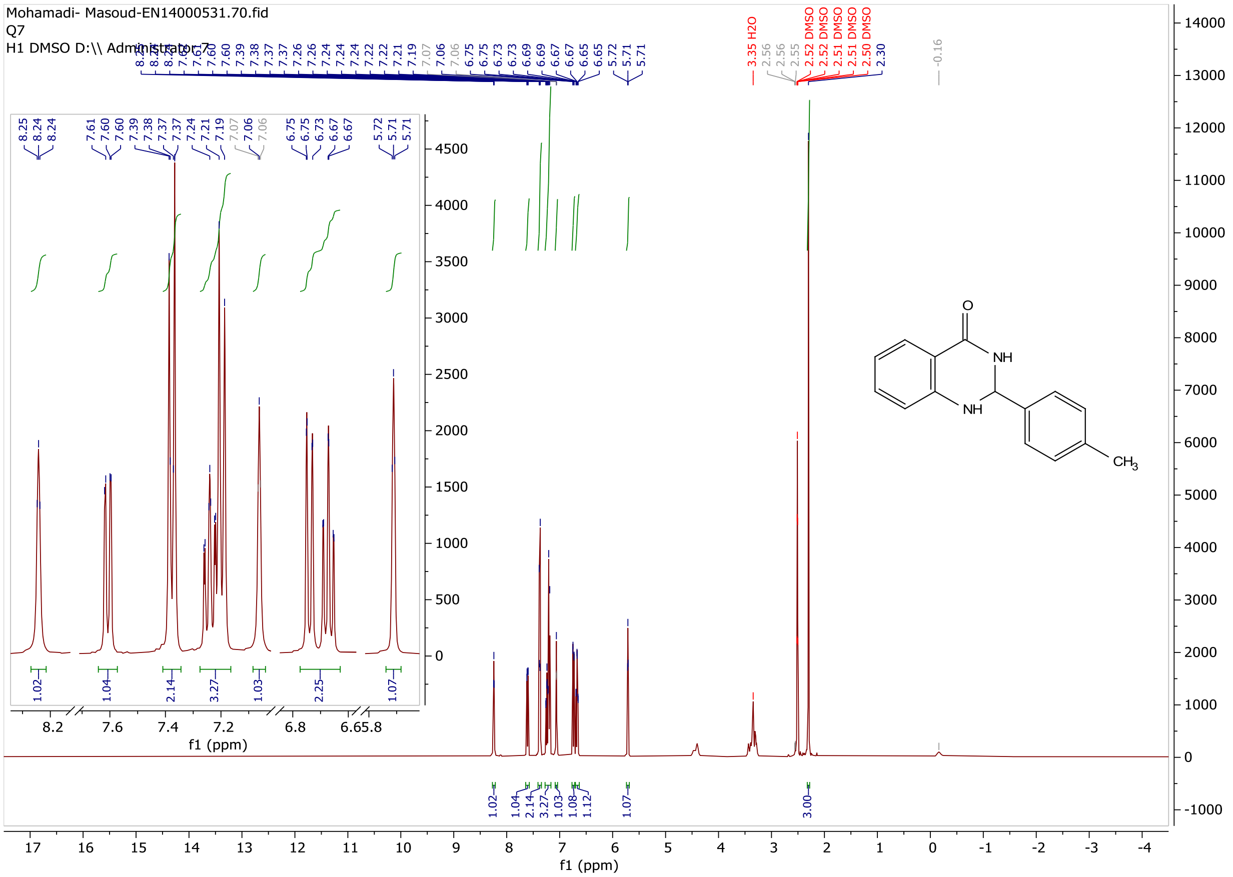

Fig S44. ^{1}H NMR spectrum of 2-(4-Tolyl)-2, 3-dihydroquinazolin-4(1H)-one.

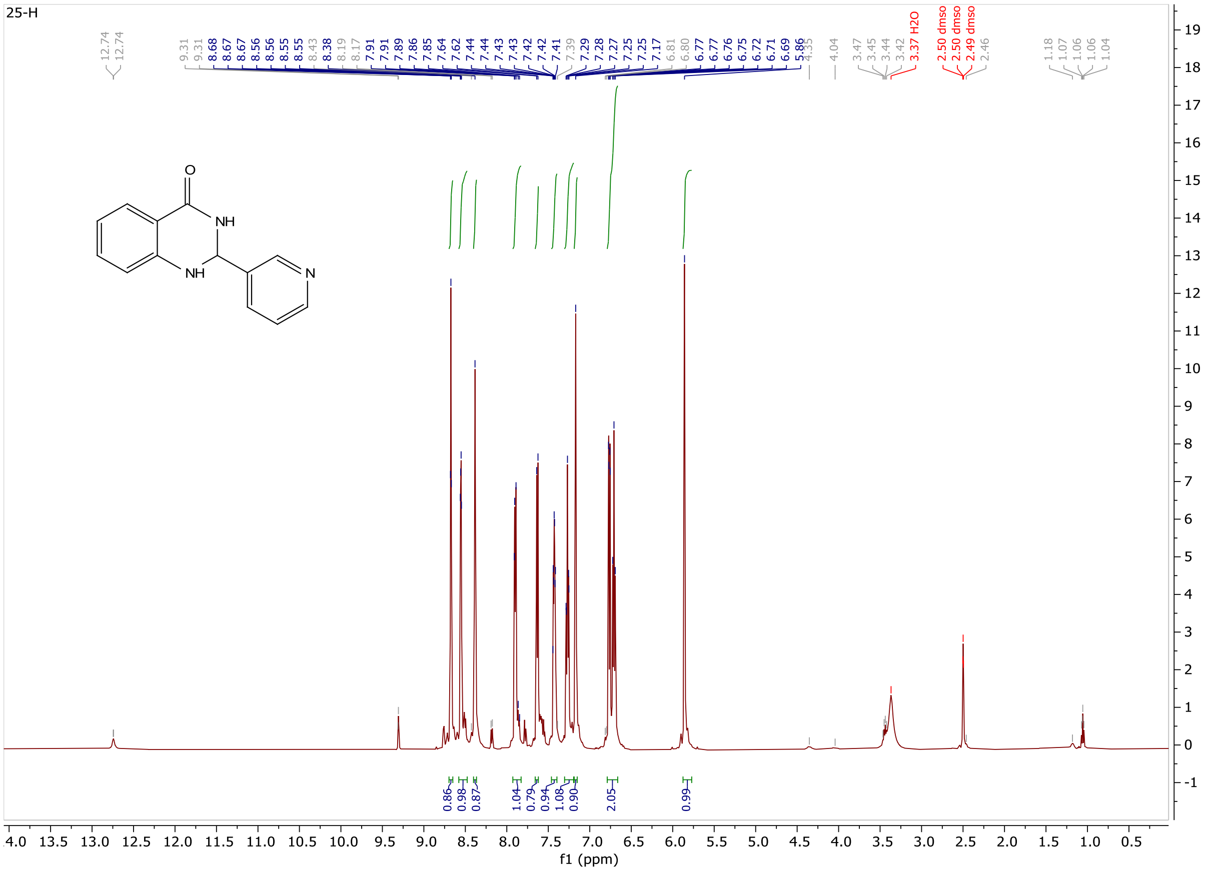

Fig S45. ^{1}H NMR spectrum of 2-(pyridin-3-yl)-2,3-dihydroquinazolin-4(1H)-one.

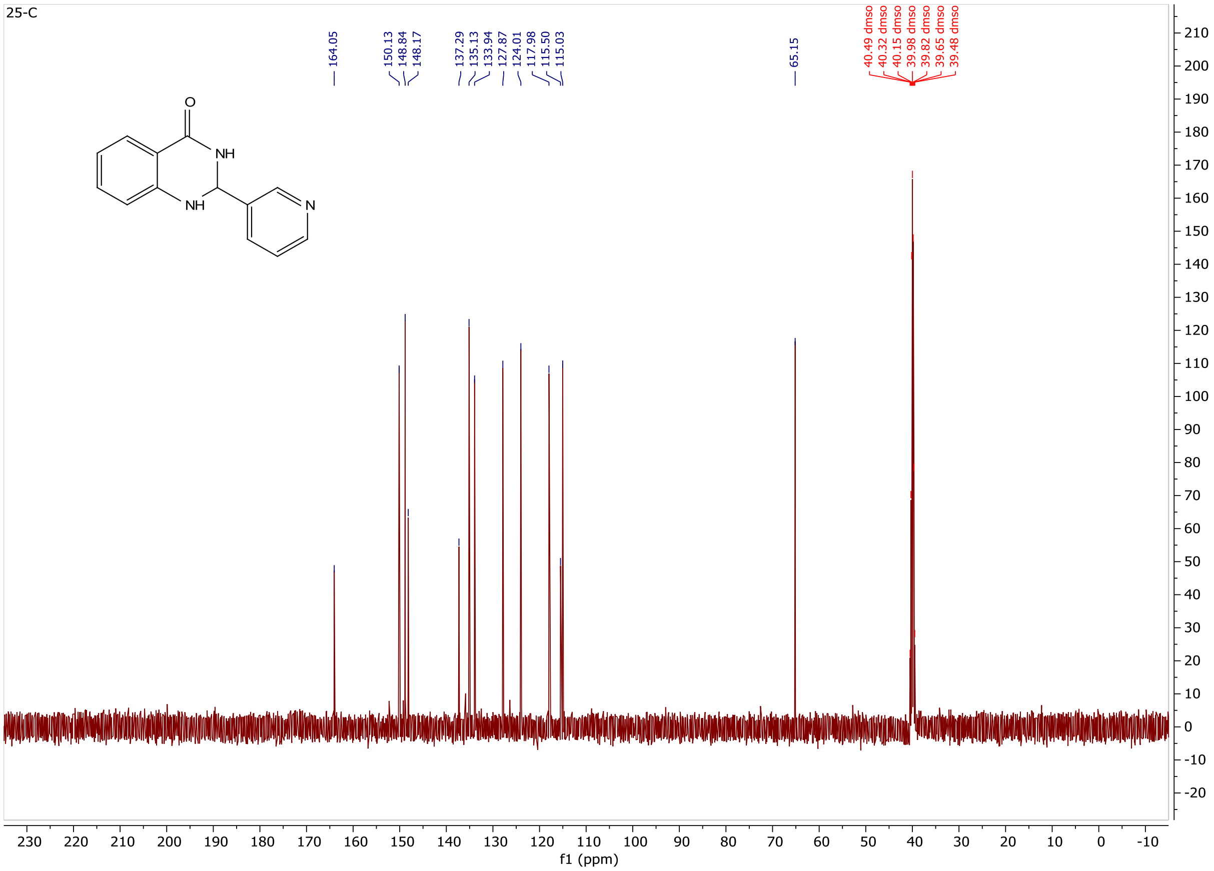

Fig S46. ^{13}C NMR spectrum of 2-(pyridin-3-yl)-2,3-dihydroquinazolin-4(1H)-one.

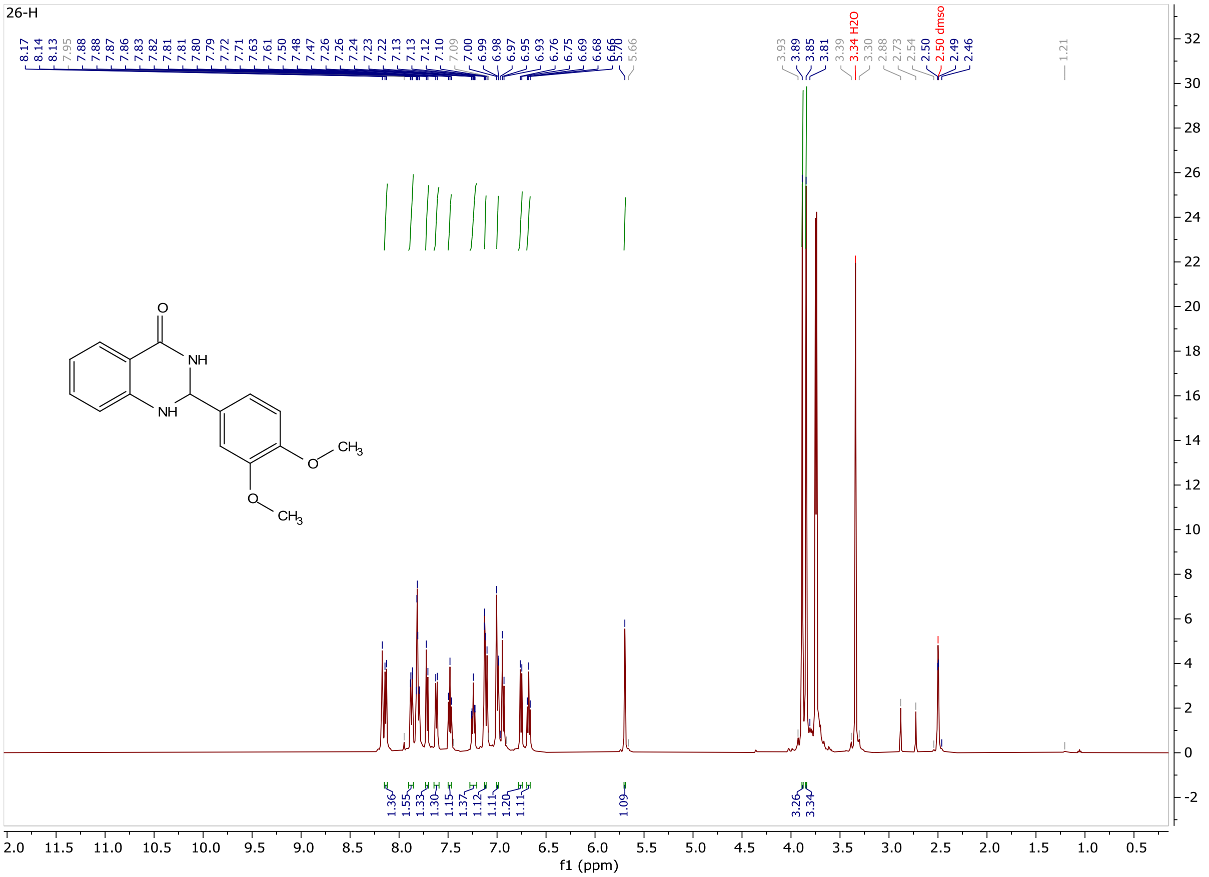

Fig S47. ^{1}H NMR spectrum of 2-(3,4-dimethoxyphenyl)-2,3-dihydroquinazolin-4(1H)-one.

Supplementary information

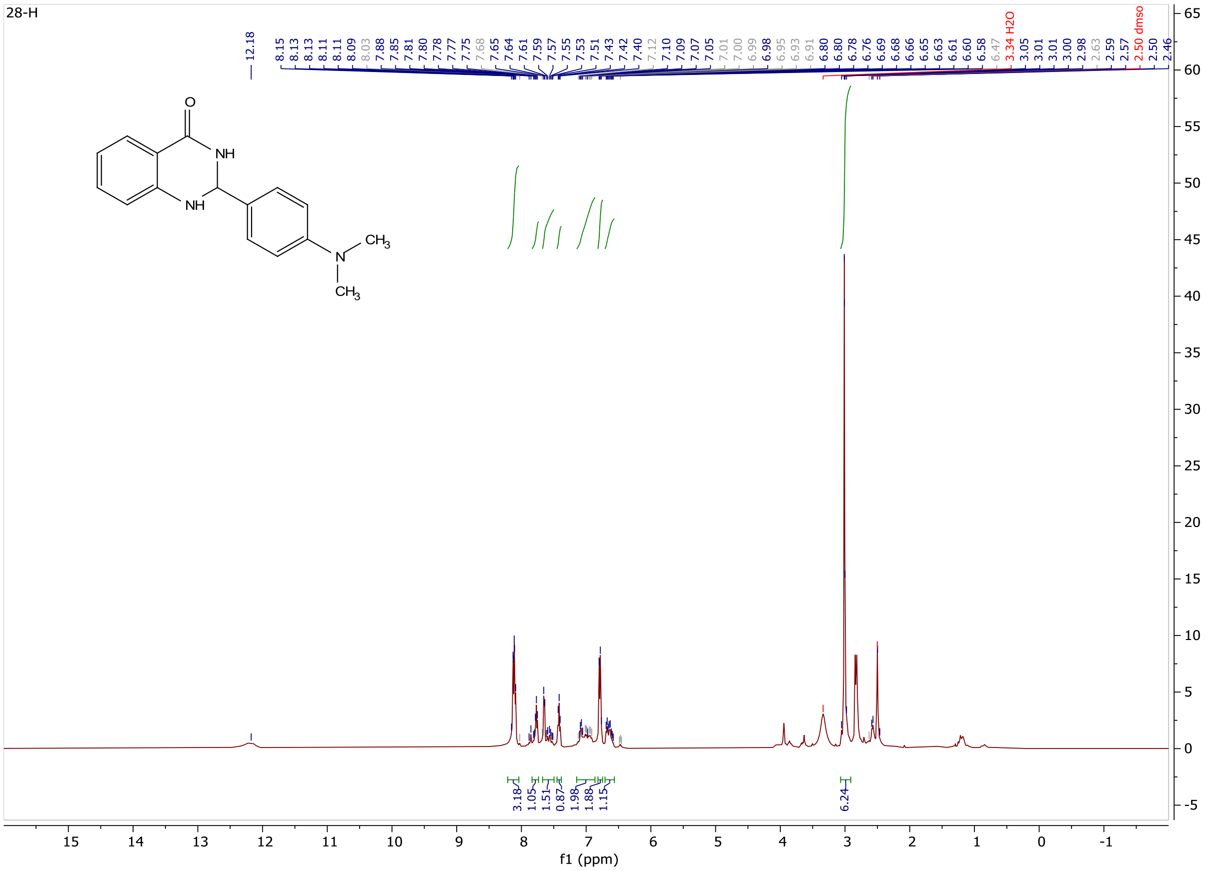

Fig S48. ^{1}H NMR spectrum of 2-(4-(dimethylamino)phenyl)-2,3-dihydroquinazolin-4(1H)-one.

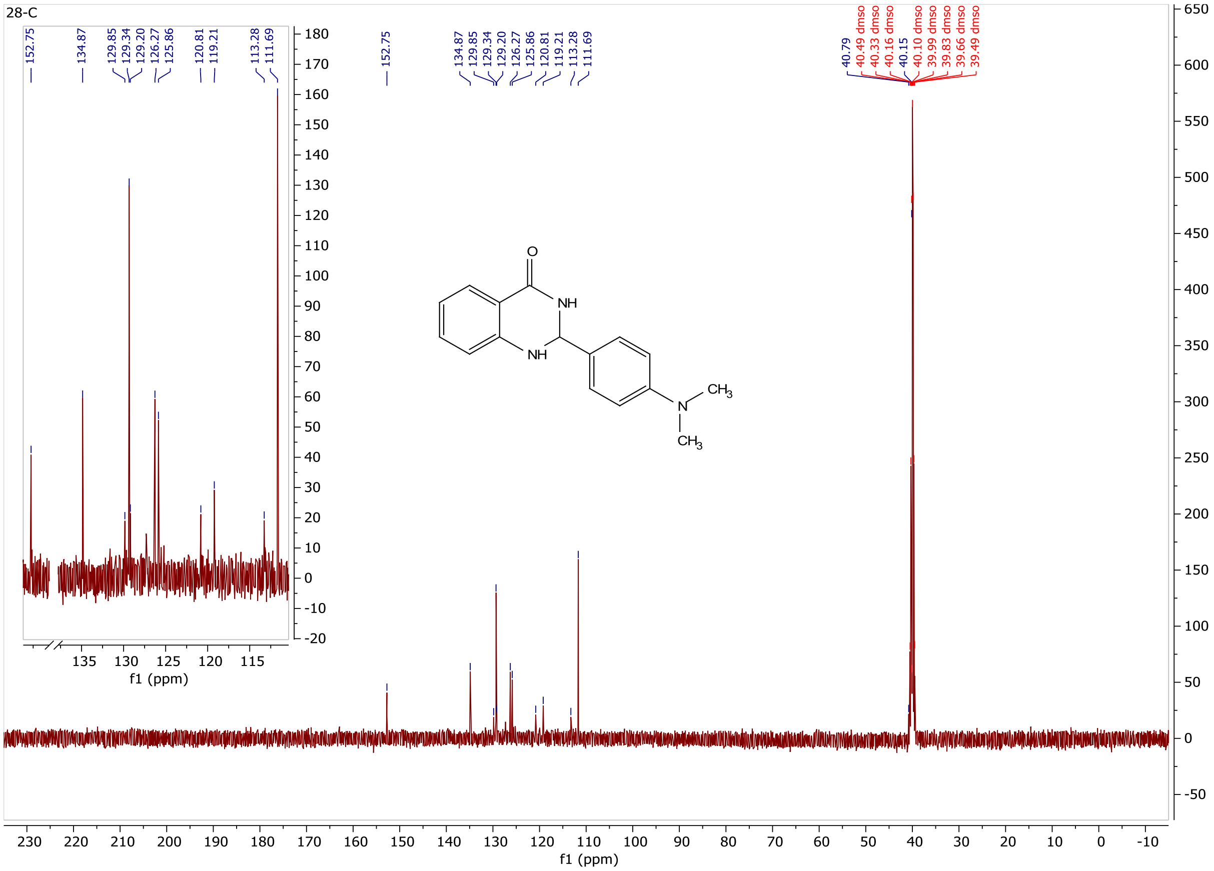

Fig S49. ^{13}C NMR spectrum of 2-(4-(dimethylamino)phenyl)-2,3-dihydroquinazolin-4(1H)-one.
